# First-in-human study of IM156, a novel potent biguanide oxidative phosphorylation (OXPHOS) inhibitor, in patients with advanced solid tumors

**DOI:** 10.1007/s10637-022-01277-9

**Published:** 2022-07-08

**Authors:** Filip Janku, Seung-Hoon Beom, Yong Wha Moon, Tae Won Kim, Young G. Shin, Dong-Seok Yim, Gun Min Kim, Hyo Song Kim, Sun Young Kim, Jae-Ho Cheong, Young Woo Lee, Barb Geiger, Sanghee Yoo, Archie Thurston, Dean Welsch, Marc S. Rudoltz, Sun Young Rha

**Affiliations:** 1 The University of Texas MD Anderson Cancer Center, Houston, TX USA; 2 Yonsei Cancer Center, Yonsei University College of Medicine, Yonsei University Health System, Seoul, South Korea; 3 Song-Dang Institute for Cancer Research, Yonsei University College of Medicine, Seoul, South Korea; 4 Hematology and Oncology, Department of Internal Medicine, CHA Bundang Medical Center, Seongnam, South Korea; 5 Asan Medical Center, University of Ulsan College of Medicine, Seoul, South Korea; 6 Institute of Drug Research and Development, College of Pharmacy, Chungnam National University, Daejeon, South Korea; 7 Department of Pharmacology, College of Medicine, The Catholic University of Korea, Seoul, South Korea; 8 ImmunoMet Therapeutics, Inc, Houston, TX USA; 9 ADME Solutions, Inc, San Diego, CA USA

**Keywords:** Protein complex I inhibitor, Biguanide, Clinical trial, IM156, Cancer

## Abstract

Preclinical models suggest anticancer activity of IM156, a novel biguanide mitochondrial protein complex 1 inhibitor of oxidative phosphorylation (OXPHOS). This first-in-human dose-escalation study enrolled patients with refractory advanced solid tumors to determine the maximum tolerated dose (MTD) or recommended phase 2 dose (RP2D). Eligible patients received oral IM156 every other day (QOD) or daily (QD) and were assessed for safety, dose-limiting toxicities (DLTs), pharmacokinetics, and preliminary signals of efficacy. 22 patients with advanced cancers (gastric, n = 8; colorectal, n = 3; ovarian, n = 3; other, n = 8) received IM156 100 to 1,200 mg either QOD or QD. There were no DLTs. However, 1,200 mg QD was not well tolerated due to nausea; 800 mg QD was determined as the RP2D. The most frequent treatment-related AEs (TRAEs) were nausea (n = 15; 68%), diarrhea (n = 10; 46%), emesis (n = 9; 41%), fatigue (n = 4; 18%) and abdominal pain, constipation, and blood lactate increased (n = 2 each; 9%). Grade 3 nausea (n = 3; 14%) was the only grade ≥ 3 TRAE. Plasma exposures increased dose proportionally; mean Day 27 area under the curve (AUC_0-24_) values were higher following QD administration compared to the respective QOD regimen. Stable disease (SD), observed in 7 (32%) patients (confirmed in 2 [9%]), was the best response. To our knowledge, this is the first phase 1 study of an OXPHOS inhibitor that established a RP2D for further clinical development in cancer. Observed AEs of IM156 were manageable and SD was the best response.

**Supplementary Information:**

The online version contains supplementary material available at 10.1007/s10637-022-01277-9.

## Introduction

Oxidative phosphorylation (OXPHOS) is an important source of energy and metabolic precursors for tumor cells and offers an attractive target for development of anticancer therapies [1]. Oxidative phosphorylation can be targeted by inhibition of mitochondrial protein complex 1 (PC1), a critical component and the first step in the electron transport chain that binds and oxidizes nicotinamide adenine dinucleotide hydride (NADH) before the subsequent transfer of electrons resulting in formation of adenosine triphosphate (ATP) [2]. Preclinical experiments in cancer demonstrated that biguanides such as metformin, commonly used to treat Type 2 diabetes mellitus (DM), and phenformin, also used to treat Type 2 DM but withdrawn from the market due to the risk for lactic acidosis, can inhibit OXPHOS via inhibition of PC1 [3]. Despite most cancers having increased rates of glycolysis, they also require OXPHOS [1]. Therefore, energetic stress in cancer cells due to inhibition of OXPHOS by biguanides may result in cancer cell death [4–6]. Metformin demonstrated anticancer activity in preclinical in vitro and in vivo models; however, an important caveat was that many in vitro models with anticancer activity used metformin concentrations higher than those which can be achieved in the plasma of patients treated with conventional doses [7, 8]. Indeed, most clinical studies and randomized trials with metformin failed to show meaningful anticancer activity in patients with advanced cancers [9, 10]. Nevertheless, since many *in vivo* cancer models demonstrated remarkable antineoplastic activity of biguanides, the development of novel biguanides with improved pharmacokinetic (PK) and toxicity profiles would potentially be of clinical benefit [4, 11–13].

IM156 is a novel biguanide that inhibits PC1 [3]. Metformin is relatively hydrophilic and thus requires active transport (e.g., through OCT1) to enter cells. IM156, on the other hand, is more hydrophobic than metformin and thus potentially more bioavailable to cancer cells. In addition, at equal concentrations IM156 was more potent at decreasing the oxygen consumption rate (OCR) of tumor cells compared to phenformin and metformin, and in reducing cellular ATP production versus phenformin without an increase in the extracellular acidification rate (ECAR) (Immunomet – Data on File). In addition, IM156 inhibits mTOR through AMPK activation resulting in inhibition of cancer cell growth and proliferation (Immunomet – Data on File). Furthermore, preclinical in vivo models demonstrated anticancer activity of IM156 in glioblastoma, gastric cancer and *EGFR*-mutated lung cancer [14]. Therefore, we designed a first-in-human dose-escalation study to determine the maximum tolerated dose (MTD) or recommended phase 2 dose (RP2D) and to evaluate the safety, tolerability, pharmacokinetics (PK), exploratory pharmacodynamic (PD) markers, and early signals of efficacy of IM156 in patients with advanced solid tumors refractory to standard therapies.

## Methods

### Study design

This multicenter first-in-human dose-escalation phase 1 study (clinicaltrials.gov identifier: NCT03272256) was designed to determine the MTD or RP2D and to evaluate the safety and tolerability of IM156. Secondary endpoints included PK characteristics, efficacy assessment (objective response rate [ORR], disease control ﻿rate [DCR], duration of response [DoR] and progression-free survival [PFS]). Furthermore, exploratory objectives included identification and profiling of IM156 metabolites, evaluations of exploratory PD markers of IM156, and evaluations of any potential relationship between putative biomarkers and safety/efficacy parameters of IM156. The study was conducted in accordance with Good Clinical Practice, the International Council for Harmonisation, and guidelines governing clinical study conduct and the ethical principles originating from the Declaration of Helsinki. The study was approved by each Institutional Review Board and patients provided written informed consent. Patients were enrolled at three sites in South Korea.

The initial dose and schedule of IM156 was chosen based on animal toxicology studies. A dog model suggested the possibility for drug accumulation; to ensure patient safety, the initial schedule was every other day (QOD; Table 1) and the starting dose of IM156 was 100 mg orally (PO). After enrollment of cohort 5 (1,200 mg QOD), the safety and PK data supported a switch to daily (QD) dosing (Table 1). IM156 was administered in the fasted state, with no food for at least 2 h before and 1 h after dosing. As administration of radiographic iodinated contrast media may cause acute renal failure leading to the accumulation of IM156 and a potential risk of lactic acidosis, administration of IM156 was interrupted at least 48 h prior to each computed tomography (CT) scan and resumed after confirmation of the serum creatinine level within the normal range (or baseline) 48 h after the CT scan.

**Table 1:** Dose-levels and dose-limiting toxicities of oral IM156

**Dose level**	**IM156 dose**	**IM156 schedule**	**Number of patients**	**Dose-limiting toxicity**
1	100 mg	QOD	3	0
2	200 mg	QOD	3	0
3	400 mg	QOD	3	0
4	800 mg	QOD	3	0
5	1,200 mg	QOD	3	0
6	800 mg	QD	3	0
7	1,200 mg	QD	4	0

*QOD* once every other day, *QD* once daily

The planned sample size for this dose-escalation study was based on the observed incidence of dose-limiting toxicities (DLTs) in cohorts of 3–6 patients per dose level utilizing a 3 + 3 design, which prohibited dose escalation if DLT frequencies exceeded 33%. Patients evaluable for DLT assessment must have received at least 70% (QOD schedule) or 75% (QD schedule) of the planned IM156 doses during cycle 1 (first 28 days), had sufficient safety evaluations, or experienced a DLT.

Three DLT-evaluable patients were enrolled at each dose level. If there were no DLTs or other prohibitive adverse events (AEs), the Safety Committee was allowed to recommend escalation to the next highest dose level. If 1 DLT occurred in the first 3 DLT-evaluable patients, enrollment in that cohort was expanded to an additional 3 DLT-evaluable patients; if ≥ 2 DLTs were reported in a cohort, that dose was considered above the MTD. If there were no further DLTs reported, the Safety Committee was allowed to recommend further dose escalation unless lower grade AEs that did not meet the protocol definition of DLTs were considered to be potentially dose-limiting.

### Patients

Patients with advanced solid tumors refractory to standard therapies were enrolled in this study; investigators were encouraged to enroll patients with tumors predicted to be dependent on OXPHOS based on preclinical studies, such as gastric cancer, colorectal cancer, breast cancer, and glioblastoma multiforme. Patients were eligible for study enrollment if they were > 18 years of age, had a life expectancy of more than 12 weeks, Eastern Cooperative Oncology Group (ECOG) performance status ≤ 2, adequate organ function and measurable or evaluable lesions according to Response Evaluation Criteria in Solid Tumors (RECIST) v1.1 or Response Assessment in Neuro-Oncology (RANO) for tumors of the central nervous system [15, 16]. Patients with any of the following were excluded: major surgery < 4 weeks prior to treatment with IM156; radiotherapy < 3 weeks prior to treatment with IM156; had not discontinued all previous systemic cancer therapies at least 3 weeks for chemotherapy (6 weeks for nitrosourea compounds), or > 5 half-lives or 3 weeks (whichever is shorter) for biologic agents prior to treatment; had not fully recovered from acute toxicities of therapy. Additional detailed informations can be found in the study protocol (Supplementary File 1).

### Assessments

Safety was assessed through vital signs, weight, laboratory assessments, physical examinations, electrocardiogram, and reporting of AEs as graded by the National Cancer Institute’s Common Terminology Criteria for Adverse Events v4.03 (NCI-CTCAE).

DLTs were defined as the following AEs if they were attributed as related to IM156: asymptomatic grade 4 neutropenia lasting for ≥ 5 days; febrile neutropenia, regardless of grade; grade 4 thrombocytopenia or grade 3 thrombocytopenia with bleeding; any grade ≥ 3 non-hematologic AE (except grade 3 hypertension and grade 3 nausea, vomiting or diarrhea for which proper measures for prevention and treatment were not implemented, and alopecia); blood lactate levels > 5 mmol; grade ≥ 2 AE prohibiting dosing of IM156 for ≥ 21 continuous days except for alterations in serum calcium or phosphorus levels. The safety analysis includes all patients who received at least one dose of study drug.

Blood samples for PK assessments were obtained on Cycle 1 Day 1 (C1D1) and Cycle 1 Day 27 (C1D27; QOD schedule) or Cycle 2 Day 1 (C2D1; QD schedule) immediately prior to dosing and at 0.5, 1, 2, 4, 8, 12, and 24 h after dose administration; the 24-h sample was obtained just prior to administration of the next dose of IM156. PK samples were also obtained on Cycle 1 Day 15 immediately prior to dosing and 2 h after dosing. Urine samples for PK analysis were obtained immediately prior to dosing and continuously for pooled samples from 0–4, 4–12, 12–24 h after dosing on C1D1 and C1D27/C2D1.

Blood samples for assessment of exploratory PD markers in peripheral blood mononuclear cells (PBMCs) were to be obtained at 0, 2, and 4 h on C1D1 and at 0 and 2 h on C1D15.

A preliminary evaluation of efficacy was based on serial CT or magnetic resonance imaging (MRI). Response criteria were as defined by RECIST 1.1 or RANO. The following endpoints were to be assessed: overall response rate (ORR: complete response [CR] plus partial response [PR]), disease control rate (DCR: CR + PR + stable disease [SD]), DoR, and PFS.

### Statistical methods

The safety population was defined as all patients who received at least one dose of IM156. The PK population was not defined in the protocol but included all patients with sufficient PK data to perform adequate PK modeling. The efficacy population included all patients who underwent at least one tumor imaging assessment while receiving IM156. Safety was assessed using descriptive statistics (e.g., DLTs, AEs, laboratory tests, physical examination, 12-lead ECG). The incidence and number of events were reported for the DLTs observed in Cycle 1 per dose group. For treatment emergent adverse events (TEAEs; AEs that did not exist prior to but occurred after, or were exacerbated by, IM156 administration), the numbers and incidences of TEAEs and treatment-related adverse events (TRAEs) were reported by dose group. Two-sided 95% Confidence Intervals (CIs) were also provided. Adverse events and TRAEs were standardized with the System Organ Class and the Preferred Term using the Medical Dictionary for Regulatory Activities v23.0. In addition, TEAEs, serious adverse events (SAEs), TEAE severity, and TEAEs leading to withdrawal were presented by dose group. Efficacy data were to be presented by Waterfall and Swim Lane plots. Time to event endpoints (e.g., DoR, PFS) were determined using Kaplan–Meier estimations with the median value for each endpoint summarized.

## Results

### Baseline characteristics

Between October 2017 and July 2020, a total of 22 patients were enrolled in the study. All patients were Asian/Korean, 12 (55%) were female, and their ages ranged from 25 to 81 years of age (median: 57). Demographics of these patients can be found in Table 2. The most common malignancies were gastric cancer (*n* = 7, 32%), colorectal cancer (*n* = 3, 14%), ovarian cancer (*n* = 3, 14%), endometrial cancer (*n* = 2, 9%) and soft tissue sarcoma (*n* = 2, 9%).

**Table 2:** Patients’ characteristics

Characteristic	Number	Percentage (%)
**All**	22	100
**Gender**		
Male	10	45
Female	12	55
**Ethnicity**		
Asian	22	100
**Median age**, years (range)	57 (25–81)	
**Cancer type**		
Gastric cancer	7	32
Colorectal cancer	3	14
Ovarian cancer	3	14
Endometrial cancer	2	9
Soft tissue sarcoma	2	9
Other cancers (breast cancer, gastric neuroendocrine carcinoma, glioblastoma, prostate cancer, renal cancer)	5	23
**Median number or prior therapies (range)**	4 (1–11)	
**ECOG PS**		
0	14	64
1	8	36

*ECOG* Eastern Cooperative Oncology Group Performance Status

### Safety

In cohorts 1 through 5, IM156 was administered QOD at daily doses of 100 mg – 1,200 mg. In cohorts 6 and 7, IM156 was administered QD at daily doses of 800 mg and 1,200 mg, respectively (Table 1). The switch from QOD to QD dosing was based on the safety profile and PK data. There were no DLTs reported at any of the dose levels. TEAEs are summarized in (Supplementary Table 1); a total of 146 TEAEs were reported, with at least one TEAE reported in all 22 (100%) patients.

The median duration of IM156 treatment was 51 days (range 18–406). A total of 7 (32%) patients experienced a TEAE leading to dose interruption; the median number of days of interruption due to a TEAE was 3 (range 2–11). Only one patient required more than one dose interruption, a patient in the 800 mg QD cohort whose dose was initially interrupted for a Grade 1 toothache in Cycle 3 that did not recur after re-challenge at 800 mg QD. He subsequently had a Grade 1 asparate aminotransferase increase in Cycle 13 for which IM156 was interrupted and that did not recur after a second re-challenge at 800 mg QD. One patient in the 1,200 mg QD cohort required a dose reduction to 800 mg QD due to Grade 3 nausea and subsequently discontinued the study due to progressive disease.

TRAEs were reported in 19 (86%) patients, more specifically all patients except those administered IM156 in the 100 mg QOD group (Table 3). The most common TRAEs were gastrointestinal (GI), occurring in 18 (82%) patients. Nausea was reported in 15 (68%) patients, diarrhea in 10 (46%) patients, emesis in 9 (41%) patients, and abdominal pain and constipation in 2 (9%) patients, respectively. Nausea and emesis required anti-emetic medications in 14 (64%) patients, the most common class being 5-HT3 receptor antagonists (ramosetron or ondansetron for 11 [50%] patients. Other TRAEs of note included fatigue in 4 (18%) of patients and blood lactic acid increased in 2 (9%) patients. TRAEs grade 3 or higher were infrequent and included only 3 (14%) patients with grade 3 nausea at 1,200 mg QOD (*n* = 1) and 1,200 mg QD (*n* = 2) (Table 3).

**Table 3:** Treatment-related adverse events in at least 2 patients

**Adverse event**	**IM156 orally QOD**										**IM156 QD**				Total (N = 22)	
	**DL1** **100 mg** **(N = 3)**		**DL2** **200 mg** **(N = 3)**		**DL3** **400 mg** **(N = 3)**		**DL4** **800 mg** **(N = 3)**		**DL5** **1,200 mg** **(N = 3)**		**DL6** **800 mg** **(N = 3)**		**DL7** **1,200 mg** **(N = 4)**			
	**All**	** > G3**	**All**	** > G3**	**All**	** > G3**	**All**	** > G3**	**All**	** > G3**	**All**	** > G3**	**All**	** > G3**	**All**	** > G3**^a^
**Nausea**	**0**	**0**	**1**	**0**	**3**	**0**	**3**	**0**	**2**	**1**	**2**	**0**	**4**	**2**	**15**	**3**
**Diarrhoea**	**0**	**0**	**1**	**0**	**0**	**0**	**3**	**0**	**2**	**0**	**3**	**0**	**1**	**0**	**10**	**0**
**Vomiting**	**0**	**0**	**2**	**0**	**1**	**0**	**0**	**0**	**2**	**0**	**1**	**0**	**3**	**0**	**9**	**0**
**Fatigue**	**0**	**0**	**0**	**0**	**0**	**0**	**1**	**0**	**0**	**0**	**0**	**0**	**3**	**0**	**4**	**0**
**Abdominal Pain**	**0**	**0**	**0**	**0**	**0**	**0**	**1**	**0**	**0**	**0**	**0**	**0**	**1**	**0**	**2**	**0**
**Constipation**	**0**	**0**	**0**	**0**	**0**	**0**	**0**	**0**	**0**	**0**	**1**	**0**	**1**	**0**	**2**	**0**
**Blood Lactic Acid increased**	**0**	**0**	**0**	**0**	**0**	**0**	**0**	**0**	**0**	**0**	**1**	**0**	**1**	**0**	**2**	**0**
**Decreased appetite**	**0**	**0**	**1**	**0**	**0**	**0**	**0**	**0**	**1**	**0**	**0**	**0**	**1**	**0**	**2**	**0**
**Insomnia**	**0**	**0**	**1**	**0**	**1**	**0**	**0**	**0**	**0**	**0**	**0**	**0**	**0**	**0**	**2**	**0**

*QOD* once every other day, *QD* once every day, *DL* dose level, *G* grade

^a^There were no treatment related grade 4 adverse events

Of 28 laboratory AEs, most (26, 93%) were considered not related to IM156. Only Grade 1 increases in blood lactate in 2 (9%) patients were attributed as possibly related to IM156, one in the 800 mg QD cohort and the other in the 1,200 mg QD cohort in whom the elevated blood lactate decreased while the patient remained on study drug. There was no trend toward increased blood lactate levels with dose or exposure.

Nine SAEs were reported in 8 patients, only 1 of which (grade 3 nausea at 1,200 mg QOD that required a dose interruption) was attributed to IM156 (Supplementary Table 2).

Although no DLTs were reported, IM156 was not well tolerated at 1,200 mg QD. All 4 patients developed nausea attributed to IM156, 2 of which were Grade 3; 3 of these patients also developed IM156-related Grade 1 vomiting. Grade 3 nausea resolved to Grade 1 or 2 with anti-emetic medications and therefore did not meet criteria for a DLT; however, one of these patients had a dose reduction to 800 mg QD and the other a dose interruption. Although per protocol the MTD was not reached, the poor tolerability of IM156 at 1,200 mg QD, supported by PK data demonstrating exposures in the predicted efficacious range at 800 mg QD in preclinical animal models, led to 800 mg QD being declared the RP2D.

### Pharmacokinetics and pharmacodynamics

Following oral administration of IM156, mean T_max_ values appeared to be independent of dose and ranged from 2.3 h – 6.7 h on Day 1 and 1.2 – 4.0 h on Day 27. On Day 1, using data summarized combining both dosing regimens over the full dose range evaluated, AUC_0-24_ values increased in a dose proportional manner at doses ≥ 200 mg (Table 4). Data for the QOD regimens on Day 27 similarly showed exposure increased in a dose proportional manner when the 100 mg QOD dose cohort was excluded. Dose proportionality was difficult to assess following QD administration as only two dose cohorts of IM156 were administered with this dosing schedule; however, mean Day 27 AUC_0-24_ exposure values were higher following QD versus QOD administration of IM156 at the respective dose levels.

**Table 4:** Pharmacokinetics of IM156 on Days 1 and 27 of Cycle 1 in Plasma

**Day**	**Cohort**	**Dosed** **(mg)**	**N**	**T**_**max**_	**C**_**max**_	**AUC**_**0-24**_	**AUC**_**0-48**_	**AUC**_**INF**_	**Vz/F**	**CL/F**	**t**_**1/2**_
				**(h)**	**(ng/mL)**	(h*ng/mL)	(h*ng/mL)	(h*ng/mL)	**(L)**	**(L/h)**	**(h)**
1		100	3	6.67 ± 2.31	38.7 ± 31.1	482 ± 342					
		200	3	4.67 ± 3.06	189 ± 41.0	2980 ± 251					
		400	3	3.33 ± 1.15	362 ± 131	4380 ± 3010					
		800	6	2.25 ± 1.47	829 ± 260	7520 ± 3820					
		1200	7	5.00 ± 3.00	1100 ± 616	14,100 ± 6970					
Fed 1		1200	3	6.83 ± 5.84	1220 ± 910	17,600 ± 12,800					
27	1	100 QOD	2	2.50 ± 2.12	109 ± 54.8	1070 ± 330	1400 ± 383	1620 ± 473	1580 ± 291	64.5 ± 18.8	17.3 ± 1.91
	2	200 QOD	3	4.00 ± 0.00	272 ± 43.8	4200 ± 1140	6010 ± 1810	6180 ± 2220	837 ± 266	34.6 ± 12.4	16.9 ± 0.752
	3	400 QOD	3	4.00 ± 0.00	530 ± 324	7630 ± 5410	10,900 ± 8770	6470 ± 2700	1160 ± 256	67.8 ± 28.3	12.4 ± 2.56
	4	800 QOD	3	1.17 ± 0.764	949 ± 408	12,900 ± 9470	18,200 ± 14,700	11,200 ± 5740	1650 ± 413	82.4 ± 42.3	14.9 ± 4.20
	5	1200 QOD	3	3.33 ± 1.15	1580 ± 75.3	21,800 ± 3680	29,800 ± 4700	33,100 ± 6120	863 ± 312	36.9 ± 6.83	15.9 ± 2.90
	6	800 QD	3	4.00 ± 0.00	1870 ± 485	33,600 ± 9780					
	7	1200 QD	2	4.00 ± 0.00	1980 ± 94.8	36,300 ± 7550					

*mg* milligrams, *T*_*max*_ time at maximum concentration, *h* hours; *Cmax* maximum concentration, *ng* nanogram, *mL* milliliter, *AUC*_*0-24*_ area under the curve from time 0 to 24 hours, *AUC*_*0-48*_ area under the curve from time 0 to 48 hours, *AUC*_*0-inf*_ area under the curve from time 0 to infinity, *Vz/F* apparent volume of distribution, *CL/F* apparent total clearance, *t*_*1/2*_ terminal elimination half-life

**Fig. 1:**
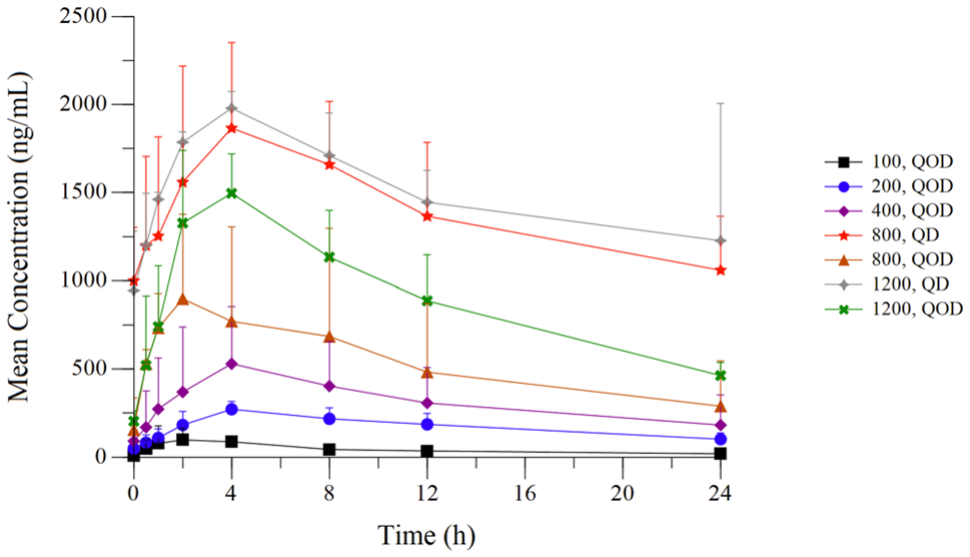
Steady-state (Day 27) plasma concentration profile of IM156

The mean plasma half-life (t_1/2_) values ranged from 12—17 h post dose administration (Fig. 1), and additional PK parameters are listed Table 4. Analysis of urine samples indicated that on Day 1, in the dose range of 200—1200 mg QOD, approximately 8–10% of IM156 is excreted in the urine (Supplementary Table 3). On day 27 the amount excreted, in the same dose range, increased to 16—18%. Although the number of patients is small, when QOD dosing is compared to QD, for a given dose, whether on day 1 or day 27, the percent of IM156 excretion did not change appreciably (Supplementary Table 3).

A circulating major metabolite of IM156 was identified in pooled human samples collected following oral QOD administration of 400 mg of IM156 using LC–MS/MS and UV detection methods. The structure of this metabolite was determined and confirmed by comparison to synthetic standard. This metabolite resulted from oxidation of the pyrrolidine ring to the corresponding carboxylic acid derivative. The metabolite was found to have no effect on cell viability or oxygen consumption rate up to concentrations of at least 50 μM in A549 human lung cancer cells (Supplementary Fig. 1).

The maximum plasma exposure achieved in this study was ~ 6 μM in patients who received IM156 at daily doses of 800 mg. This concentration of IM156 provided very little inhibition of OXPHOS when tested in whole blood ex vivo, consistent with in vitro cell assays and protein binding measurements. As such, measurements of target inhibition were not informative using PBMCs-based assays.

### Efficacy

**Fig. 2:**
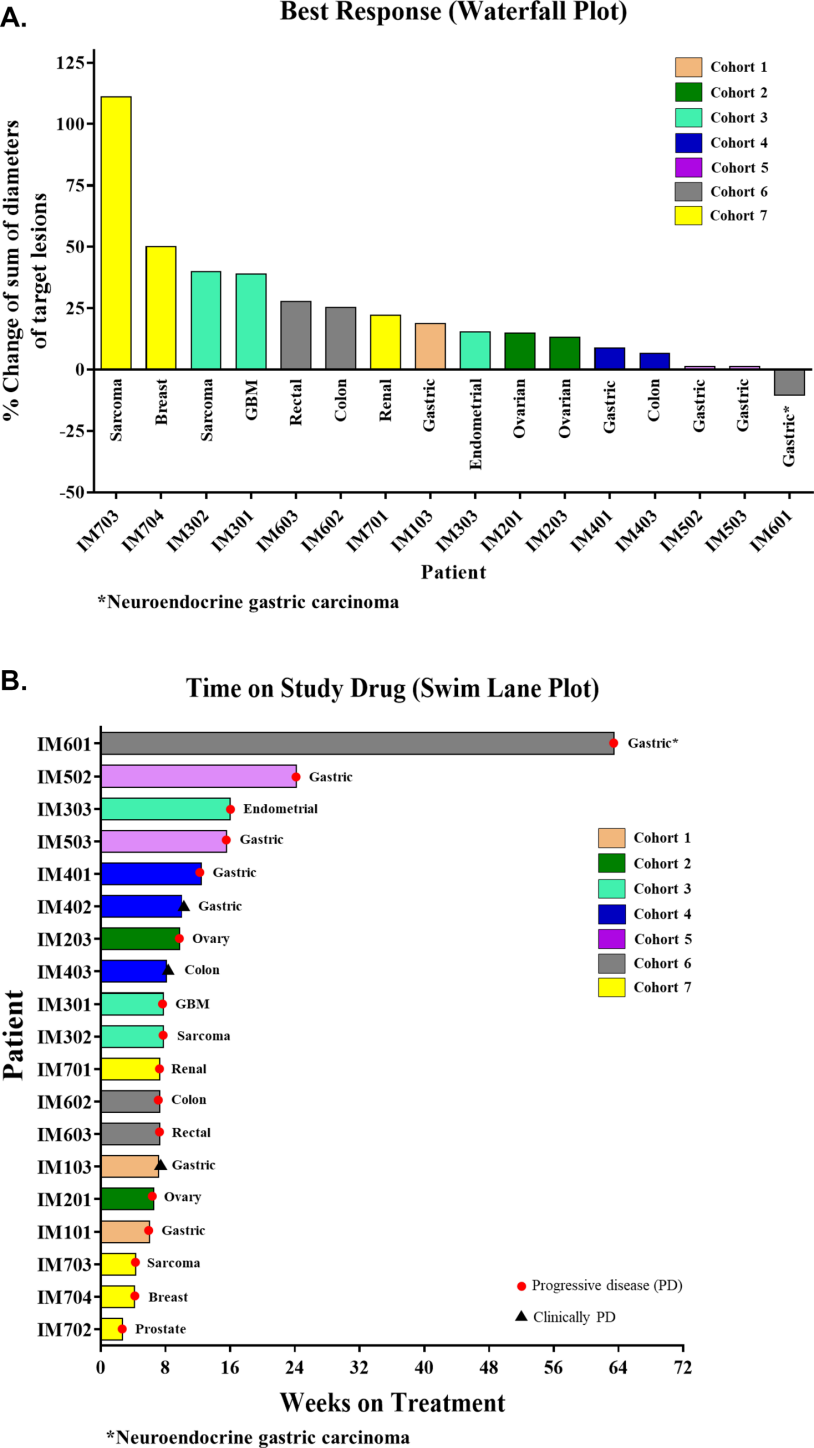
**A.** Waterfall plot demonstrating best response. Response data was available for 16 of the 22 patients enrolled. Of the 6 patients in whom response assessment was not possible, 2 withdrew consent prior to the first imaging timepoint, 3 did not have evaluable disease, and 1 was a protocol violation (received cytotoxic chemotherapy within 21 days of C1D1). **B.** Swim Lane Plot indicating duration of therapy in individual patients. Data are available for 19 of the 22 patients enrolled. Of the 3 patients in whom assessment was not possible, 2 withdrew consent prior to the first imaging timepoint and 1 was a protocol violation (received cytotoxic chemotherapy within 21 days of C1D1). Abbreviations: GBM, glioblastoma

Response data was available for 16 of the 22 patients. Of the 6 patients in whom response assessment was not possible, 2 withdrew consent prior to the first imaging timepoint, 3 did not have evaluable disease, and 1 was a protocol violation (received cytotoxic chemotherapy within 21 days of the first dose of IM156). No objective responses (CRs or PRs) were observed while SD was observed in 7 patients resulting in a DCR of 32%. One (1) patient with gastric neuroendocrine carcinoma and 1 with gastric adenocarcinoma, had a best response of prolonged SD for 444 days and 169 days, respectively (Fig. 2). The median PFS was 54 days (95% CI 47–71 days, Supplementary Fig. 2). Because the efficacy was perceived insufficient for further monotherapy development in an unselected population, initially planned expansion cohorts at the RP2D was not opened.

## Discussion

To our knowledge, this first-in-human study of IM156 is the first successfully completed phase 1 study of a PC1 inhibitor in patients with cancer with a RP2D determined for further clinical development. Other PC1 inhibitors, such as BAY 87–2243 [17], ASP4132 [18], and IACS-010759 [19], have all discontinued clinical development for oncology indications due to poor tolerability, primarily fatigue and AEs of the GI and nervous systems. By contrast, IM156 appears to have a favorable safety and tolerability profile, with treatment-related Grade ≥ 3 AEs occurring infrequently (14% of patients). The toxicity profile of IM156 is primarily gastrointestinal AEs, including nausea and vomiting in 68% and 41% of patients, respectively. Despite these AEs being primarily low-grade, their frequency, persistence, and limited resolution with anti-emetic drugs such as 5-hydroxytryptamine (5HT3) receptor antagonists ultimately precluded further dose escalation. Though no DLTs were reported, the RP2D of IM156 for patients with cancer is 800 mg orally daily, a dose that resulted in exposure above the therapeutic target predicted from preclinical cancer models.

Gastrointestinal AEs have also been observed with other biguanides and drugs targeting PC1 [9, 19, 20]. For instance, in a Phase 1 study of 21 patients of a selective non-biguanide inhibitor of PC1, IACS-010759, nausea and vomiting of any grade were reported in 67% and 29% of patients, respectively. Although diarrhea of any grade was more common in patients treated with IM156 versus IACS-010759 (50% vs. 10%), Grade ≥ 3 AEs were more frequent with IACS-010759 compared to IM156 (43% vs 14%). Additionally, IACS-010759 led to debilitating AEs not observed with IM156, such as Grade ≥ 3 visual impairment and peripheral neuropathy, and elevations of blood lactate (71% vs. 9%). The latter may be a result of a differences in the drugs’ binding to PC1 and degree of target inhibition, with a steeper dose–effect relationship of IACS-10759 than for IM156.

Two patients, 1 each at 800 and 1,200 mg QD, had Grade 1 asymptomatic elevation in blood lactate. Increased blood lactate is potentially an on-target effect of IM156; one could hypothesize that inhibition of mitochondrial OXPHOS would increase the rate of glycolysis with a subsequent rise in lactate production; however, lactate elevation is nonspecific.

Pharmacokinetic data demonstrated dose proportionality for both C_max_ and exposure at doses ≥ 200 mg. IM156 exposures following QD dosing were predicted from modeling of the data obtained with QOD dosing, supporting the switch to QD administration. Plasma exposures of IM156 at 800 mg QD demonstrated modest accumulation at steady-state (Day 1 C_max_ 829 ± 260 ng/mL, AUC_0-24_ 7,520 ± 3,820 h*ng/mL; Day 27 C_max_ 1,870 ± 485 ng/mL; AUC_0-24_ 33,600 ± 9,780 h*ng/mL) (Table 4). These values are in the range of those expected to demonstrate response based on robust efficacy in preclinical in vivo mouse models dosed at 15 – 30 mg/kg (Immunomet, Data on file); the AUC_0-24_ in single-dose oral PK studies in mice administered a 30 mg/kg dose was approximately 3.0 – 3.5 h*ng/mL. Our recent publication reported that IM156 is highly distributed to major metabolic organs such as lung, liver, kidney, etc. and that tissue concentrations of IM156 are 30–80 fold higher compared to plasma [21]. As such, measurements of the concentrations and activities of IM156 in plasma are not informative and significantly underestimate target tissue exposures and associated activity. In short, measurement of circulating plasma IM156 levels is a poor surrogate for target tissue exposures. A recently completed Phase 1 study in healthy volunteers using appropriate tissue-based biomarkers was designed to further explore and confirm the PK and target engagement of IM156 (Immunomet, Data on file).

IM156 metabolic profiling was completed for human samples following oral QOD administration of 400 mg of IM156 to cancer patients. One major metabolite detected in the pooled human plasma samples. Similarly, this metabolite was identified as a major circulating metabolite in rat and dog following oral administration of a single- or multiple-dose of IM156 in preclinical toxicity studies. This metabolite was negative in an in silico computational prediction of genetic toxicity, and no inhibition were greater compared to IM156 against off-target inhibition safety pharmacology profiling panel. Finally, this metabolite did not inhibit OXPHOS with no effect on cell viability up to concentration of 50 μM. These data demonstrate that this metabolite showed limited biological activity in mechanism of action and safety profile.

No clinical responses were observed but 32% of patients had SD as the best response, including 1 with gastric neuroendocrine carcinoma and 1 patient with gastric adenocarcinoma who remained on IM156 for 444 and 169 days, respectively. In an unselected population of patients whose cancer has not demonstrated dependence on OXPHOS, this level of anticancer activity is not supportive for further development as a monotherapy. However, IM156’s favorable safety profile suggests the potential for further development in rationally designed combinations with other anticancer agents, and as monotherapy for selected patients whose tumors have molecular profiles demonstrate a therapeutic vulnerability to OXPHOS inhibition [22].

Similar to IM156, the efficacy results of IACS-010759 showed one (5%) PR in a patient with prostate cancer and 8 (38%) patients with SD [19]. A switch to glycolysis has been speculated as a plausible mechanism for insufficient single-agent activity of other small molecule inhibitors of PC1. An adaptive switch to glycolysis in a chronic lymphocytic leukemia mouse model treated with IACS-010759 suggested that simultaneous use of a glycolysis inhibitor may be required to demonstrate efficacy [23], a combination that, due to the essential nature of the OXPHOS, may plausibly lead to significant toxicity.

In summary, our study demonstrated that IM156 is tolerable at RP2D of 800 mg QD. These data suggest that clinical development of PC1 inhibitors may be feasible. However, IM156 demonstrated rather limited single agent clinical activity in the unselected population, and we did not identify any biomarker associated with therapeutic response. Therefore, the study did not advance into monotherapy expansion cohorts at the RP2D. Further development in oncology will focus on rational combinations with other anticancer agents such as targeted therapies or chemotherapy and on continuing efforts to identify molecular markers predicting anticancer activity of IM156.

## Supplementary Information

Below is the link to the electronic supplementary material.

Supplementary file1 (PDF 1958 KB)

Supplementary file1 (DOCX 780 KB)

## Data Availability

Clinical study protocol is included in the submission.

## References

[1] Weinberg SE, Chandel NS (2015). Targeting mitochondria metabolism for cancer therapy. Nat Chem Biol.

[2] Mimaki M, Wang X, McKenzie M (2012). Understanding mitochondrial complex I assembly in health and disease. Biochim Biophys Acta.

[3] Izreig S, Gariepy A, Kaymak I (2020). Repression of LKB1 by miR-17 approximately 92 Sensitizes MYC-Dependent Lymphoma to Biguanide Treatment. Cell Rep Med.

[4] Birsoy K, Possemato R, Lorbeer FK (2014). Metabolic determinants of cancer cell sensitivity to glucose limitation and biguanides. Nature.

[5] Gravel SP, Hulea L, Toban N (2014). Serine deprivation enhances antineoplastic activity of biguanides. Cancer Res.

[6] Pollak M (2014). Overcoming Drug Development Bottlenecks With Repurposing: Repurposing biguanides to target energy metabolism for cancer treatment. Nat Med.

[7] Viale A, Pettazzoni P, Lyssiotis CA (2014). Oncogene ablation-resistant pancreatic cancer cells depend on mitochondrial function. Nature.

[8] Ming M, Sinnett-Smith J, Wang J et al (2014) Dose-Dependent AMPK-Dependent and Independent Mechanisms of Berberine and Metformin Inhibition of mTORC1, ERK, DNA Synthesis and Proliferation in Pancreatic Cancer Cells. PLoS One 9(12) 10.1371/journal.pone.0114573 PMC4262417 25493642

[9] Kordes S, Pollak MN, Zwinderman AH (2015). Metformin in patients with advanced pancreatic cancer: a double-blind, randomised, placebo-controlled phase 2 trial. Lancet Oncol.

[10] Arrieta O, Barron F, Padilla MS et al (2019) Effect of Metformin Plus Tyrosine Kinase Inhibitors Compared With Tyrosine Kinase Inhibitors Alone in Patients With Epidermal Growth Factor Receptor-Mutated Lung Adenocarcinoma: A Phase 2 Randomized Clinical Trial. JAMA Oncol e192553 10.1001/jamaoncol.2019.2553 PMC6735425 31486833

[11] Algire C, Amrein L, Bazile M (2011). Diet and tumor LKB1 expression interact to determine sensitivity to anti-neoplastic effects of metformin in vivo. Oncogene.

[12] Wheaton WW, Weinberg SE, Hamanaka RB (2014). Metformin inhibits mitochondrial complex I of cancer cells to reduce tumorigenesis. Elife.

[13] Vara-Ciruelos D, Dandapani M, Russell FM et al (2019) Phenformin, But Not Metformin, Delays Development of T Cell Acute Lymphoblastic Leukemia/Lymphoma via Cell-Autonomous AMPK Activation. Cell Rep 27 (3): 690–698 e694 10.1016/j.celrep.2019.03.067 PMC6484776 30995468

[14] Choi J, Lee JH, Koh I (2016). Inhibiting stemness and invasive properties of glioblastoma tumorsphere by combined treatment with temozolomide and a newly designed biguanide (HL156A). Oncotarget.

[15] Eisenhauer EA, Therasse P, Bogaerts J et al (2009) New response evaluation criteria in solid tumours: revised RECIST guideline (version 1.1). Eur J Cancer 45 (2):228–247 10.1016/j.ejca.2008.10.026 19097774

[16] Wen PY, Chang SM, Van den Bent MJ (2017). Response Assessment in Neuro-Oncology Clinical Trials. J Clin Oncol.

[17] Ellinghaus P, Heisler I, Unterschemmann K (2013). BAY 87–2243, a highly potent and selective inhibitor of hypoxia-induced gene activation has antitumor activities by inhibition of mitochondrial complex I. Cancer Med.

[18] Janku F, LoRusso P, Mansfield AS et al (2021) First-in-human evaluation of the novel mitochondrial complex I inhibitor ASP4132 for treatment of cancer. Invest New Drugs 2021 10.1007/s10637-021-01112-7 33830407

[19] Yap TA, Ahnert JR, Piha-Paul SA et al (2019) Phase I trial of IACS-010759 (IACS), a potent, selective inhibitor of complex I of the mitochondrial electron transport chain, in patients (pts) with advanced solid tumors. J Clin Oncol 37 (15_suppl):3014–3014

[20] Schwartz S, Fonseca V, Berner B (2006). Efficacy, tolerability, and safety of a novel once-daily extended-release metformin in patients with type 2 diabetes. Diabetes Care.

[21] Willette RN, Mangrolia P, Pondell SM (2021). Modulation of Oxidative Phosphorylation with IM156 Attenuates Mitochondrial Metabolic Reprogramming and Inhibits Pulmonary Fibrosis. J Pharmacol Exp Ther.

[22] Garofano L, Migliozzi S, Oh YT (2021). Pathway-based classification of glioblastoma uncovers a mitochondrial subtype with therapeutic vulnerabilities. Nat Cancer.

[23] Vangapandu HV, Alston B, Morse J (2018). Biological and metabolic effects of IACS-010759, an OxPhos inhibitor, on chronic lymphocytic leukemia cells. Oncotarget.

