# Supplementary material for: First-in-human study of IM156, a novel potent biguanide oxidative phosphorylation (OXPHOS) inhibitor, in patients with advanced solid tumors
Source: Invest New Drugs. 2022 Jul 8;40(5):1001–10. doi: 10.1007/s10637-022-01277-9 (PMC9395488; doi:10.1007/s10637-022-01277-9)
Supplement: Supplementary file 1 — Supplementary file1 (PDF 1958 KB) [file 10637_2022_1277_MOESM1_ESM.pdf]

CLINICAL STUDY PROTOCOL

A Multi Center, Open-label, Phase 1 clinical trial to evaluate the safety, tolerability, and preliminary efficacy of IM156 in patients with advanced solid tumors and lymphoma

**Investigational Product**	IM156
**Protocol No.**	IM_IM156-01
**Protocol Version**	6.0
**Phase of Study**	Phase 1 trial
**Date of Preparation**	2020-03-24

CONFIDENTIAL

Since all information related to this protocol is confidential and the exclusive property of IMMUNOMET, no information can be released without the prior written consent of IMMUNOMET.

Revision History of clinical study protocol

Version	Date	Comment
1.0	2017-03-29	· Initial submission to the Ministry of Food and Drug Safety (MFDS)
2.0	2017-05-19	· Revision due to reflection of the complementary measures after review of the MFDS
2.1	2017-07-28	· Correction of a typo in the appearance of IP, revision and phrase correction to the shelf life of IP
2.2	2017-09-11	· Re-revision to the shelf life of IP and addition of prohibited concomitant medications<br>· Addition of requirements that IP should be interrupted prior to the contrast agent for the CT scan and renal function test should be performed after the CT scan before resume IP administrtaion
2.3	2017-12-21	· Added an optional sestamibi scan test for an exploratory objective and revision of terminology<br>· Added window period for blood tests on Cycle 2 and subsequent cycles.
3.0	2018-07-03	· Added text allowing an intermediate dose cohort<br>· Added fasting rule on IP administration<br>· Specification of PET scan according to disease characteristics<br>· Added the option to use an external company for additional analyses<br>· Modified the fourth exclusion criteria
3.1	2018-08-06	· Modified phrase according to the review by the MFDS
3.2	2018-11-22	· Modified phrase according to the addition of new study sites
4.0	2018-12-21	· Changed dosing method after the completion of Cohort 5 (QOD → QD)<br>· Changed next dose level after the completion of Cohort 5<br>· Changed the time point of molecular imaging exploratory markers<br>✓ Configured PET scan using ^{18}F-FDG probe as mandatory, but Sestamibi scan and PET scan using ^{11}C-acetate probe as optional<br>✓ Configured the scan(s) to be conducted with every 2-cycle interval after the baseline so that scans would be performed at the same time of tumor assessment<br>· Added description of lymphoma in the target indication and tumor assessment criteria for lymphoma

Version	Date	Comment
5.0	2019-05-23	· Added single dose stage for food effect study with a high fat meal at 7 days before Cycle 1<br>· Modified second exclusion criteria (to allow enrollment of patients with diabetes mellitus who are not taking a biguanide)<br>· Modified the definition of dose limiting toxicity and the dose adjustment rules to be consistent with daily versus every other day dosing<br>· Added comment to obtain archived tumor samples to assess for potential biomarkers<br>· Changed method for blood lactate analysis<br>· Modified the prohibited concomitant drugs and treatments<br>· Changed ^{18}FDG-PET scans to optional for recurrent glioblastoma patients<br>· Modified the background section<br>· Changed the study duration
6.0	2020-03-24	· Changed the visit frequency from every cycle to every other cycle from Cycle 13<br>· Modified the procedures at Cycle 13 and subsequently<br>· Added the list of exploratory markers

Synopsis

**Title**	A Multi Center, Open-label, Phase 1 clinical trial to evaluate the safety, tolerability, and preliminary efficacy of IM156 in patients with advanced solid tumors and lymphoma
**Phase and Design of Study**	Exploratory dose-finding pharmacology study (Phase 1)
**Number of Sites / Location**	Phase 1a: Up to three investigative sites in South Korea.<br>Phase 1b: Additional sites and countries may be added.
**Sponsor**	ImmunoMet Therapeutics
**Study Objectives**	1) Primary Objective<br>• To evaluate the safety and the tolerability of IM156 in patients with advanced solid tumors or lymphoma<br>• To determine the maximum tolerated dose (MTD) and/or recommended Phase 2 dose (RP2D) in patients with advanced solid tumors or lymphoma<br>2) Secondary Objectives<br>① To evaluate the pharmacokinetic (PK) characteristics of IM156<br>② To explore preliminary efficacy (tumor response) of IM156<br>• Objective response rate (ORR)<br>• Disease control rate (DCR)<br>• Duration of response (DoR)<br>• Progression-free survival (PFS)<br>3) Exploratory Objective<br>• Identification and profiling of IM156 metabolites<br>• To evaluate the exploratory markers of IM156<br>• To evaluate any potential relationship between putative biomarkers and safety/efficacy parameters of IM156
**Target indication**	Patients with solid tumors or lymphoma who have failed standard therapy
**Inclusion Criteria**	1) At least 19 years of age<br>2) Patients with histologically or cytologically confirmed advanced solid tumor or lymphoma<br>3) Patients for whom no standard therapies are available or who have failed in the existing conventional therapies<br>4) Patients with measurable or evaluable lesions according to Response Evaluation Criteria in Solid Tumors (RECIST) v1.1 (Response Assessment in Neuro-Oncology [RANO] for recurrent glioblastoma [rGBM] or RECIL (2017) for lymphoma)<br>5) Patients with Eastern Cooperative Oncology Group (ECOG) performance status 0, 1, or 2<br>6) Patients with adequate function of bone marrow, kidney and liver as follows<br>① Absolute neutrophil count (ANC) ≥ 1,500/mm^3 (≥ 1,000/mm^3 for lymphoma), Platelet ≥ 100,000/mm^3 (≥ 75,000/mm^3 for lymphoma), Hemoglobin ≥ 9.0 g/dL (In case of hemoglobin < 9.0 g/dL, the patient can be enrolled if the value is reversed to ≥ 9.0 g/dL. However, blood transfusion within 1 week of screening is not permitted)

	② Serum creatinine ≤ 1.5 × upper limit of normal (ULN)<br>③ Total bilirubin ≤ 1.5 × ULN, aspartate aminotransferase (AST) and alanine aminotransferase (ALT) ≤ 3 × ULN (for patients with liver disease ≤ 5 × ULN)<br>④ Fasting serum glucose ≤ 160 mg/dL<br>7) Patients with life expectancy ≥ 12 weeks<br>8) Patients who have agreed to use acceptable methods for contraception during the study treatment period<br>(e.g., sterilization of the patient and/or his/her partner, use of intrauterine device by the patient or the partner, barrier contraception such as a diaphragm or condom)<br>9) Patients who have voluntarily signed an informed consent to participate in this clinical study
**Exclusion Criteria**	1) Patients with a history of hypersensitivity to the active ingredient or any component of the investigational product (IP) or biguanides<br>2) Patients with a current evidence of diabetes mellitus who are currently being treated with another biguanide (e.g., metformin)<br>3) Patients with a history of serious gastrointestinal bleeding within 6 weeks prior to screening or patients with any disease possibly affecting the absorption of oral agents (malabsorption syndrome, hemorrhagic gastric ulcer, etc.)<br>4) At the time of scheduled starting date of initial IP administration,<br>• At least 4 weeks have not elapsed since any major surgery.<br>• At least 3 weeks have not elapsed since the last treatment with radiotherapy.<br>• At least 3 weeks have not elapsed since the last treatment with chemotherapy (at least 6 weeks for nitrosurea compounds).<br>• At least 5 half-lives or 3 weeks, whichever is shorter, have not elapsed since the last treatment with biologic agents including hormone therapy.<br>5) Patients whose toxicity due to prior therapy have not recovered to baseline or Grade 1 according to the NCI CTCAE v4.03 prior to screening<br>6) Pregnant or nursing women<br>7) Patients who were administered another IP within 3 weeks prior to screening<br>8) Patients with uncontrolled metastasis to the central nervous system. However, patients with treated and stable brain metastases (stable at least for 30 days on radiology imaging) are allowed to enroll<br>9) Patients with suspected serious infectious diseases, intestinal paralysis, bowel obstruction, interstitial pneumonia, or pulmonary fibrosis<br>10) Patients with a history of psychiatric disorders likely to threaten the compliance with this protocol<br>11) Patients with a history of alcohol or drug abuse within 12 weeks prior to screening<br>12) Human immunodeficiency virus (HIV) infection or active hepatitis B or C. Patients with no detectable viral load may be enrolled<br>13) Patients with severe traumatism

<table>
<tr><td></td><td>14) Patients with any clinically significant abnormal intestinal findings that may interfere with the administration, passage, or absorption of the IP, or which makes the patients unable to orally take the tablet form of drugs
15) Patients with severe cardiac disorders (e.g., myocardial infarction, congestive heart failure, arrhythmia showing dramatic change in electrocardiogram [ECG], severe or unstable angina, other serious cardiac disorders) or patients with comorbidities of other serious internal disorders (e.g., uncontrolled diabetes mellitus, chronic obstructive pulmonary disorder, renal failure, etc.) based on investigator’s judgment
16) Patients who are otherwise considered to be ineligible for this study based on investigator’s judgment</td></tr>
<tr><td>Target Number of Patients</td><td>Phase 1a: approximately 3 to 6 patients are planned to be enrolled by dose level in approximately 8 cohorts. Up to 36 patients are expected to be enrolled.
Phase 1b: approximately 30 patients</td></tr>
<tr><td>Study Duration</td><td>Entire study duration: Approximately 36 months from the approval date of the Institutional Review Board (IRB)
(Changeable depending on the rate of patient enrollment)</td></tr>
<tr><td>Investigational Products</td><td><u>IM156</u>
① Dosage form and appearance:
- IM156 100mg, 200mg: A white round tablet
- IM156 400mg: A white oblong tablet
② Active ingredient: IM156 100 mg, 200 mg, 400 mg</td></tr>
<tr><td>Dosage and Administration</td><td><u>Phase 1a</u>
The initial dose level (dose level 1) will be IM156 100 mg every other day (QOD). One cycle is defined as 4 weeks (28 days), and evaluation for dose limiting toxicity (DLT) will be carried out after one cycle of administration is completed. IM156 is planned to be administered at approximately8 dose levels, and dose escalation will proceed unless a DLT occurs. Dose escalation will be determined by the Safety Committee after each patient in a cohort has completed one cycle of IM156. Dose escalation will be continued until the MTD is determined.

[Note: After the completion of Cohort 5, the dosing schedule was changed to once daily dosing (QD)]
① Initial dose: 100 mg
② Dose escalation
<table>
<tr><th>Level</th><th>Dosage</th><th>No. of patients</th></tr>
<tr><td>1</td><td>100 mg QOD</td><td>3~6</td></tr>
<tr><td>2</td><td>200 mg QOD</td><td>3~6</td></tr>
<tr><td>3</td><td>400 mg QOD</td><td>3~6</td></tr>
<tr><td>4</td><td>800 mg QOD</td><td>3~6</td></tr>
<tr><td>5</td><td>1200 mg QOD</td><td>3~6</td></tr>
<tr><td>6</td><td>800 mg QD</td><td>3~6</td></tr>
<tr><td>7+</td><td>To be determined by Safety Committee</td><td>3~6</td></tr>
</table></td></tr>
</table>

* Dose escalation to the next cohort is based on assessment of DLTs after the last patient in each cohort has completed one cycle and received the required number of doses of IM156. Dose escalation to the next level within the same dose group will not be allowed.

③ Method and schedule for administration:
IM156 will be administered in a fasted state (at least 2 hours after and 1 hour before eating and drinking except water) except at the single dose stage (described below).
In the single dose stage, IM156 will be administered with a high-fat meal as described by (NITR, 2005). Following an overnight fast of at least 10 hours, subjects should eat a high-fat meal in 20 minutes starting 30 minutes prior to administration of IM156 with 240 mL of water. No food will be eaten for at least 4 hours after the administration of IM156. Water is allowed as desired except for one hour before and after the administration of IM156.

<u>Phase 1b:</u> advanced solid tumor or lymphoma
IM156 will be administered at the RP2D and schedule determined during Phase 1a. The population(s) and dose and schedule of IM156 will be defined in a protocol amendment.

Methodology

This study is a Phase 1 clinical study to determine the MTD and RP2D of IM156 as a single agent and to explore its safety, tolerability, PK characteristics, exploratory markers, and preliminary efficacy.
The Phase 1 clinical study consists of two parts, Phase 1a and Phase 1b. The Phase 1a is conducted to determine the MTD of IM156 as a single agent. In addition, the safety, tolerability, preliminary efficacy, PK characteristics, and exploratory markers of IM156 are evaluated in patients with advanced solid tumors or lymphoma who have failed in the standard therapies. Approximately 8 cohorts are planned in Phase 1a; the number of cohorts and the dose and schedule of IM156 to be administered at each dose levels will be determined by Safety Committee. The Phase 1b is conducted to confirm the safety and tolerability, to explore the preliminary efficacy, and to evaluate the exploratory markers at the RP2D of IM156 in patients with advanced solid tumor or lymphoma.

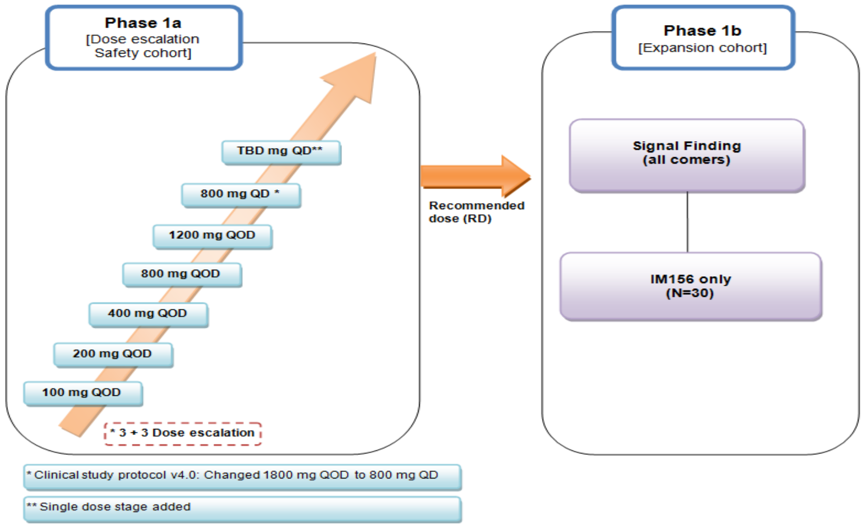

Figure 1. Study Scheme

	**Phase 1a**<br>The dose will be escalated sequentially from the lowest dose cohort (100 mg, QOD). Decisions to dose escalate will be made based on evaluation of all pertinent data obtained, including the occurrence of DLTs during the first cycle of treatment in each cohort and relevant PK data.<br>The RP2D for IM156 will be either the MTD [Refer to “Definition of dose limiting toxicity, and definition and determination of maximum tolerated dose” below] or a lower dose should it be supported by the totality of the data collected and analyzed. All patients will continue to receive IM156 until unacceptable toxicity occurs, progressive disease is confirmed according to RECIST guideline v1.1 (RANO for rGBM, RECIL (2017) for lymphoma), or any other reason to discontinue administration.<br><br>**Phase 1b**<br>IM156 will be administered at the RP2D and schedule determined during Phase 1a in 28 days cycle. Enrolled patients will continue to receive IM156 until there is an unacceptable toxicity, confirmation of progression disease according to RECIST v1.1 [Rano for rGBM, RECIL (2017) for lymphoma), or any other reason to discontinue administration.
**Definition of Dose Limiting Toxicity, and Definition and Determination of Maximum Tolerated Dose**	**1. Definition of dose-limiting toxicity (DLT)**<br>A DLT is defined as an adverse event (AE) or abnormal clinical laboratory value limiting gradual dose escalation applicable to any of the following criteria and that is not associated with the progressive disease or intercurrent disorders. To determine the dose escalation, DLT evaluation will be carried out after the first cycle only. Unless otherwise specified, toxicity will be evaluated by using the NCI-CTCAE v4.03. DLTs must be considered as related to IM156 administration.<br>① Hematologic toxicity<br>- Grade 4 ANC ≥ 5 days<br>- Febrile neutropenia as defined by CTCAE v4.03, regardless of the grade<br>- Grade 4 thrombocytopenia regardless of duration<br>- Grade 3 or 4 thrombocytopenia with bleeding<br>② Non-hematologic toxicity<br>- Toxicities of Grade 3 or greater (However, alopecia, and nausea, vomiting, and diarrhea of which proper measures for prevention and treatment have not been implemented are excluded)<br>- Note: Hypertension ≤ Grade 3 adequately controlled by an anti-hypertensive is not considered a DLT.<br>- Lactate levels > 5 mmol/L<br>③ Other<br>- Administration of at least 70% of the planned dose in QOD dose group or 75% of the planned dose in the QD dose group is infeasible during the administration period due to the IP-related toxicities<br>- Any ≥ Grade 2 significant IP-related toxicity (excluding calcium and

	phosphorus) that continues for at least 21 days, according to the investigator's judgment.<br><br>**2. Definition of maximum tolerated dose (MTD)**<br>The MTD will be determined as the highest dose at which ≤ 1 of 6 patients developed a DLT (≤ 1/6).<br><br>**3. Determination of MTD**<br>After DLT evaluation of 3 patients enrolled at each dose level,<br>① Dose will be escalated to the next level if none of the 3 patients experienced a DLT (0/3):<br>② If one of 3 patients experiences a DLT (1/3):<br>3 additional patients will be enrolled at the same dose,<br>➢ If one of 6 patients administered that dose experiences a DLT (1/3 + 0/3; 1/6): The dose will be escalated to the next level.<br>➢ If at least two of 6 patients administered that dose experience a DLT (≥ 2/6): 3 additional patients will be enrolled after reducing the dose to one lower dose level.<br>③ If two of 3 patients experiences a DLT (≥ 2/3), 3 additional patients enrolled after reducing the dose to one lower dose level<br>If 6 patients have already been administered the dose at one lower dose level, no additional patients will be enrolled and the one lower level dose will be determined as the MTD. However, if two of 3 patients experience a DLT at dose level 1, the clinical study will be terminated.<br><br>**4. Determination of RP2D**<br>RP2D is either the MTD or a lower dose based on additional factors including but not limited to delayed toxicities that occur.
**Withdrawal Criteria**	1) Progressive disease (PD)<br>2) Dose omission exceeds 4 weeks due to IP-related toxicities<br>3) Patient's voluntary withdrawal of consent<br>4) Patients have received other treatments or therapies during the study period without approval of the responsible physician that may affect the study results<br>5) Patients do not comply with investigator's directions<br>6) Patients who did not meet the inclusion/exclusion criteria<br>7) Patients who cannot be continued in the clinical study due to unmanageable toxicities, including patients that withdraw their consent due to an adverse event, regardless of grade of severity<br>8) Patients who are considered to be unable to participate in the clinical study due to a change in safety conditions and ethical perspective in the investigator's judgment<br>9) Confirmation of pregnancy during the administration of the IP<br>10) Patients who are lost to follow up<br>11) Any other case in which investigators determine the study should be discontinued
**Prohibited Concomitant Medications and**	The drugs and treatments listed below are prohibited during the administration of the IP.<br>1) Anti-cancer therapies including anti-cancer drugs or biologicals other

<table>
<tr><td>Treatments</td><td>than the IP
2) Immunosuppressants and systemic corticosteroids
(However, stable doses of prednisone of ≤ 20 mg daily or the equivalent dose of other corticosteroids is allowable for the treatment of non-malignant conditions)
3) Surgical treatments for malignant tumor
4) Radiotherapy (however, radiotherapy for pain relief is allowed if not a target lesion)
5) Other IPs</td></tr>
<tr><td>Evaluation Methods</td><td>1) Efficacy evaluation: Tumor assessment
• ORR
Proportions of patients evaluated as CR or PR for the best overall response
• DCR
Proportions of patients evaluated as CR, PR, or stable disease (SD) for the best overall response
• DoR
Period from the time of response (CR or PR) to the time of PD
• PFS
Time from the first day of study drug administration to PD or death

2) Safety parameters
① AEs
Clinical and laboratory toxicities and signs are graded and evaluated according to the NCI CTCAE v4.03. The AEs that cannot be graded according to the NCI-CTCAE will be classified as Grade 1 for mild, Grade 2 for moderate, Grade 3 for severe, Grade 4 for life-threatening, and Grade 5 for death based upon their maximum intensity.
② Vital signs
③ 12-lead ECG
④ Laboratory tests
⑤ Physical examination
⑥ ECOG performance status</td></tr>
<tr><td>Pharmacokinetic parameters and Exploratory markers</td><td>1) PK evaluation
<u>Phase 1a</u>
PK evaluation will be performed in each dose level. Patients may be hospitalized in the ward where 24-hour monitoring is possible, and blood and urine will be collected before and after IM156 adminstration for PK evaluation. IM156 should be given to patients who are in a fasted state except the single dose stage for food effect study. The time points for collecting blood and urine, and the evaluation parameters are as follows.
① Time points for blood collection:
➢ Single dose stage for food effect study
• Day -7: 0h (immediately before IP administration), 0.5h, 1h, 2h, 4h, 8h, 12h, 24h, 48h, and 72h post-administration of IP
➢ Cycle 1
• Day 1: 0h (immediately before IP administration), 0.5h, 1h,</td></tr>
</table>

	2h, 4h, 8h, 12h, and 24h (Day 2 0h) post-administration of IP<br>• Day 15: 0h (immediately before IP administration) and 2h post-administration of IP<br>➢ Cycle 2<br>• Day 1: 0h (immediately before IP administration), and 0.5h, 1h, 2h, 4h, 8h, 12h, and 24h post-administration of IP<br>The actual time of administration and of each blood draw is to be recorded in the CRF.<br><br>② Time points for urine collection:<br>➢ Single dose stage for food effect (Day -7): 0h (before IP administration, spot urine), and 0-4h, 4-12h, and 12-24h (collection of all urine within the time period as possible) post-administration of IP<br>➢ Cycle 1 Day 1: 0h (before IP administration, spot urine), and 0-4h, 4-12h, and 12-24h (collection of all urine within the time period as possible) post-administration of IP<br>➢ Cycle 2 Day 1: 0h (before IP administration, spot urine), 0-4h, 4-12h, and 12-24h post-administration of IP<br>(collection of all urine within the time period as possible)<br>③ Evaluation parameters: Area under the concentration-time curve (AUC) over the time interval from 0 extrapolated to infinity (AUC_{inf}), AUC up to the last measurable concentration (AUC_{0-last}), maximum plasma drug concentration (C_{max}), minimum plasma drug concentration (C_{min}), time taken to reach the maximum concentration (T_{max}), terminal phase half life ($T_{1/2}$), apparent total clearance of the drug from plasma after oral administration (CL/F), accumulation ratio at steady state, cumulative amount of unchanged drug excreted into the urine (Ae), renal clearance (CLr), etc.<br>④ Profiling and identification of IM156 metabolites is an exploratory objective. Analytical tests in association with metabolism will be conducted according to a separate protocol and reported separately from the clinical study report.<br><br>**<u>Phase 1b</u>**<br>Pharmacokinetic evaluation will be performed in the first cycle. The patients will be hospitalized for a 24-hour monitoring period, and blood and urine will be collected before and after IM156 administration for PK evaluation. The time points for collecting blood and urine, and the evaluation parameters are the same as the Phase 1a study, and the time points for blood/urine collection may be adjusted based on the Phase 1a study findings.<br><br>**2) Parameters of exploratory markers**<br>Exploratory markers will be evaluated using peripheral blood and tumor biopsy specimens. Molecular imaging markers, target-related markers, safety markers, and mechanism surrogate markers will be included in the evaluation.

	① Molecular imaging markers<br>PET scans using ^{11}C-acetate (optional from Cohort 6 onward at the discretion of investigator) and ^{18}F-FDG probe, and/or Sestamibi scan (Sestamibi scan is optional at the discretion of investigator in the Phase 1a) will be used to evaluate the change in tumor metabolism.<br>*PET scan using ^{18}F-FDG probe is mandatory, but Sestamibi scan and PET scan using ^{11}C-acetate probe are optional. ^{18}F-FDG-PET scans are not required for patients with GBM.<br>② Target-related markers<br>We will identify/assess exploratory markers related to effects of IM156 in tumor tissue. If possible, archived tumor samples will be obtained to assess for potential biomarkers.<br>③ Safety markers<br>Blood lactate level will be assessed using a portable lactate monitor.<br>④ Mechanism surrogate markers<br>Multiple potential markers of IM156 effects will be investigated, including those with preliminary data (e.g., Vascular endothelial growth factor levels, phosphorylation status of ribosomal protein S6 and acetyl Co-A carboxylase) as well as, but not limited to, potential markers of metabolism-related analytes, mechanism-related proteins such as redox proteins, TCA cycle-, lipid oxidation-, oxidative phosphorylation-, and tRNA ligase-enzymes, etc. and their products.<br><br>Time points of tests for evaluation of exploratory markers are as follows.<br>✓ Blood collection for the mechanism surrogate markers: 0h (prior to IP administration), 2h, and 4h (only on Day 1) on Day 1 and Day 15 of the first cycle.<br>✓ Molecular imaging markers: Day 1 of the first cycle (prior to administration of the IP), the same day as tumor assessments are conducted [i.e. every 2 cycles], and EOT.<br>✓ Tumor biopsy (perform if possible): Will be collected prior to study initiation and after EOT. Archived tumor samples, ideally from the most recent biopsy, will be provided if possible.
**Statistical Analysis Methods**	**1) Efficacy Endpoints**<br>• ORR:<br>Proportions of patients evaluated as CR or PR for the best overall response will be presented as the frequency and the percentage of patients. Patients will be summarized by dose group for the Phase 1a study, and the two-sided 95% CI will also be provided for the Phase 1b.<br>• DCR:<br>Proportions of patients evaluated as CR, PR, or SD for the best overall response should be presented as the frequency and the percentage of patients. For the Phase 1a study, patients will be summarized by dose group and the two-sided 95% confidence interval (CI) will also be provided for the Phase 1b.<br>• Time to Event Endpoints – DoR, PFS:<br>Kaplan-Meier curve will be estimated and the median value for each variable (median PFS) will be summarized. Patients will be

	summarized in total and by dose group for the Phase 1a study, and the two-sided 95% CI will also be provided for the Phase 1b study.<br><br>**2) Safety Endpoints**<br>The safety assessment provides descriptive statistics on the DLTs, AEs, laboratory tests, physical examination, 12-lead ECG, etc. according to data format. For the Phase 1a study, the incidence and number of events will be reported for the DLTs observed in Cycle 1 per dose group. For treatment emergent adverse events (TEAEs, the AEs that did not exist prior to administration of the IP but occurred after administration, or AEs that were exacerbated after administration of the IP although they existed prior to administration of the IP), the numbers and incidences of AEs and adverse drug reactions will be reported by dose group in the Phase 1a study and for all subjects in the Phase 1b study. Two-sided 95% CIs will also be provided. AEs and adverse drug reactions will be standardized with the SOC (System Organ Class) and the PT (Preferred Term) by using Medical Dictionary for Regulatory Activities (MedDRA). In addition, serious adverse events (SAEs), severity, AEs leading to withdrawal, and TEAEs will be presented by dose group for Phase 1a. Besides, descriptive statistics on laboratory test values, 12-lead ECG, etc. will be presented by each visit.

Study Flow Chart

Evaluation Items	Screening	Single dose stage				Cycle 1 (=28 days)[1]				Cycle 2		Cycle ≥3	Cycle ≥13 (every other cycle)	EOT/DC	Safety F/U
	D-21 ~ D-8	D-7	D-6	D-5	D-4	D1	D8	D15	D22	D1	D15	D1	D1	-	28 days after the last dose of IM156
**Visit window**	-	-	-	-	-	-	± 1 day			-	± 3 days	± 1 day	± 7 days	+ 7 days	± 3 days
Informed consent	O														
Confirm Inclusion/Exclusion Criteria	O	O													
Demographic Information and Medical History	O														
Current Medical Conditions	O														
Concomitant Medication History[2]	O														
Prior Chemotherapy and/or treatments for this cancer[3]	O														
Height and Weight/BSA[4]	O	O				O[6]				O[6]		O[6]	O[6]	O	
Physical Examination/Vital Signs[5]	O	O[6]				O[6]	O	O	O	O[6]	O	O[6]	O[6]	O	
ECOG Performance Status Assessment	O	O[6]				O[6]	O	O	O	O[6]	O	O[6]	O[6]	O	

[1] One cycle is composed of 4 weeks (28 days).
[2] Record medications administered to patients within 4 weeks prior to screening.
[3] For history of prior chemotherapies, information on the types and numbers of all the prior treatment lines for the corresponding disease, names of the administered drugs, duration of administration, and reasons for discontinuation will be recorded as far as possible.
[4] Height will be measured at the time of screening only.
[5] Vital signs: systolic and diastolicblood pressure, pulse rate, and temperature.
[6] Should be conducted prior to administration of the IP.

Evaluation Items	Screening	Single dose stage				Cycle 1 (=28 days)[1]				Cycle 2		Cycle ≥3	Cycle ≥13 (every other cycle)	EOT/DC	Safety F/U
	D-21 ~ D-8	D-7	D-6	D-5	D-4	D1	D8	D15	D22	D1	D15	D1	D1	-	28 days after the last dose of IM156
**Visit window**	-	-	-	-	-	-	± 1 day			-	± 3 days	± 1 day	± 7 days	+ 7 days	± 3 days
12-lead ECG	O[7]	O[6]				O[6]				O[6]		O[6]	O[6,8]	O	
**Laboratory Tests**[9]															
- Hematology	O	O[6]				O[6]	O	O	O	O[6]	O	O[6]	O[6]	O	
- Blood Chemistry	O	O[6]				O[6]	O	O	O	O[6]	O	O[6]	O[6]	O	
- Coagulation Tests	O	O[6]				O[6]				O[6]		O[6]	O[6]		
- Urinalysis	O	O[6]				O[6]				O[6]		O[6]	O[6]		

---

7 If outcome values to be measured within 14 days prior to screening are already available, these can be utilized. However, if any clinically significant abnormal finding exists, a 12-lead ECG must be carried out at screening visit.

8 One the first day of just one future cycle, 12-lead ECG test may be performed at the discretion of the investigator prior to and at 4 hr after the IP administration.

9 If outcomes of the laboratory tests to be conducted within 14 days prior to screening are already available, these can be utilized. The items of the laboratory tests are as follows.

① Hematology tests: RBC, hemoglobin, hematocrit, platelet, WBC with differential count (neutrophil, lymphocyte, monocytes, eosinophils, basophils), ANC

② Blood chemistry: Na, K, Cl, Ca, P, glucose, BUN, uric acid, creatinine, total cholesterol, total protein, γ-GTP, LDH, AST, ALT, ALP, total bilirubin, albumin, lactate, HbA1c (HbA1c: To be measured at the screening visit only. Can be measured at subsequent visits according to the investigator's judgment.)

③ Coagulation tests: PT(INR), aPTT

④ Urinalysis: specific gravity, protein, glucose, pH, occult blood, ketone

* Blood tests (Hematology and blood chemistry, etc.) on Day 1 of Cycle 2 and subsequent cycles can be performed within 3 days of Day 1. Based on the results, it can be confirmed whether administration of the IP will continue. At Cycle 13 and subsequent cycles (every other cycle), laboratory tests should be conducted at the discretion of the investigator in accordance with institution's Standard of Care. Lactate must be measured at each study visit.

Evaluation Items	Screening	Single dose stage				Cycle 1 (=28 days)[1]				Cycle 2		Cycle ≥3	Cycle ≥13 (every other cycle)	EOT/DC	Safety F/U
	D-21 ~ D-8	D-7	D-6	D-5	D-4	D1	D8	D15	D22	D1	D15	D1	D1	-	28 days after the last dose of IM156
**Visit window**	-	-	-	-	-	-	± 1 day			-	± 3 days	± 1 day	± 7 days	+ 7 days	± 3 days
- Pregnancy Test[10]	O													O	
- Tumor markers (optional)[11]	O											O[11]	O[11]	O	
**Tumor Assessment**[12]															
- Radiological examination (CT/MRI) (Same test to be used throughout the study)	O											O[12]	O[12]	O	
- Chest X-ray[13]	(O)											(O)[12,13]		(O)	
Pharmacokinetic Evaluation[14]		O	O	O	O	O		O		O			O[15]		
Exploratory Markers Evaluation															

[10] For women of childbearing potential, a serum pregnancy test will be carried out at the time of screening, and a urine test can be conducted at subsequent visits.

[11] Tumor markers (optional): Tumor markers applicable to the corresponding cancers will be tested, if possible, and the test should be performed at every other cycle [e.g., gastric/colorectal cancer - CEA, CA19-9, ovarian cancer - CA-125, liver cancer - APF, prostate cancer – PSA, biliary tract cancer - CA19-9].

[12] Tumor evaluations during screening must be performed within 28 days prior to the initial IP administration (Single dose stage). After the initial administration of the IP, tumor evaluation will be assessed around every 8 weeks (every other cycle ± 3 days) of C1D1. If the tumor assessment has been performed within 28 days prior to the EOT/DC visit, it will not be conducted at EOT/DC visit. In addition, administration of the IP will be interrupted at least for 48 hours prior to the conduct of CT scan using contrast media, and resumed only after confirming serum creatinine level is within the normal range (or baseline value) 48 hours after the CT scan.

[13] A chest X-ray may be performed at the discretion of the investigator.

[14] Patients may be hospitalized in the ward for 24-hour monitoring at Day -7 in single dose stage, Cycle 1 Days 1, and Cycle 2 Day 1 to collect blood and urine for pharmacokinetic evaluation.

[15] One the first day of just one future cycle, collect blood for pharmacokinetic evaluation prior to and at 4 hr after the IP administration.

Evaluation Items	Screening	Single dose stage				Cycle 1 (=28 days)[1]				Cycle 2		Cycle ≥3	Cycle ≥13 (every other cycle)	EOT/DC	Safety F/U
	D-21 ~ D-8	D-7	D-6	D-5	D-4	D1	D8	D15	D22	D1	D15	D1	D1	-	28 days after the last dose of IM156
**Visit window**	-	-	-	-	-	-	± 1 day			-	± 3 days	± 1 day	± 7 days	+ 7 days	± 3 days
- Blood Collection[16]						O		O							
- PET, sestamibi Scan[17]		O[6]										O		O	
- Biopsy (if possible) [18]		O[6]												O	
Administration of IM156[19]		O[20]				D1 ~ D28, QD administration (QD administration during Cohort 6 and all subsequent)									
Drug Compliance Check										O		O	O	O	

[16] Blood collection for exploratory markers evaluation will be conducted on Day 1 (prior to the initial IP administration) and Day 15 of the first cycle [0h (predose), 2h, 4h (only on Day 1)].

[17] PET scan using ^{11}C-acetate and ^{18}F-FDG probe and/or sestamibi scan (PET scan using ^{11}C-acetate probe and sestamibi scan are optional test at the discretion of the investigator) for exploratory markers evaluation will be conducted on Day -7 (scans will be conducted once during the period from the screening period within 14 days prior to the initial IP administration until the time point right before the IP administration is initiated, and this will be considered as the baseline scan) of the single dose stage, the same day tumor assessments are conducted [i.e. every 2 cycles], and at EOT. (rGBM patient is proceeded with a brain PET scan, instead of whole body scan; all scans are optional for rGBM patients). * PET scan using ^{18}F-FDG probe is mandatory, but Sestamibi scan and PET scan using ^{11}C-acetate probe are optional. At or after Cycle 13, PET scan using ^{18}F-FDG probe is optional.

[18] Biopsy for exploratory markers evaluation will be conducted, if available, but should be conducted at the timepoints right before the first administration of the IP (allowed to be conducted from the screening period 14 days prior to the first administration of the investigational product until the timepoint before administration of the IP is initiated) and after completion of the treatment (within EOT visit prior to Safety F/U).

[19] IM156 should be orally administered every other day from Day 1~ Day 28 for each cycle (administration in fasted state [i.e., Patients will be advised to take the IP 2 hours after and 1 hour before eating and drinking except water]). Apply once daily (QD) oral administration from Cohort 6 and all subsequent cochorts.

[20] Following an overnight fast of at least 10 hours, subjects should start high-fat meal 30 minutes prior to administration of IM156. Patients should eat high-fat meal in 20 minutes, and IM156 should be administered with 240 mL of water at 30 minutes after start of the meal. No food should be allowed for at least 4 hours post-dose. Water can be allowed as desired except for one hour before and after the administration of IM156.

**Evaluation Items**	**Screening**	**Single dose stage**				**Cycle 1 (=28 days)[1]**				**Cycle 2**		**Cycle ≥3**	**Cycle ≥13 (every other cycle)**	**EOT/DC**	**Safety F/U**
	**D-21 ~ D-8**	**D-7**	**D-6**	**D-5**	**D-4**	**D1**	**D8**	**D15**	**D22**	**D1**	**D15**	**D1**	**D1**	**-**	**28 days after the last dose of IM156**
**Visit window**	**-**	**-**	**-**	**-**	**-**	**-**	**± 1 day**			**-**	**± 3 days**	**± 1 day**	**± 7 days**	**+ 7 days**	**± 3 days**
Review Concomitant Medications/Therapies		O				O	O	O	O	O	O	O	O	O	O
Assess Adverse Events		O	O	O	O	O	O	O	O	O	O	O	O	O	O
Toxicity Assessment		O	O	O	O	O	O	O	O						

.

List of Abbreviations

ACC	Acetyl Co-A carboxylase
ADME	Absorption, Distribution, Metabolism and Excretion
ADR	Adverse Drug Reaction
AE	Adverse Event
ALP	Alkaline Phosphatase
ALT	Alanine Transaminase
AMPK	AMPK-activated Protein Kinase
ANC	Absolute Neutrophil Count
aPTT	Activated Partial Thromboplastin Time
AST	Aspartate Transaminase
AUC	Area Under the Curve
BA	Bioavailability
BBB	Blood Brain Barrier
BSA	Body Surface Area
BUN	Blood Urea Nitrogen
Ca	Calcium
CA19-9	Carbohydrate Antigen 19-9
CEA	Carcinoembryonic antigen
CI	Confidence Interval
Cl	Chloride
CL/F	Apparent Oral Clearance
C_{max}	Maximum Serum Concentration
C_{min}	Minimum Serum Concentration
CR	Complete Response
CRC	Colorectal Cancer
CT	Computed Tomography
CYP450	Cytochrome P450
DC	Discontinuation
DCR	Disease Control Rate
DLT	Dose Limiting Toxicity
DoR	Duration of Response
ECG	Electrocardiogram
ECOG	Eastern Cooperative Oncology Group
eCRF	electronic Case Report Form
EDC	Electronic Data Capture
EMT	Epithelial-Mesenchymal Transition
EOT	End of Treatment
FAS	Full Analysis Set
FOXC2	Forkhead Box Protein C2

GC	Gastric Cancer
GI50	Growth Inhibition 50
HbA1c	Hemoglobin A1c
ICR	Institute of Cancer Research
INR	International Normalized Ratio
IP	Investigational Product
IRB	Institutional Review Board
K	Potassium
KGCP	Korea Good Clinical Practice
LDH	Lactate Dehydrogenase
MDR1	Multidrug Resistance Protein 1
MedDRA	Medical Dictionary for Regulatory Activities
MFDS	Ministry of Food and Drug Safety
MMP	Matrix Metallopeptidase
MRI	Magnetic Resonance Imaging
MTD	Maximum Tolerated Dose
mTORC1	Mammalian Target of Rapamycin Complex 1
Na	Sodium
NCI-CTCAE	National Cancer Institute-Common Terminology Criteria for Adverse Events
NOAEL	No-Observed-Adverse-Effect level
ORR	Objective Response Rate
OS	Overall Survival
P	Phosphorus
PD	Progressive Disease
PET	Positron Emission Tomography
PFS	Progression-Free Survival
PK	Pharmacokinetics
PPS	Per Protocol Set
PR	Partial Response
PT	Prothrombin Time
PT	Preferred Term
p-gp	P-glycoprotein
QD/qd	Once a Day (daily)
QOD/qod	Every other day
rGBM	Recurrent Glioblastoma
RBC	Red Blood Cell
RECIST	Response Evaluation Criteria for Solid Tumors
rGBM	recurrent Glioblastoma
RANO	Response Assessment in Neuro-Oncology
RP2D	Recommended phase 2 Dose

SAE	Serious Adverse Event
SCLC	Small Cell Lung Cancer
SD	Stable Disease
SOC	System Organ Class
SOP	Standard Operating Procedure
STD10	Severely Toxic Dose 1/10
$T_{1/2}$	Elimination Half-Life
TGFß	Transforming Growth Factor beta
TKI	Tyrosine Kinase Inhibitor
T_{max}	Time of Maximum Concentration
WBC	White Blood Cell
ZEB	Zinc finger E-box-binding Homeobox
γ-GTP	γ-Glutamyltranspeptidase

TABLE OF CONTENTS

REVISION HISTORY OF CLINICAL STUDY PROTOCOL 2
SYNOPSIS 4
STUDY FLOW CHART 14
LIST OF ABBREVIATIONS 19
1. STUDY TITLE AND PHASE 25
1.1. STUDY TITLE 25
1.2. STUDY PHASE 25
2. NAME OF COORDINATING INVESTIGATOR AND SPONSOR 25
2.1. COORDINATING INVESTIGATOR 25
2.2. SPONSOR 25
2.3. ANALYSIS CENTERS 25
3. STUDY OBJECTIVES AND BACKGROUND 26
3.1. STUDY OBJECTIVES 26
3.2. STUDY BACKGROUND 27
3.2.1. Background 27
3.2.2. Rationale for the Clinical Program 27
3.2.3. Anticipated Prophylactic, Therapeutic, or Diagnostic Indications 28
3.2.4. Nonclinical experience 29
3.2.5. Clinical experience 32
4. INVESTIGATIONAL PRODUCTS 33
4.1. OVERVIEW OF INVESTIGATIONAL PRODUCTS 33
4.1.1. Test Drug 33
4.2. MANUFACTURE, PACKAGING, AND LABELLING OF THE INVESTIGATIONAL PRODUCT 33
4.3. MANAGEMENT OF INVESTIGATIONAL PRODUCTS 33
5. TARGET INDICATION AND STUDY DURATION 34
5.1. TARGET INDICATION 34
5.2. STUDY DURATION 34
6. INCLUSION/EXCLUSION CRITERIA FOR PATIENTS, AND TARGET NUMBER OF PATIENTS AND THE RATIONALE 34
6.1. INCLUSION CRITERIA 34
6.2. EXCLUSION CRIETERIA 34
6.3. TARGET NUMBER OF PATIENTS AND BASIS OF CALCULATION 36
7. METHODOLOGY 37
7.1. DESIGN OF STUDY 37
7.1.1. Phase 1a 37
7.1.2. Phase 1b 38
7.2. DOSAGE AND METHOD OF ADMINISTRATION, AND SCHEDULE FOR ADMINISTRATION 38
7.2.1. Dosage and Administration 38
7.2.2. Administration Method 39
7.2.3. Schedule for Administration 39
7.2.4. Rationale for Initial Dose 39
7.3. RATIONALE FOR SELECTION OF CONTROL DRUGS 40
7.4. DOSE LIMITING TOXICITY (DLT) 40
7.4.1. Definition of Dose Limiting Toxicity 40
7.4.2. Safety Committee 40

7.5. DEFINITION AND DETERMINATION OF MAXIMUM TOLERATED DOSE (MTD)....41
7.5.1. Definition of MTD....41
7.5.2. Determination of MTD....41
7.6. DOSE ADJUSTMENT....41
7.6.1. IM156....41
7.7. PRINCIPLE OF CONTINUED ADMINISTRATION....42
7.8. CRITERIA FOR ADMINISTRATION OF CONCOMITANT MEDICATIONS....42
7.8.1. Permitted Concomitant Drugs....42
7.8.2. Prohibited Concomitant Drugs and Treatments....42
7.8.3. Precautions....43
7.9. RANDOMIZATION....43
7.10. MAINTENANCE AND CODE BREAKING OF DOUBLE-BLINDING....43

8. PHARMACOKINETIC AND EXPLORATORY MARKERS EVALUATION....44

8.1. PHARMACOKINETIC EVALUATION....44
8.2. EXPLORATORY MARKERS EVALUATION....44
8.3. BLOOD COLLECTION FOR PHARMACOKINETIC AND EXPLORATORY MARKERS EVALUATION, AND STORAGE AND ANALYTICAL METHODS FOR SPECIMENS....45

9. OBSERVATION ITEMS AND STUDY PROCEDURE....49

9.1. OBSERVATION ITEMS....49
9.1.1. Obtainment of Written Patient Consent and Assignment of Screening Numbers....49
9.1.2. Collection of Demographic and Baseline Information....49
9.1.3. Collection of Medical and Medication Histories Including Prior Chemotherapies....49
9.1.4. Collection of Height, Weight, and BSA Information....49
9.1.5. Collection of Vital Sign Measurements....49
9.1.6. Physical Examination....50
9.1.7. 12-lead ECG....50
9.1.8. ECOG Performance Status....50
9.1.9. Laboratory Tests....50
9.1.10. Tumor Assessments (Radiological examination)....51
9.1.11. Chest X-ray (if necessary)....52
9.1.12. Record Concomitant Medications....52
9.1.13. Record Adverse Events....52
9.1.14. Compliance....52
9.2. STUDY PROCEDURE....52
9.2.1. Screening Visit....52
9.2.2. Single dose stage for food effect study (Day -7)....53
9.2.3. Single dose stage for the food effect study (Day -6, -5, and -4)....54
9.2.4. Cycle 1 Day 1....54
9.2.5. Treatment Visits after Cycle 1 Day 1....54
9.2.6. End of Treatment (EOT) Visit / Early Discontinuation (DC)....55
9.2.7. Safety Follow Up Visit after the End of Treatment (28 days after the last dose of IM156)....56
9.2.8. Unscheduled Visit....56

10. EXPECTED SIDE EFFECTS AND PRECAUTIONS ON USE....57

11. CRITERIA FOR DISCONTINUATION AND WITHDRAWAL....58

11.1. CRITERIA FOR WITHDRAWAL OF PATIENTS....58
11.2. MANAGEMENT OF PROTOCOL VIOLATION....58
11.3. CRITERIA FOR STUDY DISCONTINUATION....58

12. CRITERIA AND METHOD FOR EFFICACY EVALUATION....60

12.1. EVALUATION BASED ON TUMOR ASSESSMENT....60
12.2. EVALUATION ON RESPONSE AND SURVIVAL PERIOD....60

13. CRITERIA AND METHOD FOR SAFETY EVALUATION INCLUDING

ADVERSE EVENTS 61
13.1. CRITERIA AND METHOD FOR SAFETY EVALUATION 61
13.1.1. Adverse Events 61
13.1.2. Vital Signs, Physical Examination, Laboratory Tests, and 12-lead ECG 61
13.2. EVALUATION CRITERIA AND REPORTING METHOD FOR ADVERSE EVENTS 61
13.2.1. Definition of Terms 61
13.2.2. Criteria for Severity Assessment 62
13.2.3. Assessment Criteria for Causality with Investigational Product 62
13.2.4. Reporting Methods 63
13.2.5. Actions to be taken for Serious Adverse Events 64
13.2.6. Progression of Underlying Diseases 64
14. STATISTICAL ANALYSIS METHOD 65
14.1. DEFINITION OF ANALYSIS GROUPS 65
14.2. BASELINE DEMOGRAPHIC DATA AND MEDICAL/MEDICATION HISTORIES 65
14.3. EFFICACY ENDPOINTS 65
14.4. SAFETY ENDPOINTS 66
14.5. PHARMACOKINETIC PARAMETERS 67
14.6. EXPLORATORY MARKER PARAMETERS 67
15. ETHICS 68
15.1. INSTITUTIONAL REVIEW BOARD (IRB) 68
15.2. PATIENT INFORMED CONSENT 68
15.3. CONFIDENTIALITY 68
15.4. SAFETY MEASURES FOR PROTECTION OF PATIENTS 68
15.4.1. Measures to be taken for Adverse Events 68
15.4.2. Medical Treatment and Therapy for Patients after Study 68
15.4.3. Regulation of Compensation for Damage to the Health of Patients 69
16. OTHER REQUIREMENTS FOR SAFE AND SCIENTIFIC CONDUCT OF STUDY 69
16.1. MANAGEMENT AND RETENTION OF RECORDS 69
16.1.1. Source Documents 69
16.1.2. Collection of Study Data 69
16.2. QUALITY CONTROL AND QUALITY ASSURANCE 69
16.2.1. Monitoring of Study Site 69
16.2.2. Audit 70
16.2.3. Inspection 70
16.3. AMENDMENT TO THE PROTOCOL 70
17. REFERENCES 71
18. APPENDICES 73
APPENDIX 1. PRECAUTIONS IN USE OF IM156 74
APPENDIX 2. RESPONSE EVALUATION CRITERIA IN SOLID TUMORS (RECIST) VERSION 1.1 76
APPENDIX 3. RESPONSE ASSESSMENT IN NEURO-ONCOLOGY (RANO) 82
APPENDIX 4. RECIL (2017) 83
19. ATTACHMENTS 84
ATTACHMENT 1. PROTOCOL AGREEMENT 84
ATTACHMENT 2. INVESTIGATIONAL SITE, AND NAMES AND TITLES OF PRINCIPAL INVESTIGATOR · SUBINVESTIGATORS · CLINICAL TRIAL PHARMACISTS 84
ATTACHMENT 3. PATIENT INFORMATION SHEET AND INFORMED CONSENT FORM 84
ATTACHMENT 4. COMPENSATION POLICY FOR VICTIMS 84

1. STUDY TITLE AND PHASE

1.1. Study Title

A Multi Center, Open-label, Phase 1 clinical trial to evaluate the safety, tolerability, and preliminary efficacy of IM156 in patients with advanced solid tumors and lymphoma

1.2. Study Phase

Phase 1

2. NAME OF COORDINATING INVESTIGATOR AND SPONSOR

2.1. Coordinating Investigator

Coordinating investigator: Prof. Sun-Young Rha, Department of Oncology, Yonsei University College of Medicine Severance Hospital

2.2. Sponsor

ImmunoMet Therapeutics

JLABS at Texas Medical Center, 2450 Holcombe Blvd, Houston TX 77021, USA

2.3. Analysis Centers

Analysis of pharmacokinetic (PK) samples

Name of Institution	Address
BioCore Co., Ltd.	IT Mi-Rae Tower 8th & 9th Floor, 33, Digital-Ro 9-Gil, Gasan-Dong, Geumcheon-Gu, Seoul, 08511, Republic of Korea

*Analysis of metabolites will be conducted by a separate institution designated by the sponsor

Analysis of exploratory markers samples

Name of Institution	Address	Remarks
Covance	8211 SciCor Dr, Indianapolis, IN 46214.	VEGF
SPMed	6F, 111 Busan Knowledge Industry Center, Hyoyeol-Ro, Buk-Gu, Busan, Republic of Korea	pS6; pACC; pAMPK; other proteins

*Analysis of other exploratory markers will be conducted by separate institutions designated by the sponsor.

3. STUDY OBJECTIVES AND BACKGROUND

3.1. Study Objectives

1) Primary Objective

- To evaluate the safety and the tolerability of IM156 in patients with advanced solid tumors or lymphoma
- To determine the maximum tolerated dose (MTD) and/or recommended Phase 2 dose (RP2D) in patients with advanced solid tumors or lymphoma

2) Secondary Objectives

① To evaluate the PK characteristics of IM156
② To explore efficacy (tumor response) of IM156

- Objective response rate (ORR)
- Disease control rate (DCR)
- Duration of response (DoR)
- Progression-free survival (PFS)

3) Exploratory Objective

- Identification and profiling of IM156 metabolites
- To evaluate the exploratory markers of IM156
- To evaluate any potential relationship between putative biomarkers and safety/efficacy parameters of IM156

3.2. Study Background

3.2.1. Background

The concept of metabolic cancer therapies originated in 1924 with Otto Heinrich Warburg. Normal cells primarily produce energy through mitochondrial OXPHOS. However, most cancerous cells predominantly produce energy through a high rate of glycolysis followed by lactic acid fermentation, even in the presence of oxygen. This is termed the Warburg effect. Aerobic glycolysis is an inefficient way of producing adenosine triphosphate (ATP) for fast proliferating cancer cells. Transformed cellular metabolism is one of the hallmarks of cancer cells. Metabolism-controlling anticancer drugs, therefore, may result in promising new therapies.

OXPHOS inhibition reduces cellular bioenergetic levels and initiates a cascade of signaling including adenosine monophosphate (AMP)-activated protein kinase (AMPK) activation, which is the master regulator of cell metabolism. AMPK is an energy-sensing enzyme activated when cellular energy levels are low. It signals to stimulate glucose uptake in skeletal muscles, fatty acid oxidation, and reduces hepatic glucose production. AMPK activation suppresses various molecules related to cancer cell signal delivery, making it capable of functioning as an effective cancer therapy. Activation also impedes the regeneration and reproduction of cancer stem cells, which may reduce the risk of drug resistant tumor cells. Lastly, AMPK stimulation controls transcription factors and proteins involved in the epithelial-mesenchymal transition (EMT) process thereby suppressing cancer metastasis.

The anticancer effect of IM156 is driven by combined effect of bioenergetics stress and signaling cascade from AMPK activation and mammalian target of rapamycin (mTOR) inhibition. The anticancer effect can be maximized by combining treatment with traditional anticancer drugs and AMPK activation, preventing or reversing tolerance of cancer cells acquired through EMT and maintaining them susceptible to the anticancer drug.

3.2.2. Rationale for the Clinical Program

IM156 is an improved biguanide to treat cancers in the same class of metformin, which has been used to treat diabetic patients. Many epidemiology studies on metformin-treated diabetic patients suggested a cancer preventive effect of metformin in certain cancers (Zhang & Li, 2014; Evans, et al., 2005; DeCensi, et al., 2010). Published preclinical studies with metformin indicated OXPHOS inhibition is the key mechanism of anticancer activity. Drug resistant tumor cells are very sensitive to OXPHOS inhibition due to the metabolic transition of cancer cells to mitochondrial OXPHOS from glycolysis during drug treatment.

Direct support for the validity of OXPHOS-targeting anticancer therapy comes from a number of reports in the literature investigating cancer metabolism and the role of oxidative phosphorylation. Findings include:

- Mitochondrial markers predict recurrence, metastasis and tamoxifen-resistance in breast cancer. Among 145 estrogen receptor-positive (ER[+]) luminal A breast cancer patients who were treated with tamoxifen, >60 new individual mitochondrial biomarkers predicted treatment failure and tumor recurrence with hazard ratios (HR) of up to 4.17 (p=2.2e-07) (Sotgia, et al., 2017). In lung cancer cases, mitochondrial markers predicted poor overall survival with HR of up to 4.89 (p<1.0e-16) in 726 patients. The authors concluded mitochondria should be therapeutically targeted to improve the effectiveness of current lung cancer therapy and overall survival (Sotgia & Lisanti, 2017). These papers are

representative of a growing body of literature that support the link between mitochondrial status and their use as biomarkers that predict treatment outcomes.

- Elevated OXPHOS activities in resistant cells after chemotherapy or targeted drug treatment (Haq, et al., 2014; Roesch, et al., 2013; Gopal, et al., 2014; Yuan, et al., 2013);
- Upregulation of proteins in OXPHOS such as increased cytochrome C oxidase expression from patients treated with chemotherapy (Vellinga, et al., 2015);
- Increased sensitivity of resistant cells to OXPHOS inhibitors (Roesch, et al., 2013; Jeon, 2016); and
- OXPHOS inhibitors inhibit cancer stem cells (CSCs) known to display high tumorigenic potential (Hirsch, et al., 2009; Pastò, et al., 2014).

Metformin is the best known of the biguanides and a relatively safe OXPHOS inhibitor based on extensive clinical experience. Retrospective studies reported that diabetics treated with metformin were significantly less likely to develop any type of cancer compared to diabetics not taking metformin (Evans, et al., 2005). A number of studies have also demonstrated that biguanides can be effective at antagonizing tumor growth using multiple in vitro and in vivo cancer models (Birsoy, et al., 2014; Buzzai, et al., 2007; Wheaton, et al., 2014).

IM156 is a potent protein complex 1 inhibitor, ~100 times more potent than metformin. Its pharmacokinetic properties are much improved over metformin. IM156 is also cell membrane permeable. Thus it exhibits an anti-cancer effects at much lower doses. Current standards of care (SOC) including chemotherapies and targeted therapies can efficiently kill tumor cells that are highly dependent on the glycolytic pathway. In contrast, drug-resistant cells that emerge post-treatment with a targeted therapy or chemotherapy can often exhibit a metabolic profile distinct from the bulk of the original cancer cell population. These treatment-resistant subpopulations can be highly dependent on mitochondrial metabolic activities of OXPHOS for their biosynthetic and bioenergetic needs, making these resistant cell populations highly susceptible to metabolic regulators targeting OXPHOS (Viale, et al., 2014). This suggests that the sequential or combinatorial application of SOC and OXPHOS inhibitors may be a promising approach to extending progression-free survival (PFS) in cancer patients. Recent studies show molecular signatures of sensitivity to OXPHOS inhibition in various tumor types, which may be employed in biomarker and patient selection strategies in the course of clinical development (Birsoy, et al., 2014; Sancho, et al., 2015; Vayalil & Landar, 2015).

3.2.3. Anticipated Prophylactic, Therapeutic, or Diagnostic Indications

IM156 is a potent OXPHOS inhibitor and AMPK activator that plays suggested key roles in preventing drug resistance, tolerance, impeding metastasis, and inhibiting acquired drug-resistant cancer cells. As such, clinical development includes targeting refractory solid tumors post failure of SOC treatments based on biomarkers indicating OXPHOS sensitivity. Treatment re-sensitizes the tolerance of cancer cells to SOC in combination with IM156 and prevents drug resistance from SOC treatment in combination with IM156. Preclinical efficacy studies support the evaluation of IM156 in refractory gastric cancer and myc+ lymphoma or myc+/Bcl2+ double hit lymphoma.

In the United States (US), approximately 28,000 new cases of gastric cancer are estimated in 2017 (1 million new cases world-wide in 2015), with close to 11,000 deaths per year (723,000 deaths worldwide) attributable to the disease (Siegel, et al., 2017). Fortunately, rates in the US

have been steadily declining over time due to changes in diet and reduced cigarette smoking such that gastric cancer is regarded as an orphan indication in the US. However, gastric cancer has become the third most common cause of cancer death globally with high incidence rates in Asian countries, especially Japan, which has the highest native incidence rate for gastric cancer in the world (80/100,000 vs. 7.5/100,000 in the US) (Siegel, et al., 2017). Although Herceptin is approved as a front-line therapy in gastric cancer for HER2-positive patients, HER2-negative patients do not have options beyond chemotherapy. Typical first-line therapy consists of interchangeable chemotherapies of cisplatin or oxaliplatin, combined with 5 fluorouracil (5-FU), capecitabine or modified docetaxel (Defined Health Primary Research for ImmunoMet on file, National Comprehensive Cancer Network [NCCN]). Second-line therapy in the US is usually a taxane-based therapy, sometimes in combination with ramucirumab, an anti-vascular endothelial growth factor 2 (VEGFR2) monoclonal antibody. Ramucirumab in the second-line treatment provides only a 2.2 month improvement over Taxol with a hazard ratio (HR) 0.8, suggesting there is significant room for improvement (Chan, et al., 2015). Notably, there is no established third-line SOC, although an anti-programmed death-1 (PD-1) agent (nivolumab) is pending approval and is expected to be used in second and possibly third-line settings. However, responses to nivolumab are uncommon and usually short term. In addition, a new biomarker, NFX1, has been identified which indicates sensitivity to OXPHOS inhibition in gastric cancer cell lines. The utility of this biomarker and potentially others will be investigated.

Diffuse large B-cell lymphoma (DLBCL) is the most common form of non-Hodgkin's lymphoma, with 19,650 new cases per year, and it accounts for 30 % of newly diagnosed patients (Sehn, et al., 2007). R-CHOP (a combination of rituximab, cyclophosphamide, doxorubicin hydrochloride, vincristine sulfate, and prednisone) is first-line SOC, yielding around 60-70 % complete response rate. However, the prognosis is poor for patients who relapse or are refractory to frontline R-CHOP. In addition, DLBCL patients with MYC abnormalities represent a large unmet need as their disease is more aggressive and carries a worse prognosis with a 5-year survival rate estimated at only ~30 % (Savage, et al., 2009). The population with MYC protein overexpression represents 30 % of DLBCL case (Thieblemont & Brière, 2013) and one downstream MYC target, miR-17~92, a poly-cistronic microRNA, is over-expressed in 24% of DLBCL (Li, et al., 2009) suggesting a companion biomarker assay for selecting patients at greatest risk. MicroRNAs are abundant non-coding RNAs and modulate gene expression by destabilizing mRNAs. Furthermore, MYC overexpression and miR-17~92^{high} are related since MYC directly enhances miR-17~92 expression (Li, et al., 2009). miR-17~92^{high} has oncogenic characteristics and contributes to MYC-mediated tumorigenesis by rewiring cellular metabolism of MYC-positive cancer cells (Li, et al., 2014). IM156 shows survival extension in an aggressive miR-17~92^{high} B-cell lymphoma model and the data support further evaluation of IM156 in treating miR-17~92^{high} DLBCL patients. In our clinical evaluation, we will further investigate the correlation of clinical response with MYC overexpression and miR-17~92^{high} expression.

3.2.4. Nonclinical experience

1) Efficacy

IM156 provided more potent OXPHOS inhibition (half maximal inhibitory concentration [IC50] of 9 μM in A549) compared to metformin (IC50 of ~500 μM). The IC50 of IM156 on mitochondrial OXPHOS inhibition in the purified complex I membrane from bovine heart showed 2 μM. IM156 is a fast-acting inhibitor, showing OXPHOS inhibition within 20 minutes at the highest concentrations, whereas phenformin does not start to show OXPHOS inhibition until 6 hours at the same concentration, even though their IC50 after 24-hour incubation are almost the same. OXPHOS inhibition initiates a cascade of signaling including AMPK activation which is the master

regulator of cell metabolism. IM156 has been shown to inhibit mTOR through AMPK activation resulting in inhibition of cancer cell growth and proliferation.

Eleven GC lines including MKN-74 showed a significant sensitivity to IM156 as a single agent and further showed increased sensitivity in combination with chemotherapy drugs despite these lines being resistant to chemotherapy alone, suggesting a potential for therapeutic synergy. Gene expression analyses were compared between sensitive and resistant lines to detect a predictive signature. Gene Set Enrichment Assay (GSEA) and elastic net regression analysis identified NFX1 as a predictor in IM156 sensitive GC cell lines. IM156 (10 or 30 mg/kg) also effectively reduced tumor burden in combination with Irinotecan (50 or 75 mg/kg, respectively), a chemotherapy used to treat recurrent or relapsed GC, when evaluated in the Hs746T GC xenograft model in mice. The Hs746T GC line is from the EMT group. Each compound showed ~70% reduction of tumor volume when evaluated alone and the combination of both drugs further decreased tumor volume.

Eµ-MYC lymphoma lines expressing the oncogenic c-MYC are sensitive to OXPHOS inhibitors. Overexpression of miR-17~92 in Eµ-MYC lymphoma increased sensitivity to IM156 or phenformin, although IM156 was much more potent than phenformin. IM156 treatment induced AMPK activation indicated by phosphorylation in parental cells, but miR-17~92 overexpression line blocked AMPK activation upon IM156 treatment which corroborates the potential mechanism of action of IM156 in miR-17~92 overexpression lines. IM156 (10µM) treatment in miR-17~92 overexpression disables the switch to catabolic metabolism. Furthermore, IM156(10 µM) treatment leads to depletion of tricarboxylic acid (TCA) cycle intermediates, which results in energy stress and the inability to supply intermediates of building blocks for anabolic demands and significantly degrades cell viability. The in vitro findings were confirmed in vivo in a xenograft model in mice. The parental line and miR-17~92 overexpression line were inoculated into nude mice and the mice subsequently treated with IM156 in drinking water (0.8 mg/mL dosing). Oral administration of IM156 extended overall animal survival by approximately 50% when compared to animals with the control line or regular drinking water. These results indicate that miR-17~92 expression correlates with sensitivity to OXPHOS inhibition in B-cell lymphoma.

The effect of IM156 on recurrent glioblastoma was examined. Glioblastoma cells were treated with 15 µM of IM156 or metformin control alone or in combination with 500 µM temozolomide. When cells were treated with IM156 and temozolomide together, cell invasion was significantly inhibited compared to control. In addition when cells were treated with IM156 and temozolomide independently, neurosphere size decreased, and when treated together, neurosphere size decreased even further. These results suggest combined treatment with IM156 and temozolomide will effectively inhibit not only cancer stem cell growth, but also metastasis in recurrent glioblastoma. In glioblastoma animal model, the median survival time in the control group was 47 days and 58 days in the temozolomide group. The median survival time in the IM156 group was 82 days, and 106 days in the group administered both IM156 and temozolomide: a large improvement in survival. The temozolomide and IM156 combination increased survival time over IM156 as monotherapy suggesting IM156 may have re-sensitized GSC11 to temozolomide. When brain tissue was examined, it was observed that combined administration led to a decrease in tumor. Therefore, combined administration with IM156 and temozolomide may improve survival rates over the standard treatments by reducing the number of CSCs and inhibiting metastasis.

For details, refer to the investigational brochure of IM156 (ImmunoMet, 2018).

2) Pharmacokinetics and toxicologies

Following once every other day (QOD) oral gavage administration of 50, 100/75, or 150/100 mg/kg to SD rats, the IM156 exposures increased in a dose-related manner but generally in a more than dose-proportional manner based on the area under the curve (AUC) from time zero to time of last measurable concentration (AUC_{last}) values while in a less than dose-proportional manner based on the time to maximal concentration (C_{max}) values. There were no marked (>2 fold) sex differences in the IM156 exposure, although females tended to have marginally higher exposures than those of males across all IM156 dose groups. No major differences in IM156 levels were observed following the first and last dose of IM156, although there may have been some accumulation at all dose levels.

Following QOD oral gavage administration at 20, 40, or 80 mg/kg/dose to beagle dogs, the IM156 systemic exposures (AUC_{last}; C_{max} less affected) generally increased relative to escalating dose while there was no consistent trend to assess dose proportionality due, in part, to large inter-individual variations. There were no marked (>2-fold) sex differences in the IM156 exposures. The QOD administration of IM156 over 27 days resulted in accumulation of IM156 in dogs at 40 and 80 mg/kg/dose, while no apparent accumulation was observed at 20 mg/kg/dose.

The QOD oral gavage administration of 50 mg/kg/dose of IM156 to SD rats over a 27 day period (total of 14 doses) was well tolerated and did not result in any signs of overt toxicity. The QOD oral administration of 150 mg/kg/dose of IM156 resulted in the preterminal euthanasia of 2/15 males and 3/15 females between Days 8 and 10. Additionally, one 150/100 mg/kg/dose female was euthanized on Day 21. IM156-related clinical signs (hypoactivity, hypothermia, abdominal distention, crouching, hair loss, ptosis, piloerection, abnormal breathing, hair color changes, decrease in stool, dehydration, hyposthenia, weight loss, reduction in food consumption), slight body weight loss or reduction in body weight gain associated with decreases in food consumption, changes in hematology, coagulation, and serum biochemistry, as well as organ weights (increased kidney, spleen, and liver) were observed in the animals treated with 100/75 or 150/100 mg/kg/dose of IM156. Therefore, the no-observed-adverse-effect level (NOAEL) of IM156 is 50 mg/kg/dose and the severely toxic dose in 10% of the rats (STD10) is 100 mg/kg/dose, for oral QOD administration over 27 days (total of 14 doses) in rats.

The QOD oral gavage administration of IM156 at 20, 40, or 80 mg/kg/dose to beagle dogs over a 27-day period (total of 14 doses) was tolerated and did not result in morbidity/mortality. At 20 mg/kg/dose, transient clinical signs of salivation, emesis, and diarrhea were observed on the majority of dosing days. At 40 or 80 mg/kg/dose, IM156-related changes included clinical signs of salivation, emesis, diarrhea, decreased activity, and decreased body weight (80 mg/kg/dose); and decreases in thymus weight and increases in spleen weight which correlated with microscopic findings consisting of decreased cellularity of the thymus, slightly higher level of extramedullary hematopoiesis of the spleen, and increased hematopoiesis of the sternal and/or femoral bone marrow. All of these changes were either partially reversible or remained unchanged at the end of a 2week recovery period. Given the transient clinical signs a clear NOAEL could not be assigned. The highest non-severely toxic dose (HNSTD) was ≥80 mg/kg, the highest dose tested, for oral QOD administration over 27 days (total of 14 doses) in dogs.

IM156 was not genotoxic when evaluated in two in vitro assays in the presence and absence of activated S9, or in an in vivo micronucleus test in rodent bone marrow.

For details, refer to the investigational brochure of IM156 (ImmunoMet, 2018).

3) Rationale for the Single-Dose Stage

A single-dose stage was added to the protocol for the start of Cohort 7 to preliminarily assess the effect of a high-fat meal on the safety and PK parameters of IM156. Nausea was noted as a common adverse event in previous cohorts treated with IM156 and may be a class effect for OXPHOS inhibitors (Bayer, Clinical Trial Results Synopsis – Study number 15044). IM156 is currently administered in the fasted state; although this nausea appears well controlled with 5-HT3 receptor antagonists (e.g., ramosteron), it is possible that administration of IM156 with food will increase its tolerability. In addition, the requirement for refraining from eating 2 hours before and 1 hour after IM156 administration may be difficult for some patients. Thus, a single dose of IM156 will be tested 7 days prior to the start of continuous dosing to assess the effect of a hig-fat meal on safety and PK parameters.

3.2.5. Clinical experience

No clinical experience of IM156 in human is available and this study is the first clinical trial in humans.

4. INVESTIGATIONAL PRODUCTS

4.1. Overview of Investigational Products

4.1.1. Test Drug

1) General name: IM156
2) Active Ingredient: IM156
3) Appearance and dosage form:
   - IM156 100 mg, 200mg: A white round tablet
   - IM156 400 mg: A white oblong tablet
4) Storage condition: Store at room temperature (1-30°C) in an airtight container.
5) Shelf life: Maximum 36 months from the manufacturing date (In-house test according to the protocol for stability test)
6) Manufacturer: HanAll BioPharma Co., Ltd.

4.2. Manufacture, Packaging, and Labelling of the Investigational Product

The IP will be supplied to each study site after manufacture or purchase by the sponsor. The IP should be stored separately by batch number until labeled, and the IP label should include the following information according to local regulatory requirements:

1. The statement, “For clinical trial use”
2. Name or identification mark of the IP
3. Batch number or code number of the IP
4. Name, address, and telephone number of the party which has been approved for the clinical study
5. Shelf life (expiration date)
6. Storage condition
7. The statement, “Keep out of reach of children.”
8. Protocol number (Reference code that makes the clinical study identifiable)
9. Patient identification number, IP number, and visit number (Omissible when documented)

4.3. Management of Investigational Products

The sponsor will supply the test drug (IM156) to the clinical trial pharmacists of the study site after completion of the manufacturing and packaging. The clinical trial pharmacists should record receipt of the IP, and store and manage the IP, etc. to ensure it is not used for other purposes than the clinical study.

The sponsor will check the quantity and storage condition of the IP, etc. during the study and take measures to ensure the clinical study can be properly conducted. In addition, the sponsor should collect and discard all unused IP after the clinical study is completed, if the study is discontinued or terminated, or if the Principal Investigators don’t conduct the study in compliance with the protocol.

5. TARGET INDICATION AND STUDY DURATION

5.1. Target Indication

Patients with advanced solid tumors or lymphoma who have failed standard therapy.

5.2. Study Duration

Approximately 36 months from Institutional Review Board (IRB) approval of the protocol (however, this is changeable depending on the rate of patient enrollment).

6. INCLUSION/EXCLUSION CRITERIA FOR PATIENTS, AND TARGET NUMBER OF PATIENTS AND THE RATIONALE

6.1. Inclusion criteria

Patients satisfying the following criteria will be selected as patients.

1) At least 19 years of age
2) Patients with histologically or cytologically confirmed advanced solid tumors or lymphoma
3) Patients for whom no standard therapies are available or who have failed in the existing conventional therapies
4) Patients with measurable or evaluable lesions according to Response Evaluation Criteria in Solid Tumors (RECIST) v1.1 (Response Assessment in Neuro-Oncology [RANO] guideline for recurrent glioblastoma [rGBM], RECIL [2017] for lymphoma)
5) Patients with Eastern Cooperative Oncology Group (ECOG) performance status 0, 1, or 2
6) Patients with adequate function of bone marrow, kidney, and liver as follows
   ① Absolute neutrophil count (ANC) ≥ 1,500/mm^3 (≥ 1,000/mm^3 for lymphoma), Platelet ≥ 100,000/mm^3 (≥ 75,000/mm^3 for lymphoma), Hemoglobin ≥ 9.0 g/dL
   (In case of hemoglobin < 9.0 g/dL, the patient can be enrolled if the value is reversed to ≥ 9.0 g/dL. However, blood transfusion within 1 week of screening is not permitted.)
   ② Serum creatinine ≤ 1.5 × upper limit of normal (ULN)
   ③ Total bilirubin ≤ 1.5 × ULN, aspartate aminotransferase (AST) and alanine aminotransferase (ALT) ≤ 3 × ULN (for patients with liver disease, ≤ 5 × ULN)
   ④ Fasting serum glucose ≤ 160 mg/dL
7) Patients with life expectancy ≥ 12 weeks
8) Patients who have agreed to use acceptable methods for contraception during the study treatment period (e.g.: sterilization of the patient and/or his/her partner, use of intrauterine device by the patient or the partner, barrier contraception such as diaphragm or condom)
9) Patients who have voluntarily signed an informed consent to participate in this clinical study

6.2. Exclusion Crieteria

Patients who are applicable to any of the following criteria will be excluded from the clinical study.

1) Patients with a history of hypersensitivity to the active ingredient or any component of the IP or biguanides
2) Patients with a current evidence of diabetes mellitus who are currently being treated with another biguanide (e.g., metformin)
3) Patients with a history of serious gastrointestinal bleeding within 6 weeks prior to screening or patients with any disease possibly affecting the absorption of oral agents (malabsorption syndrome, hemorrhagic gastric ulcer, etc.)
4) At the time of schedulced starting date of IP administration,
    - At least 4 weeks have not elapsed since any major surgery
    - At least 3 weeks have not elapsed since the last treatment with radiotherapy
    - At least 3 weeks have not elapsed since the last treatment with chemotherapy (at least 6 weeks for nitrosurea compounds)
    - At least 5 half-lives or 3 weeks, whichever is shorter, have not elapsed since the last treatment with biologic agents including hormone therapy
5) Patients whose toxicity due to prior therapy has been restored to baseline or Grade 1 according to the National Cancer Institute (NCI)-Common Terminology Criteria for Adverse Events (CTCAE) v4.03
6) Pregnant or nursing women
7) Patients who were administered another IP within 3 weeks prior to screening
8) Patients with uncontrolled metastasis to the central nervous system. However, patients with treated and stable brain metastases (stable at least for 30 days on radiology imaging) are allowed to enroll.
9) Patients with suspected serious infectious diseases, intestinal paralysis, bowel obstruction, interstitial pneumonia, or pulmonary fibrosis
10) Patients with a history of psychiatric disorders likely to threaten the compliance with this protocol
11) Patients with a history of alcohol or drug abuse within 12 weeks prior to screening
12) Human immunodeficiency virus (HIV) infection or active hepatitis B or C; patients with no detectable viral load may be enrolled.
13) Patients with severe traumatism.
14) Patients with any clinically significant abnormal intestinal findings that may interfere with the administration, passage, or absorption of the IP, which makes the patients unable to orally take the tablet form of drugs
15) Patients with severe cardiac disorders (e.g., myocardial infarction, congestive heart failure, arrhythmia showing dramatic change in ECG, severe or unstable angina, other serious cardiac disorders) or patients with comorbidities of other serious internal disorders (e.g., uncontrolled diabetes mellitus, chronic obstructive pulmonary disorder, renal failure, etc.) based on investigator’s judgment
16) Patients who are otherwise considered to be ineligible for this study based on investigator’s judgment

6.3. Target Number of Patients and Basis of Calculation

1. Phase 1a

In the Phase 1a, 3 or 6 patients are planned to be enrolled at each dose level (approximately 8 dose levels are planned, and cohort may be added following the decision of the Safety Committee) to determine the MTD or RP2D by using a 3+3 design. However, if determination of the dose-limiting toxicity (DLT) among the enrolled patients is infeasible in the first cycle, evaluation will be made by enrolling additional patients. Thus, the total number of patients for the Phase 1a clinical study will not be based upon the test of the statistical hypothesis but dependent on the number of dose escalations.

2. Phase 1b

Since the phase 1b is aimed to make exploratory evaluation on the efficacy (tumor response) and safety of IM156 administered at the RP2D in all patients with solid tumors or lymphoma, the number of subjects satisfying the power to test a statistical hypothesis will not be calculated and approximately 30 subjects with all advanced solid tumors or lymphoma are planned to be enrolled.

7. METHODOLOGY

7.1. Design of Study

This study is a Phase 1 clinical trial to determine the MTD of IM156 and to explore its safety, tolerability, PK characteristics, exploratory markers evaluation, and preliminary efficacy in patients with advanced solid tumors or lymphoma.

The Phase 1 clinical trial consists of 1a and 1b studies.

Phase 1a is conducted to determine the MTD of IM156 as a single agent. In addition, the safety, tolerability, exploratory efficacy, PK characteristics, and exploratory markers of IM156 are evaluated in patients with advanced solid tumors or lymphoma who have failed standard therapies. Approximately 8 cohorts are planned in Phase 1a; the number of cohorts and the dose and schedule of IM156 to be administered at each dose level will be determined by Safety Committee.

The Phase 1b study is conducted to confirm the safety and the tolerability, explore the preliminary efficacy, and evaluate the PK characteristics and exploratory markers at the RP2D of IM156 in patients with advanced solid tumors or lymphoma.

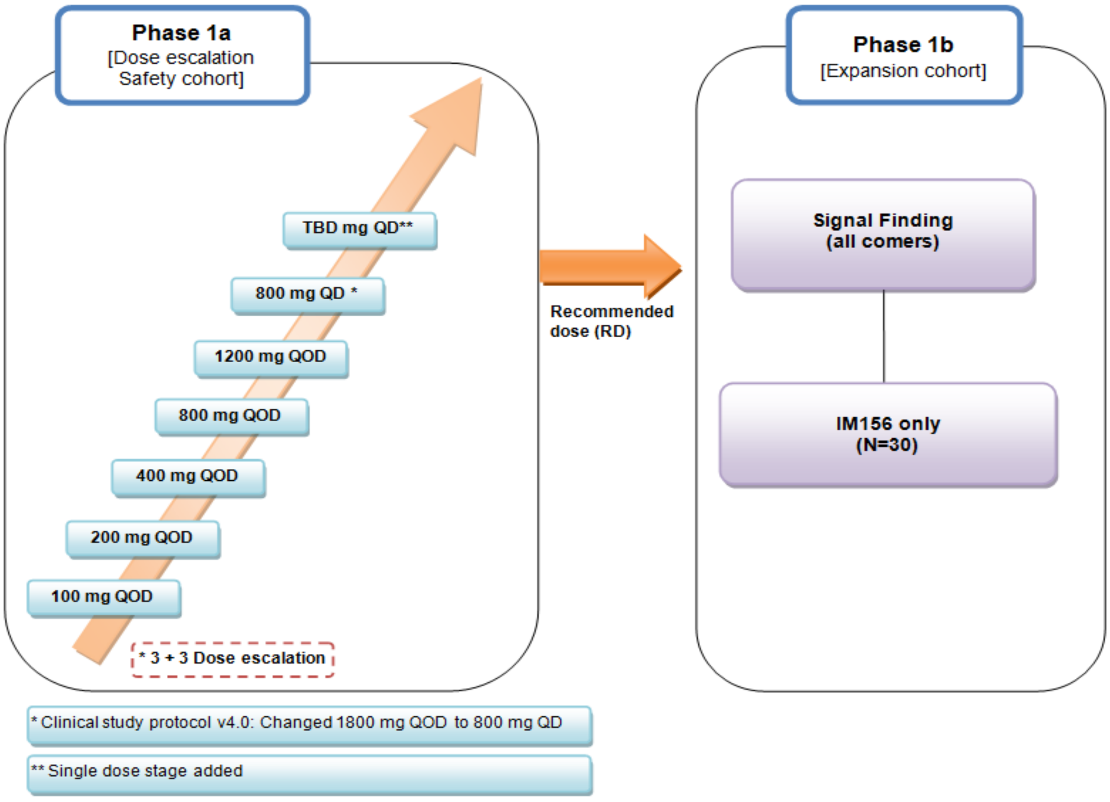

Figure 7.1. Study Scheme

7.1.1. Phase 1a

The study will use a conventional 3+3 design, the dose will be sequentially escalated from the lowest dose. Progression to the next dose cohort will be determined by the Safety Committee based on integration of all the pertinent clinical data obtained during the first cycle including the occurrence of DLTs.

The Saftey Committee can also modify the next dose level; however, the dose escalation will not exceed 100% of the previous dose. If a dose reduction is required when DLT occurs (>2/6), dose can be reduced to the previous dose level or an intermediate dose level between the current and the previous cohort determined by the Safety Committee.

Decisions to dose escalate will be made based on integration of all the pertinent clinical data obtained including the occurrence of DLTs during the first cycle.

The RP2D for IM156 will be either the MTD [Refer to "7.4.1. Definition of Dose Limiting Toxicity", and "7.5. Definition and Determination of Maximum Tolerated Dose (MTD)"] or a lower dose should it be supported by the totality of the data collected and analyzed.A treatment cycle of IM156 consists of 28 days and DLT evaluation will be conducted during the first cycle. All the patients will continue to receive IM156 until unacceptable toxicity occurs, progressive disease according to RECIST guideline v1.1 (RANO for rGBM, RECIL (2017) for lymphoma) is confirmed, or any other reason for discontinuing its administration arises.

7.1.2. Phase 1b

IM156 will be administered at the RP2D and schedule determined during the Phase 1a in a 28-day cycle. Approximately 30 patients will be enrolled in the Phase 1b study. Enrolled patients will continue to receive IM156 until there is an unacceptable toxicity, confirmation of progression disease according to RECIST v1.1 (Rano for rGBM, RECIL (2017) for lymphoma), or any other reason to discontinue administration.

7.2. Dosage and Method of Administration, and Schedule for Administration

7.2.1. Dosage and Administration

1. Phase 1a (Single administration of IM156)

The initial dose level (dose level 1) will be started with IM156 100 mg QOD. One cycle is defined as 28 days and toxicity and evaluation of DLT will be carried out after one cycle of administration is completed. IM156 is planned to be administered at approximately 8 dose levels, and will proceed unless a DLT occurs. Dose escalation will be determined by the Safety Committee after each patient in a cohort has completed one cycle of IM156. Dose escalation will be continued until the MTD is reached or the RP2D is determined.

[Note: After the completion of Cohort 5, the dosing schedule was changed to once daily (QD) (amendment of clinical protocol v4.0)]

① Initial dose: 100 mg

② Dose escalation

Level	Dosage per administration	No. of patients
1	100 mg QOD	3~6
2	200 mg QOD	3~6
3	400 mg QOD	3~6
4	800 mg QOD	3~6

Level	Dosage per administration	No. of patients
5	1200 mg QOD	3~6
6	800 mg QD	3~6
7+	To be determined by Safety Committee	3~6

* Dose escalation to the next level will be carried out through assessment of DLTs after the last patient has completed one cycle, and dose escalation to the next level within the same dose group will not be allowed.

2. Phase 1b

IM156 will be admistered at the RP2D and schedule determined during the Phase 1a in 28-day cycles.

7.2.2. Administration Method

IM156 may be administered either with food or in a fasted state at the discretion of the patient and the investigators. IM156 will be administered with water and swallowed whole without chewing.

In the single dose stage, a single dose of IM156 will be given with a high-fat meal (NITR, 2005). Patients should follow further instructions; following an overnight fast of at least 10 hours, subjects should start high-fat meal 30 minutes prior to administration of IM156. Patients should eat this meal in 20 minutes, and IM156 should be administered with 240 mL of water at 30 minutes after start of the meal. No food should be allowed for at least 4 hours post-dose. Water can be allowed as desired except for one hour before and after the administration of IM156.

If vomiting occurs after administration, its re-administration is not allowed until the scheduled next administration.

7.2.3. Schedule for Administration

IM156 will be administered starting Day 1 of Cycle 1. The treatment cycle is defined as 28 days per cycle.

7.2.4. Rationale for Initial Dose

The starting dose of IM156 has been calculated by following the FDA guideline, ICH S9 (FDA, 2009) using the severely toxic dose in 10% evaluated (STD10) and highest non-severely toxic dose (HNSTD) value. Since IM156 is not a cytotoxic anti-cancer agent, its initial dose for administration for cancer patients in this clinical study has been determined to be 100 mg.

1200 mg is estimated to be the maximum tolerable dose based on the C_{max} value which is the highest concentration reached at a dose level, but dose can be increased up to 3000 mg based on AUC in pharmacokinetic studies.

If humans show higher clearance as predicted by PK simulation data based on preclinical PK data in rats and dogs, the clinically effective dose can be reached as high as 1800 mg; therefore, the 1800 mg level is included in this clinical study.

As a result of the PK simulation for 1200 mg QOD and 1800 mg QOD in addition to QD administration based on the human PK results obtained up to Cohort 4 (800 mg QOD), QD administration of the half dose is able to maintain AUC while lowering the C_{max}. This may reduce the possibility of toxicity due to C_{max}. Also, drug compliance may be increased by switching to QD administration. Therefore, the administration of IM156, which is the investigational product, is switched to once daily (QD) from Cohort 6 onwards. In addition, the initial dose for QD

administration was set to 800 mg, rather than 900 mg QD, allowing direct comparison with 800 mg QOD administration group in addition to increased safety.

[Refer to PK simulation results (refer to the separate document which is the comparison table for the update on Clinical Protocol v4.0)]

7.3. Rationale for Selection of Control Drugs

Since this is a single-arm study, control drug is not applicable.

7.4. Dose Limiting Toxicity (DLT)

7.4.1. Definition of Dose Limiting Toxicity

A DLT is defined as an AE or abnormal clinical laboratory value induced by IM156 administration and limiting gradual dose escalation, applicable to any of the following criteria below, but not associated with PD or intercurrent diseases. DLT evaluation will be performed in the first cycle only to determine the next dose level. Unless otherwise specified, toxicity will be evaluated by using the NCI-CTCAE v4.03. DLTs must be considered as related to IM156 administration.

① Hematologic toxicity

- Grade 4 ANC ≥ 5 days
- Febrile neutropenia as defined by CTCAE v4.03, regardless of the grade
- Grade 4 thrombocytopenia regardless of its duration
- Grade 3 or 4 thrombocytopenia with bleeding

② Non-hematologic toxicity

- Toxicities of Grade 3 or greater (However, alopecia, and nausea, vomiting, and diarrhea of which proper measures for prevention and treatment have not been implemented are excluded.)
- Note: Hypertension ≤ Grade 3 adequately controlled by an anti-hypertensive is not considered a DLT.
- Lactate levels > 5 mmol/L

③ Other

- Administration of at least 70% of the planned dose in QOD dose group or 75% of the planned dose in the QD dose group is infeasible during the administration period due to IP-related toxicities
- Any ≥ Grade 2 significant IP-related toxicity (excluding calcium and phosphorus) that continues for at least 21 days, according to the investigator’s judgment.

7.4.2. Safety Committee

The independent Safety Review Committee will evaluate the safety and tolerability data of each dose to determine the next dose level and the recommended dose for IM156 in Phase 1a. The Safety Committee decides the progression to the next dose level or termination of study. The members of this committee are composed of experts in clinical studies including a clinical pharmacologist, if necessary, but excluding the sponsor and principal investigator of the study site.

7.5. Definition and Determination of Maximum Tolerated Dose (MTD)

7.5.1. Definition of MTD

The MTD will be determined as the highest dose at which one or less than one of 6 patients developes a DLT (≤ 1/6).

7.5.2. Determination of MTD

After DLT evaluation of 3 patients at each dose level,

① If none of the 3 patients have experienced a DLT (0/3):
Dose will be escalated to the next level.

② If one of the 3 patients experiences a DLT (1/3):
Enroll 3 additional patients at the same dose.

- If only one of the 6 patients experiences a DLT (1/3 + 0/3; 1/6): dose will be escalated to the next level.
- If at least two of 6 patients experiences a DLT (≥ 2/6): reduce the dose to one dose level lower and enroll 3 additional patients

③ If two of 3 patients experiences a DLT (≥ 2/3): reduce the dose to one dose level lower and enroll 3 additional patients

If 6 patients have been already evaluated at one lower dose level, no additional patient will be required for evaluation and this one lower level dose will be determined as MTD. However, if two of 3 patients experience a DLT at dose level 1 (starting dose), the clinical study will be terminated.

7.6. Dose Adjustment

7.6.1. IM156

Dose adjustment of IM156 should not be allowed during DLT assessment in Cycle 1 at all dose levels unless clinically necessary. Patients who have dose reductions for reasons other than DLT may be replaced in the dose finding cohorts if they have not taken at least 10 of 14 (70%, for QOD dose group) or 21 of 28 (75%, for QD dose group) of the IM156 doses planned for Cycle 1.

If patients experience DLT-equivalent level of toxicity, administration of IM156 should be temporarily interrupted until the toxicity is recovered to ≤ Grade 1 (non-hematologic toxicities excluding alopecia or fatigue) or baseline level (hematologic toxicities and non-hematologic toxicities present at the time of enrollment in the clinical study), and the treatment may be resumed in the later cycles after reducing the dose according to the following criteria for dose reduction.

Administration of the IP should be discontinued in the patients who experience DLT-equivalent level of toxicity after the dose has been reduced as shown in the following criteria due to IM156-related AEs. After the dose is reduced, the dose should not be increased again in the later cycles.

If a dose cohort is added in accordance with the Safety Committee decision, dose adjustment will be determined based on the added dose level.

[For QOD dosing method]

**Dose level**	**Cohort**					
	**1**	**2**	**3**	**4**	**5**	**6**
**Starting dose**	**100**	**200**	**400**	**800**	**1200**	**1800**
-1	DC	100	200	400	800	1200

-2	DC	DC	100	200	400	800

*DC: Discontinue

[For QD dosing method]

**Dose level**	**Cohort**					
	**6**	**7**				
**Starting dose**	**800**	**TBD***				
-1	600					
-2	400					

*Next dose level will be determined following the decision of the safety committee.

7.7. Principle of Continued Administration

Before initiating each cycle, key hematology test results should be confirmed on Day 1 of each cycle: ANC ≥ 1,500/mm^3 (≥ 1,000/mm^3 for lymphoma) and Platelet count ≥ 100,000/mm^3 (≥ 75,000/mm^3 for lymphoma). At or after Cycle 13, the number of ANC and Platelet should be confirmed on Day 1 of every other cycle.

7.8. Criteria for Administration of Concomitant Medications

7.8.1. Permitted Concomitant Drugs

The drugs listed in the following can be administered concomitantly during the study period.

1) Among the concomitant drugs patients had been taking prior to participation in this clinical study, those considered not to influence the interpretation of the study results can be permitted on investigator’s judgment.
2) Drugs to prevent or treat nausea or vomiting (e.g., ondansetron) are recommended and may be given prophylactically.
3) Anti-diarrheal drugs to control symptoms if diarrhea occurs.
4) Luteinizing hormone-releasing hormone (LHRH) agonist for patients with prostate cancer and premenopausal women with endocrine-dependent breast cancer

Information on all the concomitant drugs (names of ingredients, purpose of administration, dose, duration of drug administration, etc.) should be recorded in the case report forms of patients, if any (including the drugs for other diseases or AEs that have occurred).

7.8.2. Prohibited Concomitant Drugs and Treatments

Except for the list above (Section 7.8.1), combination of the drugs and treatments listed below should be prohibited during the administration of the IP.

1) Anti-cancer therapies including anti-cancer drugs or biologicals other than the IP
2) Immunosuppressants and systemic corticosteroids

   (However, stable doses of prednisone of ≤ 20 mg daily or the equivalent dose of other corticosteroids is allowable for the treatment of non-malignant conditions)
3) Surgical treatments for malignant tumor
4) Radiotherapy (however, radiotherapy for pain relief is allowed if not a target lesion)
5) Other IPs

If prohibited concomitant drugs are required to treat patients during the study according to the investigator's judgment, the patients should be withdrawn from the study immediately and details relevant to this should be recorded in the case report form.

7.8.3. Precautions

The following precautions for metformin which has been widely prescribed and belongs to the same chemical class, biguanide, as IM156 should be referred to and caution should be exercised when interacting drugs are coadministered with IM156.

1) Hypoglycemic agents or drugs with glucose lowering effect (e.g., insulins, sulfonamides, sulfonylureas, α-glucosidase inhibitors, anabolic steroids, guanethidine, salicylates including aspirin, β-blockers including propranolol, angiotensin converting enzyme inhibitors). When these medications are concomitantly administered with biguanides, the risk of hypoglycemia may increase.
2) Examinations requiring IV administration of radiographic iodinated contrast media (e.g., intravenous urography, intravenous cholangiography, angiography, computed tomography using contrast media, etc.). These examinations may cause acute renal failure leading to the accumulation of this medication, which thus has the potential to expose a patient to the risk of lactic acidosis. Therefore, in this clinical study, administration of the IP should be interrupted at least 48 hours prior to the conduct of CT scan and resumed after confirming serum creatinine level is within the normal range (or baseline value) 48 hours after the CT scan.
3) Drinking alcohol or medications containing alcohol. The risk of lactic acidosis increases with acute alcoholic intoxication which may occur under the status of fasting, malnutrition, or decreased liver function.
4) Carbonic anhydrase inhibitors (e.g., zonisamide, acetazolamide, dichlorophenamide). If this medication is concomitantly administered with carbonic anhydrase inhibitors, metabolic acidosis may be caused, and the risk of lactic acidosis may increase.
5) Drugs that may cause interactions (e.g., furosemide, nifedipine, cimetidine), affect renal function, or cause significant hemodynamic changes, or affect this medication like cationic drugs excreted by renal tubular secretion (e.g., amiloride, digoxin, morphine, procainamide, quinidine, quinine, ranitidine, triamterene, trimethoprim, vancomycin). The applicable medications may have an impact on the absorption and metabolism of biguanides resulting in the increase in plasma concentration of IM156.
6) Glyburide. If coadministered with biguanides, it is likely that plasma concentration of glyburide is decreased.

7.9. Randomization

Randomization is not applicable in this study.

7.10. Maintenance and Code Breaking of Double-Blinding

Blinding is not applicable to this study.

8. PHARMACOKINETIC AND EXPLORATORY MARKERS EVALUATION

8.1. Pharmacokinetic Evaluation

Pharmacokinetic evaluation on IM156 will be carried out at each dose level in all the patients enrolled in the Phase 1a and Phase 1b study. Pharmacokinetic evaluation will be performed on Day 1 and 15 (Day 15 is only for the Phase 1a study) of the first cycle (Cycle 1), and Day 1 of the second cycle (Cycle 2). Day 1 Cycle 2 will be Day 29. On Day 1 of Cycles 1 and 2, patients may be hospitalized in the ward where 24-hour monitoring is possible, and blood and urine will be collected before and after IM156 adminstration for PK evaluation. The drug concentration will be evaluated whenever analyses of patient samples are feasible during the clinical study. In PK evaluation, area under the concentration-time curve (AUC) over the time interval from 0 extrapolated to infinity (AUC_{inf}), AUC up to the last measurable concentration ($AUC_{0–last}$), maximum plasma drug concentration (C_{max}), minimum plasma drug concentration (C_{min}), time taken to reach the maximum concentration (T_{max}), terminal phase half life ($T_{1/2}$), apparent total clearance of the drug from plasma after oral administration (CL/F), accumulation ratio at steady state, cumurative amount of unchanged drug excreted into the urine (Ae), renal clearance (CLr), and so on will be determined and other PK parameters can be added, if necessary. The time points for blood collection in the phase 1b study are changeable based upon the results of the phase 1a study.

Only at the MTD in the Phase 1a study, exploratory assessment of IM156 metabolites will be conducted using pooled plasma samples and urine samples, and pertinent details will be reported separately from this clinical study report.

8.2. Exploratory Markers Evaluation

Exploratory markers will be evaluated using peripheral blood mononuclear cells (PBMCs) from all enrolled patients on Days 1 and 15 of the first cycle at each dose level during Phase 1a. Molecular imaging marker evaluation will be carried out on Day 1 of the first cycle which is defined as baseline scan (Scan is acceptable as a baseline scan collected within 14 days prior to the initial IP administration during screening period), the same day tumor assessments are conducted [i.e. every 2 cycles], and at EOT. Sestamibi scan is optional at the discretion of the investigator. PET scan using ^{11}C-acetate probe is optional from Cohort 6 at the discretion of the investigator.

GBM patient is proceeded with a brain PET scan, instead of the whole-body scan.

In addition, tumor tissue should be collected from patients who agree on the ICF before IP administration is initiated (possible to be conducted during screening period within 14 days before IP administration) and after EOT (prior to the end of safety F/U visit). Tumor biopsy will be used to evaluate exploratory biomarkers.

① Molecular imaging markers
PET scans using ^{11}C-acetate (^{11}C-acetate PET scan is optional from Cohort 6 at the discretion of the investigator) and ^{18}F-FDG probe, and/or Sestamibi scan (Sestamibi scan is optional at the discretion of investigator) will be used to evaluate the change in tumor energy metabolism. *PET scan using ^{18}F-FDG probe is mandatory, but Sestamibi scan and PET scan using ^{11}C-acetate probe are optional. ^{18}F-FDG-PET scans are not required for patients with GBM.

② Target related markers
We will assess exploratory markers which are related to effects of IM156 on the mitochondria through IHC. If possible, archived tumor samples will be obtained to assess for potential biomarkers.

③ Safety markers
Blood lactate level will be assessed using a portable lactate monitor.

④ Mechanism surrogate markers

8.3. Multiple potential markers of IM156 effects will be investigated, including those with preliminary data (e.g., Vascular endothelial growth factor levels, phosphorylation status of ribosomal protein S6 and acetyl Co-A carboxylase) as well as, but not limited to, potential markers of metabolism-related analytes, mechanism-related proteins such as redox proteins, TCA cycle-, lipid oxidation-, oxidative phosphorylation-, and tRNA ligase-enzymes, etc. and their products.Blood Collection for Pharmacokinetic and Exploratory Markers Evaluation, and Storage and Analytical Methods for Specimens

1) Method for blood collection, and pretreatment and storage condition of separated plasma

During blood collections performed during hospitalization for PK and exploratory markers evaluation, pain and impact caused by frequent blood collection should be minimized with placement of a catheter into the available brachial vein. At the time of blood collection using a catheter at the time points specified in Table 8.3.1, about 1.0 mL of normal saline should be infused into the catheter after drawing about 1.0 mL of blood to prevent blood coagulation. Fully sterilized disposable device and fluid should be used to prevent infection. The guideline on detailed procedures will be separately provided.

Table 8.3.1. Time points of collecting blood for pharmacokinetic and exploratory markers evaluation

Sample No.		Cycle	Day	Amount of blood (ml)	Time points for blood collection (Time after administration of IM156)
Phase 1a	Phase 1b*				
01	-	SDay**	-7	5	0 (right before administration of IM156)[1)]
02	-	SDay**	-7	5	30 minutes (± 5 minutes)
03	-	SDay**	-7	5	1 hour (± 5 minutes)
04	-	SDay**	-7	5	2 hours (± 10 minutes)
05	-	SDay**	-7	5	4 hours (± 15 minutes)
06	-	SDay**	-7	5	8 hours (± 30 minutes)
07	-	SDay**	-7	5	12 hours (± 1 hour)
08	-	SDay**	-6	5	24 hours (± 2 hour)
09	-	SDay**	-5	5	48 hours (± 2 hour)
10	-	SDay**	-4	5	72 hours (± 2 hour)
11	31	1	1	10	0 (right before administration of IM156)[1,2)]
12	32	1	1	5	30 minutes (± 5 minutes)
13	33	1	1	5	1 hour (± 5 minutes)
14	34	1	1	10	2 hours[2)] (± 10 minutes)
15	35	1	1	10	4 hours[2)] (± 15 minutes)
16	36	1	1	5	8 hours (± 30 minutes)
17	37	1	1	5	12 hours (± 1 hour)
18	38	1	2	5	24 hours (- 2 hours, before administration of IM156)
19	39	1	15	10	0 (right before administration of IM156, -

Sample No.		Cycle	Day	Amount of blood (ml)	Time points for blood collection (Time after administration of IM156)
Phase 1a	Phase 1b*				
					10 minutes)[2)]
20	40	1	15	10	2 hours [2)] (± 10 minutes)
21	41	2	1	5	0 (right before administration of IM156, -10 minutes)
22	42	2	1	5	30 minutes (± 5 minutes)
23	43	2	1	5	1 hour (± 5 minutes)
24	44	2	1	5	2 hours (± 10 minutes)
25	45	2	1	5	4 hours (± 15 minutes)
26	46	2	1	5	8 hours (± 30 minutes)
27	47	2	1	5	12 hours (± 1 hour)
28	48	2	2	5	24 hours (- 2 hours, before administration of IM156)
29	-	≥13[3]	1	5	0 (right before administration of IM156, -10 minutes)
30	-	≥13[3]	1	5	4 hours (± 15 minutes)

* The time points for blood collection in the phase 1b study are changeable based upon the results of the phase 1a study.
** SDay: Single dose stage for food effect study.
[1)] The allowable window in the time points for blood collection will not be applied to the blood collection before the initial administration of IM156 (0h on Day 1 of Cycle 1). Therefore, the blood samples will be obtained at any time available for blood collection prior to the first administration of IM156.

[2)] Exploratory markers evaluation along with pharmacokinetic evaluation will be performed at 0 h, 2 h, and 4 h of Day 1, and 0 h and 2 h of Day 15. At other time points, pharmacokinetic evaluation will be performed.

[3)] A plasma sample for PK analysis will be obtained at prior to and 4 hours (the approximate T_{max}) after the IP administration, on Day 1 of Cycle 13 or later.

For PK analyisis, the collected blood should be immediatedly stored in an ice box and supernatant plasma should be separated by centrifugation within a maximum of 45 minutes. For exploratory marker analysis in PBMCs, the collected blood should be moved to the laboratory at room temperature and PBMCs should be separated by centrifugation within a maximum of 45 minutes. The aliquoted specimen should be transferred to a freezer and stored at ≤ -70 ℃ until transported. Recordings of temperature and specimen control should be maintained. The guideline on detailed procedures for this will be provided separately.

2) Method, pretreatment and storage method for urine collection

The study patients should be instructed to empty their bladders prior to IP administration for the collection of urine samples. During hospitalization, the patients will be required to urinate prior to drug administration (spot urine) and collect urine in a provided storage container at established time points as in Table 8.3.2 post-administration.

Table 8.3.2. Time points of collecting urine for pharmacokinetic evaluation

Sample No.		Cycle	Day	Period of urine collection (Time after administration of IM156)
Phase 1a	Phase 1b*			
01	-	SDay**	-7	0 (Before administration of IM156, spot urine)
02	-	SDay**	-7	0~4 hours<br>(Collection of all urine available within the period)
03	-	SDay**	-7	4~12 hours<br>(Collection of all urine available within the period)
04	-	SDay**	-7	12~24hours<br>(Collection of all urine available within the period)
05	13	1	1	0 (Before administration of IM156, spot urine)
0	14	1	1	0~4 hours<br>(Collection of all urine available within the period)
07	15	1	1	4~12 hours<br>(Collection of all urine available within the period)
08	16	1	1	12~24hours<br>(Collection of all urine available within the period)
09	17	2	1	0 (Before administration of IM156, spot urine)
10	18	2	1	0~4 hours<br>(Collection of all urine available within the period)
11	19	2	1	4~12 hours<br>(Collection of all urine available within the period)
12	20	2	1	12~24hours<br>(Collection of all urine available within the period)

* The time points for urine collection in the phase 1b study are changeable based upon the results of the phase 1a study.

** SDay: Single dose stage for food effect study.

Urine samples should be stored in a refrigerator (1~8 ℃) during the collection. Patients should be instructed to collect urine at the end of each time window indicated in the table 8.3.2, if possible. Total volume of urine for each time interval will be recorded in the unit of mL. The aliquoted specimen should be transferred to a freezer and stored at ≤ -70 ℃ until transported to the analysis center. Recordings of temperature and specimen control should be maintained. The guideline on detailed procedures will be provided separately.

3) Storage method for tumor tissue specimen

Tumor biopsies (primary or metastatic tumor) will be collected prior to administration of the IP and after EOT, if available. Tumor tissue specimens should be prepared at the study site and sent to the sponsor or its designee according to the detailed procedures provided separately. The specimens can be stored for up to 10 years after the clinical study is completed, and the guidelines on the storage of specimens will be provided separately. Archived tumor samples, ideally from the most recent biopsy, will be provided, if available.

4) Transportation of samples for pharmacokinetic and exploratory marker analysis

Details of transferring and storing samples including the address and contact information to which they should be transported will be provided to the study site in a separate guideline by the sponsor.

5) Analytical method for IM156

Operating condition for analysis of IM156 in plasma/urine should be established and conducted using liquid chromatography-tandem mass spectrometry (LC-MS/MS). The established analytical method should be applied to the analyses of samples after validation of its specificity, linearity, accuracy, precision, and sensitivity has been completed. The remaining samples after the analysis of IM156 will be discarded in accordance with the procedure of the analysis center when the analysis is completed.

9. OBSERVATION ITEMS AND STUDY PROCEDURE

9.1. Observation Items

9.1.1. Obtainment of Written Patient Consent and Assignment of Screening Numbers

Prior to participation in the clinical study, the objectives and contents of this clinical study should be explained to the patients (or their representatives) in detail and written informed consents should be obtained. The written informed consent should be obtained prior to any procedure of the clinical study and the investigators should provide a copy of the signed informed consent to the patients.

After the informed consent form is signed, the investigators should assign a screening number to the patients according to the order the consent forms were obtained. If any patient assigned with a screening number withdraws during screening, the corresponding screening code should not be reassigned to a different patient.

9.1.2. Collection of Demographic and Baseline Information

Initials, ages, and gender information of the patients should be collected at screening visit.

9.1.3. Collection of Medical and Medication Histories Including Prior Chemotherapies

Medical and medication histories of the patients should be investigated and recorded in detail through a medical examination and interview and a review of their previous medical records. Existence of the past and current histories of surgeries, chemotherapies, radiotherapies, etc. within 4 weeks prior to screening should be investigated and their times of occurrence and the investigators' comments should be recorded in the Medical History section of the case report form.

For medication history, information on medications administered to the patients within 4 weeks prior to screening including names of the medications (names of active ingredients), purpose of administration, daily dose, and route and duration of administration should be investigated. However, for the history of chemotherapies, information on the types and numbers of all the previous treatment lines available for the pertinent disease, and for the medications administered, names of the drugs, duration of administration, and reasons for discontinuation should be investigated.

9.1.4. Collection of Height, Weight, and BSA Information

Height (in cm) should be measured at the time of screening only, and weight (kg) and body surface area (BSA) (m^2) can be measured and calculated on the first day (Day 1) of each cycle (at or after Cycle 13, weight and BSA will be measured on Day 1 of every other cycle).

9.1.5. Collection of Vital Sign Measurements

For vital signs, systolic and diastolic blood pressure, pulse rate, and body temperature should be measured at each visit to the study site. Vital signs should be measured prior to other scheduled tests after the patient has rested for at least 5 minutes in a sitting position (at or after Cycle 13, the vital signs will be measured on Day 1 of every other cycle).

9.1.6. Physical Examination

Physical examination should be conducted at each visit to the study site. The examination of the appearance of skin, head/neck, chest/lungs, heart, abdomen, urinary/reproductive system, extremity, musculoskeletal system, nervous system, lymph nodes, and other body organs are required, and any findings in the physical examination after administration of IP applicable to the definition of AEs should be reported as AEs (at or after Cycle 13, the physical examination will be conducted on Day 1 of every other cycle).

9.1.7. 12-lead ECG

12-lead ECG should be carried out at the time of screening, on the first day (Day 1) of each cycle, and at EOT/DC. However previous measurements within 14 days prior to screening can replace the ECG requirement at screening. However, ECG should be performed at screening visit if any clinically significant abnormal finding exists. If any clinically significant abnormal finding appears in the ECG at the time of screening, enrollment in the clinical study should be based on the investigator’s judgment (at or after Cycle 13, 12-lead ECG will be performed on Day 1 of every other cycle. One the first day of just one future cycle (once Amendment 6 is implemented), 12-lead ECG test may be performed at the discretion of the investigator prior to and at 4 hr after the IP administration.).

9.1.8. ECOG Performance Status

The ECOG Performance Status should be evaluated at each visit during the treatment period including the screening and at the time of EOT/DC.

Grade	ECOG Performance Status
0	Fully active, able to carry on all pre-disease performance without restriction
1	Restricted in physically strenuous activity but ambulatory and able to carry out work of a light or sedentary nature (e.g., light house work, office work)
2	Ambulatory and capable of all selfcare but unable to carry out any work activities, capable of activities about more than 50% of waking hours
3	Capable of only limited selfcare, confined to bed or chair more than 50% of waking hours
4	Completely disabled, cannot carry on any selfcare, totally confined to bed or chair

9.1.9. Laboratory Tests

Screening tests can be replaced with the measurements within 14 days prior to the screening, if available. However, if any clinically significant abnormal finding exists, the laboratory tests should be conducted at the screening visit.

The laboratory tests will be carried out at the investigational sites and the following test items will be included.

Hematology and blood chemistry will be carried out at each visit, and coagulation tests and urinalysis at screening and prior to initiation of each cycle. However, after Cycle 3, blood chemistry will be performed prior to initiation of each cycle to confirm whether IP administration can be continued.

In addition, blood tests (Hematology and blood chemistry, etc.) will be conducted on Day 1 with 3-day window for Cycle 2 and subsequent cycles. The blood test results will be used to determine the continuation of IP administration. At or after Cycle 13 (once Amendment 6 is

implemented), laboratory tests will be conducted at the discretion of the investigator in accordance with the institutino's Standard of Care. Lactate is to be measured at each study visit.

- Hematology: RBC, hemoglobin, hematocrit, platelet, WBC with differential count (neutrophils, lymphocyte, monocytes, eosinophils, basophils), ANC
- Blood chemistry: Na, K, Cl, Ca, P, glucose, BUN, uric acid, creatinine, total cholesterol, total protein, γ-GTP, LDH, AST, ALT, ALP, total bilirubin, albumin, lactate, HbA1c (HbA1c: To be conducted at screening visit only. Can be conducted at subsequent visits, if necessary, on investigator's judgment). Lactate will be measured using a portable lactate monitor.
- Coagulation tests: PT(INR), aPTT
- Urinalysis: specific gravity, protein, glucose, pH, occult blood, ketone
- Pregnancy test: Serum pregnancy test should be conducted for women with childbearing potential at the time of screening, and urine test can be carried out at the other visits.
- Tumor marker (optional): Tumor markers applicable to the corresponding cancers should be tested at screening and at every other cycle, if possible [e.g., gastric/colorectal cancer - CEA, CA19-9, ovarian cancer - CA-125, liver cancer - APF, prostate cancer – PSA, biliary tract cancer - CA19-9].

9.1.10. Tumor Assessments (Radiological examination)

Exploratory assessment of the tumor response from IM156 monotherapy should be conducted in compliance with the guidance of RECIST ver.1.1 (RANO for rGBM, RECIL (2017) for lymphoma). Tumor assessment should be made at least once prior to initiation of IP administration and each lesion should be evaluated during the study period in the same manner. Tumor assessments will be performed at every other cycle (approximately 8 weeks interval). Unexpected tumor assessment within 28 days prior to EOT/DC visit can replace the tumor assessment requirement at EOT.

Tumor assessment will be conducted using a CT scan or MRI scan. MRI scan for rGBM should be used. Whichever method is used at screening, the same method should always be used for tumor assessments including at EOT. Tumor assessment during screening will be used as a baseline measurement to evaluate the future response and/or progression. The study site should follow the procedure below to ensure the reasonable comparison of tumor data and the uniformity of tumor response evaluation during the study period.

- All the lesions identified at baseline should be reassessed using the same method in all the follow-up evaluation throughout the study process (i.e., if a contrast agent was used for CT scan at baseline, the same contrast agent should be always used in all subsequent CT scans. However, in case a CT scan is not possible due to hypersensitivity to contrast agents, MRI scan is acceptable.).

- Imaging data obtained from all patients enrolled in the study site should be reviewed by the investigators together with radiologists within the study site, who will determine disease assessment.

Besides the scheduled tumor assessments, unscheduled imaging studies are allowed based on clinical necessity determined by investigator.

Tumor assessment will be carried out once around every 8 weeks (every other cycle ± 3 days). If outcomes of the tumor assessment which are conducted within 14 days prior to screening are

already available, these can be utilized. If the tumor assessment has been performed within 28 days prior to the EOT/DC visit, it will not be conducted at EOT/DC visit.

Sestamibi, PET scan using ^{18}F-FDG or ^{11}C-acetate probe (PET scan using ^{11}C-acetate probe and Sestamibi scan are optional at the discretion of the investigator) will be additionally performed for exploratory markers evaluation and refer to the Section 8.2. GBM patients are proceeded with a brain PET scan, instead of whole body scan, and all scans are optional for rGBM patients. At or after Cycle 13, PET scan using 18F-FDG probe is optional at the discretion of the investigator.

9.1.11. Chest X-ray (if necessary)

If chest CT scan is not performed to evaluate the disease, chest X-ray can be carried out at screening and at the end of every 2 cycles (at an interval of about 8 weeks). Additional chest x-rays can be carried out if the investigators consider clinically necessary.

9.1.12. Record Concomitant Medications

When concomitant medications need to be administered, names of the drugs (names of active ingredients), purpose of administration, daily dose (or one time), and route and duration of administration should be investigated at each visit during the study.

9.1.13. Record Adverse Events

AEs should be investigated and recorded in the case report form at each visit. In addition, if the severity of symptoms or signs identified before initial IP administration has deteriorated after IP administration, they should be considered as AEs.

AEs will be collected from the time of initial IP administration until 28 ± 3 days after the last administration of the IP (safety follow-up visit after EOT).

Criteria for assessment and method for reporting of AEs are described in detail in the Section 13.2.

9.1.14. Compliance

Compliance with oral administration of IP should be assessed at the end of each cycle and the number of tablets which have not been taken by the patients should be checked and recorded.

9.2. Study Procedure

9.2.1. Screening Visit

The patients who have been asked to participate in this study will have the study explained to them and evaluated in the following order.

1) Before any procedure of the study is initiated, an explanation of the study will be provided to all the patients and written informed consent will be obtained from the patients.
2) Screening numbers will be assigned to the patients.
3) Demographic information, past/current medical history, and medication history (including the information on prior chemotherapies) of the patients will be obtained.
4) Height and weight/BSA will be measured.

5) Vital signs (systolic and diastolic blood pressure, pulse rate, and body temperature) will be measured and physical examination will be carried out.
6) 12-lead ECG will be performed (this can be replaced with the outcome values measured within 14 days prior to screening, if available. However, if any clinically significant abnormal finding exists, it must be carried out at screening visit).
7) ECOG Performance Status will be assessed.
8) Laboratory tests (hematology, blood chemistry, coagulation tests, urinalysis, and tumor markers test [if possible]) will be performed (only for screening tests, the outcomes measured within 14 days prior to screening can be utilized, if available. However, if any clinically significant abnormal finding exists, the laboratory tests will be conducted at screening visit).
9) For women of childbearing potential, pregnancy test will be performed.
10) Tumor assessment will be conducted (alternatively measurements within 4 weeks prior to screening can be utilized).
11) The inclusion/exclusion criteria will be checked.
12) The next visit will be scheduled.

9.2.2. Single dose stage for food effect study (Day -7)

All patients who are eligible to participate in the clnical study after screening tests can be enrolled in the clinical tudy and prescribed the IP.

1) Patients should have an overnight fast of at least 10 hours.
2) Vital signs (systolic and diastolic blood pressure, pulse rate, and body temperature) and weight/BSA will be measured and physical examination will be conducted.
3) ECOG Performance Status will be assessed.
4) 12-lead ECG will be performed.
5) Laboratory tests (hematology, blood chemistry, coagulation tests, and urinalysis) will be performed.
6) Concomitant medications/therapies will be checked.
7) The inclusion/ exclusion criteria will be re-checked before enrolling the patients to the study.
8) Scans (PET scan using ^{18}F-FDG probe is mandatory, Sestamibi scan and PET scan using ^{11}C-acetate probe is optional at the discretion of the investigator) for exploratory markers evaluation will be also performed.
9) For exploratory markers evaluation, biopsy will be collected (baseline biopsy may be collected from the time the patient signs the ICF until the first dose of IP is administered).
10) IP will be administered. Patients should start a high-fat meal (NITR, 2005) in 20 minutes starting 30 minutes prior to the administration of IM156 with 240 mL of water.
11) Samples for pharmacokinetic evaluation will be collected (for collection of pharmacokinetic samples, the patients may be hospitalized one day prior to this visit, if possible. For the time points of sample collection, refer to the Section 8).
12) No food will be eaten for at least 4 hours after the administration of IM156. Water can be allowed as desired except for one hour before and after the administration of IM156 during the food effect study.
13) AEs will be checked.
14) The next visit will be scheduled.

9.2.3. Single dose stage for the food effect study (Day -6, -5, and -4)

The following procedures will be performed;

1) Samples for pharmacokinetic evaluation will be collected (for the timepoints of sample collection, refer to Section 8).
2) AEs will be checked.
3) The next visit will be scheduled.

9.2.4. Cycle 1 Day 1

The procedures at the scheduled visits are as follows.

1) Vital signs (systolic and diastolic blood pressure, pulse rate, and body temperature) and weight/BSA will be measured and physical examination will be conducted.
2) ECOG Performance Status will be assessed.
3) 12-lead ECG will be performed.
4) Laboratory tests (hematology, blood chemistry, coagulation tests, and urinalysis) will be performed.
5) Concomitant medications/therapies will be checked.
6) AEs will be checked.
7) Samples for pharmacokinetic/exploratory markers evaluation will be collected (for collection of pharmacokinetic samples, the patients may be hospitalized one day prior to Cycle1 Day1, if possible. For the time points of sample collection, refer to the Section 8).
8) The IPs will be distributed. IM156 will be administered in a fasted state (patient will be advised to take IP at least 2 hours after and 1 hour before eating or drinking except water).
9) The next visit will be scheduled.

9.2.5. Treatment Visits after Cycle 1 Day 1

One treatment cycle consists of 28 days (4 weeks), and administration of the IP should be continued until unacceptable toxicity occurs, the progressive disease according to RECIST v1.1 (RANO for rGBM, RECIL (2017) for lymphoma) is confirmed, and any reason for discontinuing their administration arises. Until the patients discontinue the administration of the IP, they are requested to visit the study site weekly (± 1 day) during the first cycle (Cycle 1), biweekly during the second cycle (Cycle 2), and monthly during the third cycle and subsequent cycles. At or after Cycle 13 (once Amendment 6 is implemented), the patient will visit the study site on Day 1 of every other cycle for study assessments. The following procedures will be carried out at each visit after baseline. [There is no visit window on Day 1 of Cycle 2. Visit window for Day 15 of Cycle 2 is ± 3 days, and Day 1 on Cycle 3 and subsequent cycles is ± 1 day. For the study assessment visits to occur at or after Cycle 13 (once Amendment 6 is implemented), the visit window is ± 7 days.]

1) Vital signs (systolic and diastolic blood pressure, pulse rate, and body temperature) and weight/BSA (measured only on Day 1 of each cycle) will be measured and physical examination will be performed. At or after Cycle 13 (once Amendment 6 is implemented), vital signs and weight/BSA will be measured at each study visit.
2) ECOG Performance Status will be assessed.
3) 12-lead ECG will be performed (On Day 1 of each cycle); At or after Cycle 13 (once Amendment 6 is implemented), a 12-lead ECG will be performed at each study visit. One

the first day of just one future cycle (once Amendment 6 is implemented), 12-lead ECG test may be performed at the discretion of the investigator prior to and at 4 hr after the IP administration.

4) Laboratory tests will be conducted. At or after Cycle 13 (once Amendment 6 is implemented), laboratory tests will be conducted at the discretion of the investigator in accordance with the institution’s Standard of Care. Lactate is to be measured at each study visit.
   - Hematology and blood chemistry: At each visit
   - Coagulation tests and urinalysis will be conducted on Day 1 of each cycle.
   - Tumor markers (optional) will be evaluated at every other cycle.
5) Tumor assessment and scans for exploratory markers evaluation (PET scan using 18F-FDG probe is mandatory, Sestamibi scan and PET scan using 11C-acetate probe are optional at the discretion of the investigator) will be conducted with every 2-cycle interval (8 weeks). At or after Cycle 13 (once Amendment 6 is implemented), PET scan using 18F-FDG probe is optional at the discretion of the investigator.
6) The samples (blood and urine) for pharmacokinetic/exploratory markers evaluation will be collected.
   - ➢ Day 15 of Cycle 1: Collection of blood samples for pharmacokinetic evaluation at the time points prior to and 2 hours after IP administration.
   - ➢ Day 1 of Cycle 2: The patients may be hospitalized a day before the initiation of Cycle 2 (Day 28 of Cycle 1) to collect blood and urine samples for pharmacokinetic analysis. For the time points of blood/urine collection, refer to the Section 8. C2D1 should occur on Day 29.
   - ➢ A plasma sample for PK analysis will be obtained prior to and at 4 hours (the approximate T_{max}) after the IP administration, on Day 1 of Cycle 13 or later. This sample will be obtained at only one study visit; it will not be repeated at each study visit.

7) Concomitant medications/therapies will be checked.
8) AEs and toxicities will be checked.
9) The IP will be prescribed. IM156 may be administered either with food or in a fasted state at the discretion of the patient and the investigators. IM156 will be administered with water and swallowed whole without chewing.
10) At the time of initiating a cycle, compliance will be checked by counting the number of tablets which have not been taken in the prior cycle. At or after Cycle 13, compliance will be checked on Day 1 of every other cycle.
11) The next visit should be scheduled.

9.2.6. End of Treatment (EOT) Visit / Early Discontinuation (DC)

The patients who have discontinued the IP or who have been permanently withdrawn from this study are required to attend an EOT/DC visit at the study site within 7 days from the last administration of the IP. The following procedures will be carried out at this visit.

1) Vital signs (systolic and diastolic blood pressure, pulse rate, and body temperature) and weight/BSA will be measured and physical examination will be performed.
2) ECOG Performance Status will be evaluated.

3) 12-lead ECG will be performed.
4) Laboratory tests (hematology, blood chemistry, and tumor markers) will be conducted.
5) Tumor assessment and scans for exploratory markers evaluation (PET scan using ^{18}F-FDG probe is mandatory, Sestamibi scan and PET scan using ^{11}C-acetate probe are optional at the discretion of the investigator) will be conducted (If the tumor assessment has been made within 28 days from EOT, the result can be used for the EOT tumor assessment requirement.).
6) For exploratory markers evaluation, samples will be collected through biopsy, if possible (this can be performed at the Safety F/U visit).
7) Concomitant medications/therapies will be checked.
8) AEs will be checked.
9) At the time of initiating a cycle, compliance will be checked by counting the number of tablets which have not been taken in the prior cycle.
10) Next visit should be scheduled.

9.2.7. Safety Follow Up Visit after the End of Treatment (28 days after the last dose of IM156)

The safety follow up visit will be conducted 28 days after the last dose of IM156. At this visit, the following procedures will be carried out. This visit may be conducted via telephone call if the patient is not able to come to the study site.

1) Concomitant medications/therapies will be recorded.
2) AEs will be recorded.
3) Other anti-tumor therapy after EOT (if possible). The Safety Visit Follow up should be conducted prior to the start of any other ani-tumor therapy.

9.2.8. Unscheduled Visit

If a patient makes an unscheduled visit due to the necessity for medical treatment, the pertinent details should be recorded in a corresponding form. However, the scheduled visit should not be changed due to the unscheduled visit.

10. EXPECTED SIDE EFFECTS AND PRECAUTIONS ON USE

Refer to the Appendix 1. Precautions in Use of IM156

11. CRITERIA FOR DISCONTINUATION AND WITHDRAWAL

11.1. Criteria for Withdrawal of Patients

The study completion status of all the patients participating in the clinical study should be recorded, and any reason of discontinuation of drug administration and observation should be recorded. If any of the following conditions are applicable, the patients may be discontinued from the treatment or withdrawn from the study.

1) Progressive disease (PD)
2) Dose omission exceeds 4 weeks due to IP-related toxicities
3) Patient’s voluntary withdrawal of consent
4) Patients have received other treatments or therapies during the study period without approval of the responsible physician that may affect the study results
5) Patients do not comply with investigator’s directions
6) Patients who did not meet the inclusion/exclusion criteria
7) Patients who cannot be continued in the clinical study due to unmanageable toxicities, including patients that withdraw their consent due to an adverse event, regardless of grade or severity
8) Patients who are considered to be unable to participate in the clinical study due to a change in safety conditions and ethical perspective in the investigator’s judgment
9) Confirmation of pregnancy during the administration of IP
10) Patients are lost to follow up
11) Any other case in which investigators determine the study should be discontinued

Safety follow up (Safety F/U) should be carried out for the patients who are discontinued from the treatment due to the progressive disease, toxicities and other reason listed above. The safety F/U visit will be scheduled after the procedures for the EOT are completed. The Safety F/U visit should be conducted unless the discontinued patients clearly decline the Safety F/U visit. The EOT visit is not considered as the completion of the clinical study.

11.2. Management of Protocol Violation

The principal investigator and subinvestigators of this clinical study should be thoroughly familiar with and follow the protocol to ensure that protocol violations do not occur. To comply with the schedules for administration of the IP and tests in this clinical study, the investigators (or their designees) should take proper measures, for example, written notice or notification by telephone for the next visit schedule, to ensure the patients can make a visit on the applicable day without exception. However, any protocol violation that has arisen should be managed as follows.

In the case of serious protocol violations, the patient should be excluded from the PPS analysis, and this will be confirmed at the analysis set meeting.

In addition, minor protocol violations are considered to have no influence on the interpretation of the study results. The degree of violations or delays and the reasons should be documented accurately and whether they have affected the study should be reviewed comprehensively by the investigators, sponsor, monitors, and statisticians when the clinical study report is being prepared.

11.3. Criteria for Study Discontinuation

If it is considered to be unethical to continue the clinical study in light of the outcomes observed during the clinical study, the investigators can discontinue the study partially or entirely in consultation with the sponsor. In addition, the sponsor can discontinue the clinical study partially or entirely for safety or administrative reasons.

If the sponsor requests an early termination or temporaly suspension of the clinical study, the investigators should notify this fact to the institutional review board (IRB) immediately and submit a detailed statement of reasons for the early termination and/or temporary suspension to the IRB.

If the clinical study has been terminated early or suspended temporarily, the investigators should inform the patients of this fact immediately and make proper measures and implement follow-up procedures.

At study discontinuation, the principal investigator should deliver the following documents to the sponsor: the electronic case report forms of the patients, all progress status and outcomes of the clinical study. The principal investigator should follow the destruction of IP procedure provided by the sponsor.

12. CRITERIA AND METHOD FOR EFFICACY EVALUATION

12.1. Evaluation based on tumor assessment

Tumor response will be evaluated by presenting the response rate and the control rate based on the best overall response. The best overall response means the best response among the confirmed assessments of tumor responses and the definitions of assessment variable are as follows.

1) ORR

: Proportion of patients with CR or PR

2) DCR

: Proportion of patients with complete response (CR), partial response (PR) or stable disease (SD)

3) DoR

: Time from documentation of tumor response (CR or PR) to disease progression (PD)

Complete and partial responses should be re-confirmed by a second assessment at least 4 weeks from the day the first response was observed. Stable disease is assessed if it is observed at least once at a minimum of 6 weeks after the initiation of treatment.

Tumor response will be determined based on RECIST v1.1 (Appendix 2), RANO criteria for rGBM (Appendix 3), or RECIL (2017) criteria for lymphoma.

12.2. Evaluation on Response and Survival Period

1) PFS

: Time from the first day of study drug administrationf until PD or death

13. CRITERIA AND METHOD FOR SAFETY EVALUATION INCLUDING ADVERSE EVENTS

13.1. Criteria and Method for Safety Evaluation

Safety evaluation will be conducted by monitoring of patientive and objective AEs, measuring vital signs, and performing physical examination, laboratory tests (hematology/blood chemistry/coagulation tests/urinalysis), and 12-lead ECG test, etc.

13.1.1. Adverse Events

AEs are general terms for symptoms, signs, and abnormal laboratory test values that have occurred or have deteriorated after IP administration. Types, occurrence and end dates, severity, and treatments and outcome of AEs, and their causal relationship with the IP, etc. should be recorded in the AEs section of a case report form. If those are not recorded, those will be considered as subjective symptoms. All AEs will be coded by the MedDRA v19.0 (or an upper version) and classified by SOC and PT.

13.1.2. Vital Signs, Physical Examination, Laboratory Tests, and 12-lead ECG

Measurement, test methods, and schedules of vital signs, etc. for safety evaluation will be carried out according to Sections 9.1.5~9.1.11, and any finding that corresponds to the definition of an AE in vital signs, physical examination, laboratory tests, and 12-lead ECG test should be reported as an AE.

13.2. Evaluation Criteria and Reporting Method for Adverse Events

13.2.1. Definition of Terms

1) Adverse Event (AE)

AEs are defined as any unfavorable and unintended signs (including abnormal laboratory findings, etc.), symptoms or diseases that occur in a patient who received an IP and does not necessarily have to have a causal relationship with the IP.

2) Adverse Drug Reaction (ADR)

ADRs include cases in which harmful and unintended reactions have occurred at any dose of the IP and their causal relationship with the IP cannot be ruled out.

3) Serious AE·ADR

SAEs include cases in which AEs or adverse drug reactions that have occurred at any dose of the IP are applicable to any of the following.

- A case that results in death or is life-threatening
- A case that requires hospitalization or prolongation of existing hospitalization[1)]
- A case that results in permanent or significant disability and reduced capacity
- A case that results in congenital anomaly or birth defect in fetus

[1)] The following cases are not applicable to 'the case that requires hospitalization or prolongation of existing hospitalization' and should not be reported as AEs;

- Hospitalization or prolongation of existing hospitalization for a procedure (e.g., operation, examination) that had been scheduled prior to the study

- Hospitalization or prolongation of existing hospitalization for follow-up observation of an already healed or improved condition
- Hospitalization or prolongation of existing hospitalization for examination or education
- Hospitalization or prolongation of existing hospitalization for non-medical reasons (e.g., temporary absence of a family member)
- Admission to a hospice, or nursing care, or rehabilitation facility
- Admission due to disease progression

If any situation considered to have a medically serious effect on the safety and health condition of a patient has occurred even though it is not included in the situations listed above, the responsible physician and relevant health professionals will decide whether or not it should be considered as a SAE based on their medical judgment and take proper measures.

Progressive disease determined through tumor assessment will not be reported as an AE.

13.2.2. Criteria for Severity Assessment

Severity of AEs will be classified according to the NCI-CTCAE v4.03 (NCI-CTCAE, 2010), and those which cannot be classified by the NCI-CTCAE will be evaluated by the following 5-grade criteria.

Table 13.2.1. Criteria for Severity Assessment

Grade	Description of Severity
1	Mild; Perceivable symptoms or signs but endured without difficulty
2	Moderate; Discomfort that is enough to restrict normal daily lives
3	Severe; Discomfort that is enough to make normal daily lives infeasible.
4	Life-threatening
5	Death

13.2.3. Assessment Criteria for Causality with Investigational Product

When AEs occur, the causality with IP is classified and evaluated by the investigator as follows;

1) Certain
   - If evidence of the IP administration exists and the temporal relationship between administration of the IPand occurrence of the AE is plausible
   - If the AE can be explained by the administration of the IP with higher probability than any other reasons
   - If the AE disappears by discontinuing IP administration
   - If the outcome of re-challenge (only if feasible) is positive
   - If the AE shows a pattern consistent with the information already known about the IP or products of the same class
2) Probable, Likely
   - If evidence of the IP administration exists and the temporal relationship between administration of the IP and occurrence of the AE is plausible
   - If the AE can be explained by the administration of the IP with higher probability than any other reasons

- If the AE disappears by discontinuing the administration

3) Possible
   - If evidence of the IP administration exists and the temporal relationship between administration of the IP and occurrence of the AE is plausible
   - If the AE is considered to have been caused by the IP administration with the same level of probability as the other possible reasons
   - Information on IP withdrawal may be lacking or unclear
4) Unlikely
   - If evidence of the IP administration exists, and the relationship between administration of the IP and occurrence of the AE is improbable (but not impossible)
   - Disease or other drugs provide plausible explanations
5) Conditional/Unclassified
   - More data for proper assessment needed, or additional data under examination
6) Unassessible/Unclassifiable
   - Data cannot be judged because information is insufficient or contradictory, and cannot be supplemented or verified.
7) Not applicable
   - No drug intake

13.2.4. Reporting Methods

13.2.4.1. Reporting of adverse events

The investigators must inform patients (or their representatives) of all the AEs that may possibly occur after IP administration and instruct them to report all the symptoms that may occur.

The investigators must record the details of all symptoms identified through physical examination or laboratory tests including their types, occurrence and end dates, severity, treatments and outcomes, and causal relationship with the IP on the appropriate case report form. In addition, the pertinent AEs should be followed up until they are resolved (disappearance of AEs or lost contact to follow up, etc.).

13.2.4.2. Immediate reporting

For all SAEs that occurr while the IP is being administered or within 28 days after administration is terminated, the investigator should report them to the IRB as well as the sponsor (or its designee) by e-mail or fax. SAEs must be reported immediately after or within 24 hours after learning of them regardless of their causality with the IP. At the time of initial reporting, all the details in the SAE Form should be included as possible, and afterward, the completed form should be sent to the sponsor. These should also be recorded in the AEs section of a case report form.
The sponsor (or its designee) and the IRB should review the information after receiving the initial report and contact the principal investigator to obtain more detailed information, if necessary. For unexpected serious adverse drug reactions, they should be reported to the applicable local regulatory authority within the following established time limit.

- If the event leads to death or is life threatening: Within 7 days of when the client receives a report or becomes aware of the relevant facts. The client must submit an additional report that includes details on the relevant adverse drug event within 8 days of receiving the first report or becoming aware of the facts of the adverse drug event.

- Any other unexpected serious adverse drug reaction: Within 15 days from the date when the sponsor received the report or learned of the pertinent facts.

In addition, the sponsor should follow up pertinent adverse drug reactions until they are resolved (disappearance of adverse drug reaction or lost to follow up, etc.), in case it obtains any additional information on the adverse drug reactions reported in compliance with the above.

Contact information for reporting of AEs is as follows;

- Name: LSK Global PS, PV department
- E-mail: lsk.pv@lskglobal.com
- Fax number: 82-2-6919-2472

13.2.5. Actions to be taken for Serious Adverse Events

If any SAE occurs during this clinical study, the investigators should report it to the sponsor or its designee and the IRB according to the procedure of the Section 13.2.4.2. In addition, the AE should be minimized with prompt and appropriate measures taken for the patients. The IRB can order necessary measures to be taken after reviewing the severity and causality of the SAE. The sponsor should review the SAE report received from the investigator and submit it to local regulatory authorities, if necessary. In addition, the sponsor should inform the IRBs and the principal investigators of the other study sites of this SAE.

SAEs including those occurring within 28 days (even though other chemotherapies are initiated) after the last administration of the IP should be followed up and reported. However, scheduled hospitalization to treat the disease which existed prior to participation in the clinical study or to receive other chemotherapies will not be reported as a SAE. The investigators should monitor the patients until confirmation that all SAEs are resolved or resolved with sequelae.

13.2.6. Progression of Underlying Diseases

Progressive disease (PD) may be considered as the deterioration of condition due to an underlying cancer of the pertinent patient. Obvious PD (new metastasis of the primary cancer or progression of the existing metastasis, etc.), and symptoms and signs caused by the existing cancer will not be reported as an AE or SAE (including death obviously caused by the PD). However, progressive clinical symptoms where it can’t be determined whether they have been caused by progression of the existing malignant disorder or they are not in accordance with the expected progression pattern of the target disorder of the clinical study should be reported as AEs and SAEs. Although death obviously caused by the progressive disease should not be reported as a SAE, information on the death (date of death, cause of death, etc.) should be recorded in the case report form.

14. STATISTICAL ANALYSIS METHOD

This study is an open-label clinical study to determine the recommended dose of the IP, to confirm the safety, tolerability, and PK characteristics, and to evaluate exploratory markers and anti-tumor activity. No hypothesis testing of the IP compared to the control drug will be carried out in this clinical study. Thus, descriptive statistics will be presented for the study variables. In general, descriptive statistics will be summarized by dose group for the Phase 1a study and by disease group for the Phase 1b study. For continuous variables, mean ± standard deviation (SD), median, minimum, and maximum will be presented and for categorical variables, frequency and percentage (%) will be presented.

If there is any missing data at a time point, time to event data will be censored. For the variables other than time to event, data will not be imputed.

14.1. Definition of Analysis Groups

In this clinical study, the FAS (Full Analysis Set) will be analyzed at the time of assessing tumor response in the Phase 1a study. For evaluating efficacy in the Phase 1b study, the FAS will be used as the main analysis population and the PP (Per-Protocol Set) will be used for the supplementary analysis. Safety evaluation will be carried out in the Safety Set.

1. FAS (Full Analysis Set):

The FAS includes patients who have been administered the IP at least once and have been evaluated for tumor response according to RECIST ver. 1.1 (RANO for rGBM).

2. PPS (Per Protocol Set):

The PPS includes patients who are in the FAS and have no serious protocol violations as follows.

8) Inclusion/exclusion criteria have been seriously violated
9) Prohibited concomitant medications have been administered
10) Other serious violation of the protocol

3. Safety Set:

Safety analyses will be carried out in the patients who have been administered the IP and evaluated for safety at least once. The patients who have been enrolled in the clinical study but dropped out prior to administration of the IP will be excluded from the Safety Set.

14.2. Baseline Demographic Data and Medical/Medication Histories

Descriptive statistics on the demographic information (age, gender, etc.) and baseline characteristics of patients prior to treatment will be presented. For continuous variables, number of patients, mean ± SD, median (range), minimum, and maximum will be presented, and for categorical variables, frequency and percentage (%) will be presented.

14.3. Efficacy Endpoints

- ORR:
  Proportions of patients evaluated as CR or PR for the best overall response should be presented as the frequency and the percentage of patients. They will be summarized by dose group for the Phase 1a study and by disease group for the Phase 1b study, and the two-sided 95% CI will also be provided.
- DCR:
  Proportions of patients evaluated as CR, PR, or SD for the best overall response should be

presented as the frequency and the percentage of patients. For the Phase 1a study, they will be summarized by dose group, and the two-sided 95% CI will also be provided for the Phase 1b.

- Survival data – DoR, PFS, OS:
  For survival data, Kaplan-Meier curve will be estimated and the median value for each parameter (median progression free survival) will be summarized. For the results of the Phase 1b, they will be summarized by disease group and the two-sided 95% CI will also be presented. For the Phase 1a, they will be summarized by in total and by dose group.
  The rules on the censoring date of the PFS and the reference date of the time point of disease progression should comply with Table 14.3.1.

Table 14.3.1. PFS Censoring Rules (including only the disease progression documented by imaging data)

Situation	Date of Progression or Censoring	Outcome
No baseline tumor assessments	Date of the last visit to the site	Censored
Progression documented between scheduled visits	Whichever comes first:<br>• Date of tumor assessment indicating new lesion (if progression is based on new lesion); or<br>• Date of last tumor assessment measuring lesions (if progression is based on increase in sum of measured lesions)	Progressed
No progression	Date of last tumor assessment of measured lesions	Censored
Treatment discontinuation for undocumented progression	Date of last tumor assessment of measured lesions	Censored
Treatment discontinuation for toxicity or other reason	Date of last tumor assessment of measured lesions	Censored
New anticancer treatment started	Date of last tumor assessment of measured lesions	Censored
Death before first PD assessment	Date of death	Censored
Death between adequate assessment visits	Date of death	Censored
Death or progression after more than one missed visit	Date of the last visit to the site	Censored

14.4. Safety Endpoints

Safety analyses will be carried out in the Safety Set based on AEs, abnormalities in outcomes of laboratory tests and physical examination, etc.

For safety evaluation, descriptive statistics on the DLTs, AEs, laboratory tests, physical examination, 12-lead ECG, etc. will be presented according to the form of data. For the Phase 1a study, the incidence and number of events will be presented for the DLTs observed in Cycle 1 by dose group. For treatment emergent adverse events (TEAEs mean the AEs which did not exist prior to administration of the IP but occurred after administration, or AEs which were exacerbated after administration of the IP although they existed prior to administration) of the IP, the numbers

and incidences of AEs and ADRs will be presented by dose group for the Phase 1a study and for all subjects in the Phase 1b study, with two-sided 95% CIs. AEs and ADRs will be standardized with the SOC (System Organ Class) and the PT (Preferred Term) by using the MedDRA. In addition, SAEs, severity, AEs leading to withdrawal, and TEAEs will be presented by dose group in the Phase 1a study and by disease group for the Phase 1b study. Besides, descriptive statistics on laboratory test values, 12-lead ECG, etc. will be presented by visit.

14.5. Pharmacokinetic Parameters

The PK parameters to be evaluated are plasma AUC_{inf}, $AUC_{0–last}$, C_{max}, C_{min}, T_{max}, $T_{1/2}$, CL/F, accumulation ratio at steady state, Ae, CLr, etc., and will be described by using descriptive statistics according to the form of data. The report on PK evaluation may be presented separately from the clinical study report.

14.6. Exploratory Marker Parameters

The exploratory marker parameters will be described according to the form of data by using descriptive statistics. Correlation between the dose of IM156 and AUC, etc. will be analyzed, if necessary. The report on exploratory marker evaluation may be presented separately from the clinical study report.

.

15. ETHICS

This clinical study will be conducted ethically and scientifically in compliance with KGCP and all the relevant regulations. In addition, this clinical study will be conducted based upon the Declaration of Helsinki, ensuring it will not cause any disadvantage to patients as well as the dignity, rights and interests of patients will be respected.

15.1. Institutional Review Board (IRB)

Before initiating the clinical study, the principal investigator is responsible for obtaining a written approval from the IRB for the protocol, patient information sheet and informed consent form, data and procedures relevant with the recruitment of patients, etc. The IRB of each study site will review the ethical, scientific, and medical validity of the clinical study and provide its decision in writing to the principal investigator and the sponsor prior to initiation of the study.

15.2. Patient Informed Consent

The investigators should provide the patients (or their representatives) with sufficient explanation about the contents, procedures, effects, and potential AEs of the study in advance, and obtain patient informed consent in writing. The investigators should provide the patients (or their representatives) with one copy of the signed consent form and patient information sheet, and the original copy of the consent form should be kept in the investigator file.

Refer to Attachment 3. Patient information sheet and informed consent form.

15.3. Confidentiality

Confidentiality of any record that can verify the identity of the patient should be maintained. All the documents relevant with the clinical study, CRF, etc. will be recorded and classified not by using a patient name but by using a patient identification code. Even in case the study results are published, confidentiality of the patient’s identity will be maintained.

The sponsor, or clinical research associates, auditors, and any regulatory authorities and the IRB may have access to the medical records of the patients to verify the collected data, and confidentiality of patients’ information should be strictly maintained even during the audit.

15.4. Safety Measures for Protection of Patients

15.4.1. Measures to be taken for Adverse Events

If AEs have occurred due to this clinical study, the investigators should take measures to ensure the patient can have necessary examinations and treatments immediately. In addition, the investigators should follow up the pertinent AEs until the patient is recovered or lost to follow up, if necessary.

15.4.2. Medical Treatment and Therapy for Patients after Study

The patients who have withdrawn from the clinical study or are unresponsive to the treatment should be informed that they are able to receive other treatments. For those patients who complete the clinical study, they are allowed to have medical treatment for the unexpected delayed AEs by the physician’s direction at any time.

15.4.3. Regulation of Compensation for Damage to the Health of Patients

Refer to Attachment 4. Regulation of compensation for damage to the health of patients

16. OTHER REQUIREMENTS FOR SAFE AND SCIENTIFIC CONDUCT OF STUDY

16.1. Management and Retention of Records

The principal investigator should retain the various data (including electronic documents) relevant for the clinical study including the protocol, source documents, records on the IP, etc. under a proper storage condition for 3 years (or the approval date of document) (however, the retention period may be extended, if the sponsor considers it is required).

These documents will be subject to investigation at the time of inspection by the sponsor or relevant regulatory authorities, and the investigators should not destroy any document relevant to the clinical study without written approval of the sponsor. The investigators should take preventive measures against any accident of these documents.

16.1.1. Source Documents

Source documents are defined as the output of the activities for data collection and observation in the clinical study. The source documents include medical records, electronic data, and measurement recordings by equipment, etc. and other records can be included in them. All the source documents that exist for this clinical study are recorded and kept by the investigators of the study sites and only authorized persons can have access to and peruse the source documents.

16.1.2. Collection of Study Data

The Electronic Data Capture (EDC) system used in this clinical study follows the regulations of 21 CFR part 11 (Code of Federal Regulations). The EDC system can be accessed only when authorized, and all the traces of entry, correction, storage, and deletion in the eCRF will be recorded. The investigators should assure that the data of eCRFs are accurate, complete, legible, and timely by placing their electronic signatures in them.

After the study is terminated, eCRFs prepared through the EDC system will be delivered to the study site after patients' data are saved in an electronic storage device, and will be stored on the same criteria as the other source documents.

16.2. Quality Control and Quality Assurance

16.2.1. Monitoring of Study Site

Monitoring will be carried out to confirm patients' rights and welfare are protected, to confirm that the reported study-related data are accurate, complete, and verifiable in comparison with the source documents, and to confirm that the study is conducted in compliance with the approved protocol and relevant rules and regulations such as the KGCP, etc.

Monitoring of the clinical study will be conducted through the regular visits and communications of the sponsor (or its designee) and/or monitors. Their visit schedules will be properly distributed after consultation between investigators and monitors.

The monitors should check the source documents, records on the management of the IP, storage condition of documents, etc., and review the overall process of the clinical study. In addition, the

monitors should discuss the discovered problems with the investigators and the investigators should cooperate in this process.

16.2.2. Audit

The sponsor may conduct audits separately from routine monitoring during the study for Quality Assurance. Audits include verification of whether the clinical study has been being conducted in compliance with the protocol, standard operating procedure, and relevant rules and regulations such as the KGCP, etc. and reviews of all the source documents, medication records, medical records, and so on. The sponsor (or its designee) may request for access to source documents and other essential documents, and the investigators should allow this and cooperate in this process.

16.2.3. Inspection

Regulatory authorities may conduct inspections during or after the study, and once an inspection has been scheduled, the investigator should inform the sponsor of this fact immediately. The local regulatory authorities can request for access to source documents and other essential documents to conduct an inspection of the study site and the investigators should allow this and cooperate in this process.

A separate storage place should be prepared to preserve the various data and records relevant with the conduct of the clinical study, and the security of the storage place should be maintained.

16.3. Amendment to the Protocol

If the study protocol has to be amended, approval for the amended protocol should be obtained from the IRB, and local regulatory authorities, if necessary.

17. References

Birsoy, K. et al., 2014. Metabolic determinants of cancer cell sensitivity to glucose limitation and biguanides. *Nature,* 508(7494), pp. 108-112.

Buzzai, M. et al., 2007. Systemic treatment with the antidiabetic drug metformin selectively impairs p53-deficient tumor cell growth. *Cancer Research,* 67(14), pp. 6745-6752.

Chan, M. M., Sjoquist, K. M. & Zalcberg, J. R., 2015. Clinical utility of ramucirumab in advanced gastric cancer. *Biologics,* 22(9), pp. 93-105.

DeCensi, A. et al., 2010. Metformin and Cancer Risk in Diabetic Patients: A Systematic Review and Meta-analysis. *Cancer Prevention Research,* 3(11), pp. 1451-1461.

Evans, J. M. M. et al., 2005. Metformin and reduced risk of cancer in diabetic patients. *BMJ,* Volume 330, pp. 1304-5.

Evans, J. M. M. et al., 2005. Metformin and reduced risk of cancer in diabetic patients. *theBMJ,* 330(7503), pp. 1304-1305.

FDA, 2009. *Nonclinical evaluation for anticancer pharmaceuticals, S9,* s.l.: s.n.

Gopal, Y. V. et al., 2014. Inhibition of mTORC1/2 overcomes resistance to MAPK pathway inhibitors mediated by PGC1α and oxidative phosphorylation in melanoma. *Cancer Research,* 74(23), pp. 7037-7047.

Haq, R. et al., 2014. Oncogenic BRAF regulates oxidative metabolism via PGC1α and MITF. *Cancer Cell,* 23(3), p. 302–315.

Hirsch, H. A., Iliopoulos, D., Tsichlis, P. N. & Struhl, K., 2009. Metformin selectively targets cancer stem cells, and acts together with chemotherapy to block tumor growth and prolong remission. *Cancer Research,* 69(19), pp. 7507-7511.

ImmunoMet, 2018. *Investigator's Brochure IM156.,* s.l.: s.n.

Jeon, S.-M., 2016. Regulation and function of AMPK in physiology and diseases. *Experimental & Molecular Medicine,* 48(7), p. e245.

Li, C. et al., 2009. Copy number abnormalities, MYC activity, and the genetic fingerprint of normal B cells mechanistically define the microRNA profile of diffuse large B-cell lymphoma. *Blood,* 113(26), pp. 6681-6690.

Li, Y. et al., 2014. MYC through miR-17-92 suppresses specific target genes to maintain survival, autonomous proliferation, and a neoplastic state. *Cancer Cell,* 26(2), pp. 262-272.

NCI-CTCAE, 2010. *Common Terminology Criteria for Adverse Events (CTCAE) version 4.03,* s.l.: U.S.DEPARTMENT OF HEALTH AND HUMAN SERVICES.

NITR, 2005. *Guideline on Food-Effect Bioavailability and Fed Bioequivalence Studies,* s.l.: National Institute of Toxicological Research.

Pastò, A. et al., 2014. Cancer stem cells from epithelial ovarian cancer patients privilege oxidative phosphorylation, and resist glucose deprivation. *Oncotarget,* 30(5), pp. 4305-4319.

Roesch, A. et al., 2013. Overcoming intrinsic multidrug resistance in melanoma by blocking the mitochondrial respiratory chain of slow-cycling JARID1B(high) cells. *Cancer Cell,* 23(6), pp. 811-825.

Sancho, P. et al., 2015. MYC/PGC-1α Balance Determines the Metabolic Phenotype and Plasticity of Pancreatic Cancer Stem Cells. *Cell Metabolism,* 22(4), pp. 590-605.

Savage, K. J. et al., 2009. MYC gene rearrangements are associated with a poor prognosis in diffuse large B-cell lymphoma patients treated with R-CHOP chemotherapy. *Blood,* 114(17), pp. 3533-3537.

Sehn, L. H. et al., 2007. The revised International Prognostic Index (R-IPI) is a better predictor of outcome than the standard IPI for patients with diffuse large B-cell lymphoma treated with R-CHOP. *Blood,* 109(5), pp. 1857-1861.

Siegel, R. L., Miller, K. D. & Jemal, A., 2017. Cancer Statistics, 2017. *CA: A Cancer Journal for Clinicians,* 67(1), pp. 7-30.

Sotgia, F., Fiorillo, M. & Lisanti, M. P., 2017. Mitochondrial markers predict recurrence, metastasis

and tamoxifen-resistance in breast cancer patients: Early detection of treatment failure with companion diagnostics. *Oncotarget,* 8(40), pp. 68730-68745.
Sotgia, F. & Lisanti, M. P., 2017. Mitochondrial markers predict survival and progression in non-small cell lung cancer (NSCLC) patients: Use as companion diagnostics. *Oncotarget,* 8(40), pp. 68095-68107.
Thieblemont, C. & Brière, J., 2013. MYC, BCL2, BCL6 in DLBCL: impact for clinics in the future. *Blood,* 121(12), pp. 2165-2166.
Vayalil, P. K. & Landar, A., 2015. Mitochondrial oncobioenergetic index: A potential biomarker to predict progression from indolent to aggressive prostate cancer. *Oncotarget,* 6(40), pp. 43065-43080.
Vellinga, T. T. et al., 2015. SIRT1/PGC1α-Dependent Increase in Oxidative Phosphorylation Supports Chemotherapy Resistance of Colon Cancer. *Clinical Cancer Research,* 15(21), pp. 2870-2879.
Viale, A. et al., 2014. Oncogene ablation-resistant pancreatic cancer cells depend on mitochondrial function. *Nature,* 514(7524), pp. 628-632.
Wheaton, W. W. et al., 2014. Metformin inhibits mitochondrial complex I of cancer cells to reduce tumorigenesis. *Elife,* Volume 3, p. e02242.
Yuan, P. et al., 2013. Phenformin enhances the therapeutic benefit of BRAFV600E inhibition in melanoma. *PNAS,* 110(45), pp. 18226-18231.
Zhang, Z.-J. & Li, S., 2014. The prognostic value of metformin for cancer patients with concurrent diabetes: a systematic review and meta-analysis. *Diabetes, Obesity and Metabolism,* 16(8), pp. 707-710.

18. APPENDICES

Appendix 1. Precautions in use of IM156

1. Adverse events

Since this medication has never been administered to humans, the following can be anticipated based upon the adverse events of metformin, a medication of the same biguanide class.

4) Lactic acidosis: Although increase in lactic acid has not been caused by repeated administration of this drug and this drug is considered to be comparatively safe from the influence of lactic acid, lactic acidosis may rarely occur depending on the degree of renal insufficiency and patient's age. Once this occurs, it is life-threatening. Initial symptoms of lactic acidosis are sometimes indistinguishable, and nonspecific symptoms such as malaise, myalgia, dyspnea, increasing somnolence, and abdominal pain are accompanied. If acidosis gets more severe, hypothermia, hypotension, and refractory bradyarrhythmia may occur. If these symptoms appear, patients should notify this to physicians immediately.
5) Digestive system: When this medication is administered, gastrointestinal symptoms (diarrhea, nausea, abdominal distension, anorexia, dyspepsia, constipation, abdominal pain) may occur.
6) Sensory system: Although metallic taste may occur, it generally disappears gradually.
7) Skin: Since erythema, pruritus, urticaria, rash, etc. may occur, administration of this medication should be discontinued in such cases.
8) Blood system: Anemia, leucopenia, and thrombocytopenia may rarely occur.
9) Liver: Liver dysfunction may sometimes occur.
10) Hypoglycemia: Although hypoglycemia does not occur in patients receiving biguanide drug alone and no influences of this drug on blood glucose were observed, hypoglycemia may occur at the time of combination with insulin or oral antidiabetics that may induce hypoglycemia.

2. General precautions

11) Monitoring of renal function: The route of excretion of this medication has not been studied yet. The risk of accumulation of biguanide drugs and lactic acidosis generally increases with the degree of renal impairment. Thus, this medication should not be administered to the patients with serum creatinine value above the upper limit of normal (ULN) for their age. In patients with elderly, this medication should be carefully titrated to establish the minimum dose for adequate glycemic control effect, since aging is associated with reduced renal function. In elderly patients, renal function should be monitored regularly and generally, this medication should not be titrated to the maximum dose.
12) Hypoxic states: Cardiovascular collapse (shock) from whatever cause, acute congestive heart failure, acute myocardial infarction and other conditions characterized by hypoxemia have been associated with lactic acidosis and may also cause prerenal azotemia. When such events occur in patients on this medication therapy, the drug should be promptly discontinued.
13) Alcohol intake: Alcohol is known to potentiate the effect of this medication on lactate metabolism and increase the risk of lactic acidosis. Therefore, patients should be warned against acute or chronic excessive alcohol intake, while receiving this medication.
14) Patients of specific occupations: Since this medication may rarely cause serious lactic acidosis and delayed severe hypoglycemia, caution should be exercised when this medication is administered to patients who engage in high place work, driving, etc. In addition, patients and their families should be sufficiently and thoroughly reminded of the precautions against lactic acidosis and hypoglycemia.

15) Risks of lactic acidosis, its symptoms and conditions that predispose to its development should be explained to patients. Patients should be advised to discontinue this medication immediately and to promptly notify their health practitioner if unexplained hyperventilation, myalgia, fatigue, unusual somnolence, or other nonspecific symptoms occur. Patients should be counselled against the risk of alcohol intake, either acute or chronic, while receiving this medication.

Appendix 2. Response Evaluation Criteria in Solid Tumors (RECIST) version 1.1

Reference: Eisenhauer EA, et al. New response evaluation criteria in solid tumors: Revised RECIST guideline (version 1.1)

1. Measurability of tumor at baseline

1.1 Definitions

At baseline, tumor lesions/lymph nodes will be categorized measurable or non-measurable as follows:

1.1.1 Measurable tumor lesions

Tumor lesions must be accurately measured in at least one dimension (longest diameter in the plane of measurement is to be recorded) with a minimum size as follows:

10 mm by CT or MRI scan (CT/MRI scan slice thickness no greater than 5 mm)

10 mm caliper measurement by clinical examination (lesions which cannot be accurately measured with calipers should be recorded as non-measurable)

20 mm by chest X-ray

Malignant lymph nodes: To be considered pathologically enlarged and measurable, a lymph node must be ≥15 mm in short axis when assessed by CT scan (CT scan slice thickness recommended to be no greater than 5 mm). At baseline and in follow-up, only the short axis will be measured and followed. See the clause “2.2. Baseline documentation of target and non-target lesions”, for information on lymph node measurement.

1.1.2 Non-measurable tumor lesions

Non-measurable tumor lesions include small lesions (longest diameter <10 mm or pathological lymph nodes with ≤10 to <15 mm short axis) as well as truly non-measurable lesions. Lesions considered truly non-measurable include: lepto-meningeal disease, ascites, pleural or pericardial effusion, inflammatory breast disease, lymphangitic involvement of skin or lung (lymphangitis cutis/pulmonis), peritoneal transmission, abdominal masses/abdominal organomegaly identified by physical exam that is not measurable by reproducible imaging techniques

1.2 Target lesions

1.2.1 Measurement of lesions

All measurements should be recorded in metric notation, using calipers if clinically assessed. All baseline evaluations should be performed as close as possible to the initiation of administration and never more than 4 weeks before the beginning of the treatment.

1.2.2 Method of assessment

The same method of assessment and the same technique should be used to characterize each identified and reported lesion at baseline and during the clinical study. Imaging based evaluation should always be preferred.

Clinical lesions: Clinical lesions will only be considered measurable when they are superficial

and ≥10 mm diameter as assessed using calipers (e.g. skin nodules). For the case of skin lesions, documentation by color photography including a ruler to estimate the size of the lesion is suggested.

Chest X-ray: Chest CT is preferred over chest X-ray (particularly when progression is an important endpoint), since CT is more sensitive than X-ray (particularly in identifying new lesions). However, lesions on chest X-ray may be considered measurable if they are clearly defined and surrounded by aerated lung.

CT, MRI: CT is the best currently available and reproducible method to measure lesions selected for response assessment. This guideline has defined measurability of lesions on CT scan based on the assumption that CT slice thickness is 5 mm or less. When CT scans have slice thickness > 5 mm, the minimum size for a measurable lesion should be twice the slice thickness. MRI is also acceptable.

If prior to enrolment it is known a patient is not able to undergo CT scans with IV contrast due to allergy or renal insufficiency, the decision as to whether a non-contrast CT or MRI (without IV contrast) should be used to evaluate the patient at baseline and during the study should be guided by the tumor type under investigation and the anatomic location of the disease. For patients who develop contraindications to contrast after baseline contrast CT is done, the decision as to whether non-contrast CT or MRI (enhanced or non-enhanced) should be performed should also be based on the tumor type and anatomic location of the disease. Each case should be discussed with the radiologist to determine if substitution of these other approaches is possible and, if not, the patient should be considered not evaluable from that point forward.

Ultrasound: Ultrasound is not useful in assessment of lesion size and should not be used as a method of measurement. If new lesions are identified by ultrasound, confirmation by CT or MRI is advised.

Endoscopy, laparoscopy, tumor markers: The utilization of these techniques for objective tumor evaluation is not advised in general.

Cytology, histology: These techniques can be used to differentiate between PR and CR in rare cases (for example, to differentiate between residual benign lesions and residual malignant lesions in tumor types such as germ cell tumors). When effusions are known to be a potential adverse event of treatment (e.g. with certain taxane compounds or angiogenesis inhibitors), the cytological confirmation of the neoplastic origin of any effusion that appears or worsens during treatment can be considered if the measurable tumor has met criteria for response or stable disease to differentiate between response (or SD) and PD.

2. Tumor response evaluation

2.1 Assessment of overall tumor burden and measurable disease

To assess objective response or future progression, it is necessary to estimate the overall tumor burden at baseline and use this as a comparator for subsequent measurements. Measurable disease is defined by the presence of at least one measurable lesion.

2.2 Baseline documentation of ‘target’ and ‘non-target’ lesions

When more than one measurable lesion is present at baseline, a maximum of two lesions per organ and all lesions up to a maximum of five lesions in total (representative of all involved organs) should be identified as target lesions and will be recorded and measured at baseline.

This means in instances where patients have only one or two organ sites involved, a maximum of two (one site) and four lesions (two sites) respectively will be recorded. Other lesions of each organ (even though they are measurable) will be recorded as non-measurable (even though their sizes are >10 mm at CT scans). Target lesions should be selected on the basis of their size (lesions with the longest diameter), be representative of all involved organs, but in addition should be those that lend themselves to reproducible repeated measurements. It may be the case that, on occasion, the largest lesion does not lend itself to reproducible measurement in which circumstance the next largest lesion which can be measured reproducibly should be selected.

Lymph nodes merit special mention since they are normal anatomical structures which may be visible by imaging even if not involved by tumor. Pathological nodes which are defined as measurable and may be identified as target lesions must meet the criterion of a short axis of ≥15 mm by CT scan. Only the short axis of these nodes will contribute to the baseline sum. The short axis of the node is the diameter normally used by radiologists to judge if a node is involved by solid tumor. Nodal size is normally reported as two dimensions in the plane in which the image is obtained. The smaller of these measures is the short axis. For example, an abdominal node which is reported as being 20 mm x 30 mm has a short axis of 20 mm and qualifies as a malignant, measurable node. In this example, 20 mm should be recorded as the node measurement. All other pathological nodes (those with short axis ≥10 mm but <15 mm) should be considered as non-target lesions. Nodes that have a short axis <10 mm are considered to be non-pathological and should not be recorded or followed.

A sum of the diameters (longest for non-nodal lesions, short axis for nodal lesions) for all target lesions will be calculated and reported as the baseline sum diameters. If lymph nodes are to be included in the sum, then as noted above, only the short axis is added into the sum. The baseline sum diameters will be used as reference to further characterize any objective tumor regression in the measurable dimension of the disease. All other lesions (or sites of disease) including pathological lymph nodes should be identified as non-target lesions and should also be recorded at baseline. Measurements are not required and these lesions should be followed as "present", "absent", or in rare cases "unequivocal progression". In addition, it is possible to record multiple non-target lesions involving the same organ as a single item on the case report form (e.g. "multiple enlarged pelvic lymph nodes" or "multiple liver metastases").

2.3 Response criteria

This section provides the definitions of the criteria used to determine objective tumor response for target lesions.

2.3.1 Evaluation of target lesions

Table 1. Response criteria for target lesions

Response of target lesions	Definition
Complete response (CR)	Disappearance of all target lesions. And, any nodal pathological target lesion must have reduction in short axis to

Response of target lesions	Definition
	<10 mm.
Partial Response (PR)	At least a 30% decrease in the sum of longest diameters of target lesions, taking as reference the baseline sum diameters.
Stable Disease (SD)	Neither sufficient shrinkage to qualify for PR nor sufficient increase to qualify for PD, taking as reference the smallest sum diameters while on study
Progressive Disease (PD)	At least a 20% increase in the sum of diameters of target lesions, taking as reference the smallest sum on study (this includes the baseline sum if that is the smallest on study). In addition to the relative increase of 20%, the sum must also demonstrate an absolute increase of at least 5 mm. (Note: the appearance of one or more new lesions is also considered progression).

2.3.2 Evaluation of non-target lesions

This section provides the definitions of the criteria used to determine the tumor response for the group of non-target lesions. While some non-target lesions may actually be measurable, they need not be measured and instead should be assessed only qualitatively at the time points specified in the protocol.

Table 2. Response criteria for non-target lesions

Response of non-target lesions	Definition
Complete response (CR)	Disappearance of all non-target lesions and normalization of tumor marker level. All lymph nodes must be non-pathological in size (<10 mm short axis).
Non-CR/Non-PD	Persistence of one or more non-target lesion(s) and/or maintenance of tumor marker level above the normal limits.
Progressive Disease (PD)	Unequivocal progression of existing non-target lesions. (Note: the appearance of one or more new lesions is also considered progression).

2.3.3 New lesions

The appearance of new malignant lesions denotes disease progression; Therefore, some comments on detection of new lesions are important. There are no specific criteria for the identification of new radiographic lesions; However, the finding of a new lesion should be unequivocal: i.e. not attributable to differences in scanning technique, change in imaging modality or findings thought to represent something other than tumor (for example, some ‘new’ bone lesions may be simply healing or flare of pre-existing lesions). This is particularly important when the patient’s baseline lesions show partial or complete response. For example, necrosis of a liver lesion may be reported on a CT scan report as a ‘new’ cystic lesion, which it is not. A lesion identified on a study in an anatomical location that was not scanned at baseline is considered a new lesion and will indicate disease progression (PD).
If a new lesion is equivocal (for example, because of its small size), continued therapy and

follow-up evaluation will clarify if it represents truly new disease. If repeat scans confirm there is definitely a new lesion, then progression should be declared using the date of the initial scan.

Table 3. Time Point Response

Target lesions	Non-target lesions	New lesions	Overall response
CR	CR	No	CR
CR	Non-CR/non-PD	No	PR
CR	Not evaluated	No	PR
PR	Non-PD or not all evaluated	No	PR
SD	Non-PD or not all evaluated	No	SD
Not all evaluated	Non-PD	No	NE
PD	Any	Yes or No	PD
Any	PD	Yes or No	PD
Any	Any	Yes	PD

CR: complete response, PR: partial response, SD: stable disease, PD: progressive disease,
NE: invaluable

Table 4. Best overall response when confirmation of CR and PR required

Overall response at first time point	Overall response at subsequent time point	Best overall response
CR	CR	CR
CR	PR	SD, PD or PR[a]
CR	SD	SD provided minimum criteria for SD duration met, otherwise, PD
CR	PD	SD provided minimum criteria for SD duration met, otherwise, PD
CR	NE	SD provided minimum criteria for SD duration met, otherwise NE
PR	CR	PR
PR	PR	PR
PR	SD	SD
PR	PD	SD provided minimum criteria for SD duration met, otherwise, PD
PR	NE	SD provided minimum criteria for SD duration met, otherwise NE
NE	NE	NE

CR: complete response, PR: partial response, SD: stable disease, PD: progressive disease,
NE: invaluable

[a] If a CR is truly met at first time point, then any disease seen at a subsequent time point, even disease meeting PR criteria relative to baseline, makes the disease PD at that point (since disease must have reappeared after CR). Best response would depend on whether minimum duration for SD was met. However, sometimes 'CR' may be claimed when subsequent scans suggest small lesions were likely still present and in fact the patient had PR, not CR at the first time point. Under these circumstances, the original CR should be changed to PR and the best response is PR.

Appendix 3. Response Assessment in Neuro-Oncology (RANO)

Reference: Wen PY, Macdonald R, Reardon DA, et al. Updated Response Assessment Criteria for High-Grade Gliomas: Response Assessment in Neuro-Oncology Working Group, J Clin Oncol 2010; 28 (11):1963-1972

Response	Criteria (RANO 2010)
Complete response (CR)	• Complete disappearance of all enhancing measurable and nonmeasurable disease sustained for at least 1 month<br>• Stable or improved FLAIR/T2 lesions<br>• No new lesions<br>• Stable or improved clinically<br>• No corticosteroids (Physiologic replacement dose is allowable.)
Partial response (PR)	• ≥50% decrease of all enhancing measurable and nonmeasurable lesions sustained for at least 1 month (maximum cross-sectional area of tumors)<br>• No progression of non-measurable disease<br>• No new lesions<br>• Stable or improved FLAIR/T2 lesions<br>• Stable or improved clinically<br>• Same or lower dose of corticosteroids compared with baseline scan
Stable disease (SD)	• Stable FLAIR/T2 lesions on same or lower dose of corticosteroids compared with baseline scan<br>• Clinically stable status
Progression (PD)	• ≥ 25% increase in all enhancing measurable lesions compared with the smallest tumor measurement obtained either at baseline or best response after initiation of therapy, on stable or increasing doses of corticosteroids<br>• Significant increase in FLAIR/T2 lesions on stable or increasing doses of corticosteroids compared with baseline scan or best response after initiation of therapy, not caused by comorbid events (e.g., radiotherapy, ischemic injury, seizures, postoperative changes, or other treatment effects)<br>• Any new lesions<br>• Clinical deterioration not attributable to other causes apart from the tumor or reduction in corticosteroid dose<br>• Death or deteriorating condition<br>• Clear progression of nonmeasurable disease

Appendix 4. RECIL (2017)

Reference: Younes A, Hilden P, Coiffier B, et al. International Working Group consensus response evaluation criteria in lymphoma (RECIL 2017). Annals of Oncology 2017;28:1436-1447

19. ATTACHMENTS

Attachment 1. Protocol Agreement

Attachment 2. Investigational Site, and Names and Titles of Principal Investigator· Subinvestigators · Clinical Trial Pharmacists

Attachment 3. Patient Information Sheet and Informed Consent Form

Attachment 4. Compensation policy for victims
