# Supplementary material for: First-in-human study of IM156, a novel potent biguanide oxidative phosphorylation (OXPHOS) inhibitor, in patients with advanced solid tumors
Source: Invest New Drugs. 2022 Jul 8;40(5):1001–10. doi: 10.1007/s10637-022-01277-9 (PMC9395488; doi:10.1007/s10637-022-01277-9)

**SUPPLEMENTARY TABLES AND FIGURES**

**Supplementary Table 1.** All Treatment-Emergent Adverse Events

| **Adverse Events /Grades** | **IM156 orally every other day** | | | | | | | | | | | | | | | | | | | | **IM156 orally daily** | | | | | | | | **Total** | | | | |
| --- | --- | --- | --- | --- | --- | --- | --- | --- | --- | --- | --- | --- | --- | --- | --- | --- | --- | --- | --- | --- | --- | --- | --- | --- | --- | --- | --- | --- | --- | --- | --- | --- | --- |
|  | **DL1 100 mg (n=3)** | | | | **DL2 200 mg (n=3)** | | | | **DL3 400 mg (n=3)** | | | | **DL4 800 mg (n=3)** | | | | **DL5 1200 mg (n=3)** | | | | **DL6 800 mg (n=3)** | | | | **DL7 1200 mg (n=4)** | | | |  | | | | |
|  | **1** | **2** | **3** | **4** | **1** | **2** | **3** | **4** | **1** | **2** | **3** | **4** | **1** | **2** | **3** | **4** | **1** | **2** | **3** | **4** | **1** | **2** | **3** | **4** | **1** | **2** | **3** | **4** | **1** | **2** | | **3** | **4** |
| **Gastrointestinal disorders** | | | | | | | | | | | | | | | | | | | | | | | | | | | | | | | | | |
| Nausea | 1 | 0 | 0 | 0 | 1 | 0 | 0 | 0 | 1 | 2 | 0 | 0 | 2 | 1 | 0 | 0 | 1 | 0 | 1 | 0 | 2 | 0 | 0 | 0 | 0 | 2 | 2 | 0 | 8 | 5 | 3 | | 0 |
| Diarrhoea | 0 | 1 | 0 | 0 | 0 | 1 | 0 | 0 | 0 | 0 | 0 | 0 | 3 | 0 | 0 | 0 | 1 | 1 | 0 | 0 | 3 | 0 | 0 | 0 | 2 | 0 | 0 | 0 | 9 | 3 | 0 | | 0 |
| Vomiting | 1 | 0 | 0 | 0 | 2 | 0 | 0 | 0 | 2 | 0 | 0 | 0 | 0 | 0 | 0 | 0 | 1 | 1 | 0 | 0 | 1 | 0 | 0 | 0 | 3 | 0 | 0 | 0 | 10 | 1 | 0 | | 0 |
| Abdominal pain | 0 | 0 | 0 | 0 | 0 | 1 | 0 | 0 | 0 | 0 | 0 | 0 | 1 | 0 | 1 | 0 | 1 | 0 | 0 | 0 | 0 | 0 | 0 | 0 | 0 | 2 | 0 | 0 | 2 | 3 | 1 | | 0 |
| Constipation | 0 | 0 | 0 | 0 | 0 | 0 | 0 | 0 | 0 | 0 | 0 | 0 | 1 | 1 | 0 | 0 | 1 | 0 | 0 | 0 | 2 | 0 | 0 | 0 | 1 | 0 | 0 | 0 | 5 | 1 | 0 | | 0 |
| Gastrointestinal pain | 0 | 1 | 0 | 0 | 0 | 0 | 0 | 0 | 0 | 0 | 0 | 0 | 0 | 0 | 0 | 0 | 0 | 0 | 0 | 0 | 1 | 0 | 0 | 0 | 0 | 0 | 0 | 0 | 1 | 1 | 0 | | 0 |
| Ileus | 0 | 0 | 0 | 0 | 0 | 0 | 1 | 0 | 0 | 0 | 0 | 0 | 0 | 0 | 0 | 0 | 0 | 0 | 0 | 0 | 0 | 0 | 0 | 0 | 0 | 1 | 0 | 0 | 0 | 1 | 1 | | 0 |
| Abdominal discomfort | 0 | 0 | 0 | 0 | 0 | 0 | 0 | 0 | 0 | 0 | 0 | 0 | 0 | 0 | 0 | 0 | 0 | 0 | 0 | 0 | 1 | 0 | 0 | 0 | 0 | 0 | 0 | 0 | 1 | 0 | 0 | | 0 |
| Abdominal distension | 0 | 1 | 0 | 0 | 0 | 0 | 0 | 0 | 0 | 0 | 0 | 0 | 0 | 0 | 0 | 0 | 0 | 0 | 0 | 0 | 0 | 0 | 0 | 0 | 0 | 0 | 0 | 0 | 0 | 1 | 0 | | 0 |
| Abdominal pain upper | 0 | 0 | 0 | 0 | 0 | 0 | 0 | 0 | 0 | 0 | 0 | 0 | 0 | 1 | 0 | 0 | 0 | 0 | 0 | 0 | 0 | 0 | 0 | 0 | 0 | 0 | 0 | 0 | 0 | 1 | 0 | | 0 |
| Ascites | 1 | 0 | 0 | 0 | 0 | 0 | 0 | 0 | 0 | 0 | 0 | 0 | 0 | 0 | 0 | 0 | 0 | 0 | 0 | 0 | 0 | 0 | 0 | 0 | 0 | 0 | 0 | 0 | 1 | 0 | 0 | | 0 |
| Gastritis | 0 | 0 | 0 | 0 | 0 | 0 | 0 | 0 | 0 | 0 | 0 | 0 | 0 | 0 | 0 | 0 | 0 | 0 | 0 | 0 | 0 | 0 | 0 | 0 | 1 | 0 | 0 | 0 | 1 | 0 | 0 | | 0 |
| Large intestinal obstruction | 0 | 1 | 0 | 0 | 0 | 0 | 0 | 0 | 0 | 0 | 0 | 0 | 0 | 0 | 0 | 0 | 0 | 0 | 0 | 0 | 0 | 0 | 0 | 0 | 0 | 0 | 0 | 0 | 0 | 1 | 0 | | 0 |
| Obstruction gastric | 0 | 0 | 1 | 0 | 0 | 0 | 0 | 0 | 0 | 0 | 0 | 0 | 0 | 0 | 0 | 0 | 0 | 0 | 0 | 0 | 0 | 0 | 0 | 0 | 0 | 0 | 0 | 0 | 0 | 0 | 1 | | 0 |
| Proctalgia | 0 | 1 | 0 | 0 | 0 | 0 | 0 | 0 | 0 | 0 | 0 | 0 | 0 | 0 | 0 | 0 | 0 | 0 | 0 | 0 | 0 | 0 | 0 | 0 | 0 | 0 | 0 | 0 | 0 | 1 | 0 | | 0 |
| Toothache | 0 | 0 | 0 | 0 | 0 | 0 | 0 | 0 | 0 | 0 | 0 | 0 | 0 | 0 | 0 | 0 | 0 | 0 | 0 | 0 | 1 | 0 | 0 | 0 | 0 | 0 | 0 | 0 | 1 | 0 | 0 | | 0 |
| **General disorders and administration site conditions** | | | | | | | | | | | | | | | | | | | | | | | | | | | | | | | | | |
| Fatigue | 0 | 0 | 0 | 0 | 0 | 0 | 0 | 0 | 0 | 0 | 0 | 0 | 1 | 0 | 0 | 0 | 0 | 1 | 0 | 0 | 1 | 0 | 0 | 0 | 0 | 3 | 0 | 0 | 2 | 4 | 0 | | 0 |
| Pyrexia | 1 | 0 | 0 | 0 | 1 | 0 | 0 | 0 | 0 | 0 | 0 | 0 | 0 | 0 | 0 | 0 | 1 | 0 | 0 | 0 | 0 | 0 | 0 | 0 | 2 | 1 | 0 | 0 | 5 | 1 | 0 | | 0 |
| Asthenia | 0 | 0 | 0 | 0 | 1 | 0 | 0 | 0 | 0 | 0 | 0 | 0 | 1 | 0 | 0 | 0 | 1 | 0 | 0 | 0 | 0 | 0 | 0 | 0 | 0 | 0 | 0 | 0 | 3 | 0 | 0 | | 0 |
| Chest pain | 0 | 0 | 0 | 0 | 0 | 0 | 0 | 0 | 0 | 0 | 0 | 0 | 1 | 0 | 0 | 0 | 0 | 0 | 0 | 0 | 0 | 0 | 0 | 0 | 0 | 0 | 0 | 0 | 1 | 0 | 0 | | 0 |
| Influenza like illness | 0 | 0 | 0 | 0 | 0 | 0 | 0 | 0 | 0 | 0 | 0 | 0 | 0 | 0 | 0 | 0 | 0 | 0 | 0 | 0 | 0 | 0 | 0 | 0 | 1 | 0 | 0 | 0 | 1 | 0 | 0 | | 0 |
| Oedema peripheral | 0 | 0 | 0 | 0 | 0 | 0 | 0 | 0 | 0 | 0 | 0 | 0 | 0 | 0 | 0 | 0 | 0 | 0 | 0 | 0 | 0 | 0 | 0 | 0 | 0 | 1 | 0 | 0 | 0 | 1 | 0 | | 0 |
| Pain | 0 | 0 | 0 | 0 | 0 | 0 | 0 | 0 | 0 | 0 | 0 | 0 | 0 | 0 | 0 | 0 | 0 | 0 | 0 | 0 | 0 | 0 | 0 | 0 | 0 | 1 | 0 | 0 | 0 | 1 | 0 | | 0 |
| **Investigations** | | | | | | | | | | | | | | | | | | | | | | | | | | | | | | | | | |
| Alanine aminotransferase increased | 0 | 0 | 0 | 0 | 1 | 0 | 0 | 0 | 0 | 0 | 0 | 0 | 0 | 0 | 0 | 0 | 0 | 0 | 1 | 0 | 1 | 0 | 0 | 0 | 1 | 0 | 0 | 0 | 3 | 0 | 1 | | 0 |
| Aspartate aminotransferase increased | 0 | 0 | 0 | 0 | 0 | 0 | 0 | 0 | 0 | 0 | 0 | 0 | 0 | 0 | 0 | 0 | 0 | 1 | 1 | 0 | 1 | 0 | 0 | 0 | 1 | 0 | 0 | 0 | 2 | 1 | 1 | | 0 |
| Blood creatinine increased | 1 | 0 | 0 | 0 | 0 | 0 | 0 | 0 | 0 | 0 | 0 | 0 | 0 | 0 | 0 | 0 | 0 | 0 | 0 | 0 | 0 | 0 | 0 | 0 | 2 | 0 | 0 | 0 | 3 | 0 | 0 | | 0 |
| Blood alkaline phosphatase increased | 0 | 0 | 0 | 0 | 0 | 0 | 0 | 0 | 0 | 0 | 0 | 0 | 0 | 0 | 0 | 0 | 0 | 1 | 1 | 0 | 0 | 0 | 0 | 0 | 0 | 0 | 0 | 0 | 0 | 1 | 1 | | 0 |
| Blood lactic acid increased | 0 | 0 | 0 | 0 | 0 | 0 | 0 | 0 | 0 | 0 | 0 | 0 | 0 | 0 | 0 | 0 | 0 | 0 | 0 | 0 | 1 | 0 | 0 | 0 | 1 | 0 | 0 | 0 | 2 | 0 | 0 | | 0 |
| Gamma-glutamyltransferase increased | 0 | 0 | 0 | 0 | 0 | 0 | 0 | 0 | 0 | 0 | 0 | 0 | 0 | 0 | 0 | 0 | 0 | 1 | 1 | 0 | 0 | 0 | 0 | 0 | 0 | 0 | 0 | 0 | 0 | 1 | 1 | | 0 |
| Neutrophil count decreased | 0 | 0 | 0 | 0 | 0 | 0 | 0 | 0 | 0 | 0 | 0 | 1 | 0 | 0 | 0 | 0 | 0 | 0 | 0 | 0 | 0 | 0 | 0 | 0 | 0 | 0 | 0 | 0 | 0 | 0 | 0 | | 1 |
| Platelet count decreased | 0 | 0 | 0 | 0 | 0 | 0 | 0 | 0 | 0 | 0 | 0 | 0 | 0 | 0 | 0 | 0 | 0 | 0 | 0 | 0 | 0 | 0 | 0 | 0 | 1 | 0 | 0 | 0 | 1 | 0 | 0 | | 0 |
| **Metabolism and nutrition disorders** | | | | | | | | | | | | | | | | | | | | | | | | | | | | | | | | | |
| Decreased appetite | 1 | 0 | 0 | 0 | 0 | 0 | 0 | 0 | 0 | 0 | 0 | 0 | 0 | 0 | 0 | 0 | 1 | 0 | 0 | 0 | 1 | 0 | 0 | 0 | 0 | 1 | 0 | 0 | 3 | 1 | 0 | | 0 |
| Hypoalbuminaemia | 0 | 1 | 0 | 0 | 0 | 0 | 0 | 0 | 0 | 0 | 0 | 0 | 0 | 0 | 0 | 0 | 0 | 1 | 0 | 0 | 0 | 0 | 0 | 0 | 0 | 0 | 0 | 0 | 0 | 2 | 0 | | 0 |
| Hyperkalaemia | 0 | 0 | 0 | 0 | 0 | 0 | 0 | 0 | 0 | 0 | 0 | 0 | 0 | 0 | 0 | 0 | 0 | 0 | 0 | 0 | 0 | 0 | 0 | 0 | 0 | 0 | 1 | 0 | 0 | 0 | 1 | | 0 |
| Hypocalcemia | 0 | 0 | 0 | 0 | 0 | 0 | 0 | 0 | 0 | 0 | 0 | 0 | 0 | 0 | 0 | 0 | 0 | 1 | 0 | 0 | 0 | 0 | 0 | 0 | 0 | 0 | 0 | 0 | 0 | 1 | 0 | | 0 |
| **Nervous system disorders** | | | | | | | | | | | | | | | | | | | | | | | | | | | | | | | | | |
| Headache | 0 | 0 | 0 | 0 | 0 | 0 | 0 | 0 | 0 | 1 | 0 | 0 | 0 | 0 | 0 | 0 | 1 | 0 | 0 | 0 | 0 | 0 | 0 | 0 | 0 | 0 | 0 | 0 | 1 | 1 | 0 | | 0 |
| Akathisia | 0 | 0 | 0 | 0 | 0 | 0 | 0 | 0 | 0 | 0 | 0 | 0 | 0 | 0 | 0 | 0 | 0 | 0 | 0 | 0 | 0 | 0 | 0 | 0 | 0 | 1 | 0 | 0 | 0 | 1 | 0 | | 0 |
| Dysesthesia | 0 | 0 | 0 | 0 | 0 | 0 | 0 | 0 | 0 | 0 | 0 | 0 | 0 | 0 | 0 | 0 | 0 | 0 | 0 | 0 | 0 | 0 | 0 | 0 | 1 | 0 | 0 | 0 | 1 | 0 | 0 | | 0 |
| Lethargy | 0 | 0 | 0 | 0 | 0 | 0 | 0 | 0 | 0 | 0 | 0 | 0 | 0 | 0 | 0 | 0 | 0 | 0 | 0 | 0 | 0 | 0 | 0 | 0 | 0 | 1 | 0 | 0 | 0 | 1 | 0 | | 0 |
| Peripheral sensory neuropathy | 0 | 0 | 0 | 0 | 0 | 0 | 0 | 0 | 1 | 0 | 0 | 0 | 0 | 0 | 0 | 0 | 0 | 0 | 0 | 0 | 0 | 0 | 0 | 0 | 0 | 0 | 0 | 0 | 1 | 0 | 0 | | 0 |
| **Respiratory, thoracic and mediastinal disorders** | | | | | | | | | | | | | | | | | | | | | | | | | | | | | | | | | |
| Cough | 0 | 0 | 0 | 0 | 1 | 0 | 0 | 0 | 1 | 0 | 0 | 0 | 1 | 0 | 0 | 0 | 0 | 0 | 0 | 0 | 0 | 0 | 0 | 0 | 0 | 0 | 0 | 0 | 3 | 0 | 0 | | 0 |
| Hemoptysis | 0 | 0 | 0 | 0 | 0 | 0 | 0 | 0 | 0 | 0 | 0 | 0 | 0 | 0 | 0 | 0 | 0 | 0 | 0 | 0 | 0 | 0 | 0 | 0 | 0 | 1 | 0 | 0 | 0 | 1 | 0 | | 0 |
| Oropharyngeal pain | 0 | 0 | 0 | 0 | 0 | 0 | 0 | 0 | 0 | 0 | 0 | 0 | 0 | 0 | 0 | 0 | 1 | 0 | 0 | 0 | 0 | 0 | 0 | 0 | 0 | 0 | 0 | 0 | 1 | 0 | 0 | | 0 |
| Pleural effusion | 0 | 0 | 0 | 0 | 0 | 1 | 0 | 0 | 0 | 0 | 0 | 0 | 0 | 0 | 0 | 0 | 0 | 0 | 0 | 0 | 0 | 0 | 0 | 0 | 0 | 0 | 0 | 0 | 0 | 1 | 0 | | 0 |
| **Blood and lymphatic system disorders** | | | | | | | | | | | | | | | | | | | | | | | | | | | | | | | | | |
| Anaemia | 0 | 1 | 0 | 0 | 0 | 1 | 0 | 0 | 0 | 0 | 0 | 0 | 0 | 0 | 0 | 0 | 0 | 0 | 1 | 0 | 0 | 0 | 0 | 0 | 0 | 1 | 1 | 0 | 0 | 3 | 2 | | 0 |
| **Musculoskeletal and connective tissue disorders** | | | | | | | | | | | | | | | | | | | | | | | | | | | | | | | | |  |
| Back pain | 0 | 0 | 0 | 0 | 0 | 0 | 0 | 0 | 0 | 0 | 0 | 0 | 0 | 2 | 0 | 0 | 0 | 0 | 0 | 0 | 0 | 0 | 0 | 0 | 0 | 0 | 0 | 0 | 0 | 2 | 0 | | 0 |
| Pain in extremity | 0 | 0 | 0 | 0 | 0 | 0 | 0 | 0 | 1 | 0 | 0 | 0 | 0 | 1 | 0 | 0 | 0 | 0 | 0 | 0 | 0 | 0 | 0 | 0 | 0 | 0 | 0 | 0 | 1 | 1 | 0 | | 0 |
| Flank pain | 0 | 0 | 0 | 0 | 0 | 1 | 0 | 0 | 0 | 0 | 0 | 0 | 0 | 0 | 0 | 0 | 0 | 0 | 0 | 0 | 0 | 0 | 0 | 0 | 0 | 0 | 0 | 0 | 0 | 1 | 0 | | 0 |
| **Psychiatric disorders** | 0 | 0 | 0 | 0 | 0 | 1 | 0 | 0 | 1 | 0 | 0 | 0 | 0 | 0 | 0 | 0 | 0 | 0 | 0 | 0 | 1 | 0 | 0 | 0 | 0 | 0 | 0 | 0 | 2 | 1 | 0 | | 0 |
| Insomnia | 0 | 0 | 0 | 0 | 0 | 1 | 0 | 0 | 1 | 0 | 0 | 0 | 0 | 0 | 0 | 0 | 0 | 0 | 0 | 0 | 1 | 0 | 0 | 0 | 0 | 0 | 0 | 0 | 2 | 1 | 0 | | 0 |
| **Skin and subcutaneous tissue disorders** | | | | | | | | | | | | | | | | | | | | | | | | | | | | | | | | | |
| Dry skin | 0 | 0 | 0 | 0 | 0 | 0 | 0 | 0 | 0 | 0 | 0 | 0 | 0 | 0 | 0 | 0 | 0 | 0 | 0 | 0 | 0 | 0 | 0 | 0 | 0 | 1 | 0 | 0 | 0 | 1 | 0 | | 0 |
| Pruritus | 0 | 0 | 0 | 0 | 0 | 0 | 0 | 0 | 0 | 0 | 0 | 0 | 0 | 0 | 0 | 0 | 0 | 0 | 0 | 0 | 0 | 0 | 0 | 0 | 1 | 0 | 0 | 0 | 1 | 0 | 0 | | 0 |
| Purpura | 0 | 0 | 0 | 0 | 0 | 0 | 0 | 0 | 0 | 0 | 0 | 0 | 0 | 0 | 0 | 0 | 0 | 0 | 0 | 0 | 0 | 0 | 0 | 0 | 1 | 0 | 0 | 0 | 1 | 0 | 0 | | 0 |
| **Infections and infestations** | | | | | | | | | | | | | | | | | | | | | | | | | | | | | | | | | |
| Appendicitis perforated | 0 | 0 | 0 | 0 | 0 | 0 | 0 | 0 | 0 | 0 | 0 | 0 | 0 | 0 | 0 | 0 | 0 | 0 | 0 | 0 | 0 | 0 | 0 | 0 | 0 | 0 | 1 | 0 | 0 | 0 | 1 | | 0 |
| Cystitis | 0 | 0 | 0 | 0 | 0 | 0 | 0 | 0 | 0 | 0 | 0 | 0 | 0 | 1 | 0 | 0 | 0 | 0 | 0 | 0 | 0 | 0 | 0 | 0 | 0 | 0 | 0 | 0 | 0 | 1 | 0 | | 0 |
| **Renal and urinary disorders** | | | | | | | | | | | | | | | | | | | | | | | | | | | | | | | | | |
| Urinary retention | 0 | 0 | 0 | 0 | 0 | 0 | 0 | 0 | 0 | 0 | 0 | 0 | 0 | 1 | 0 | 0 | 0 | 0 | 0 | 0 | 0 | 0 | 0 | 0 | 0 | 0 | 0 | 0 | 0 | 1 | 0 | | 0 |
| Urinary tract disorder | 0 | 0 | 0 | 0 | 0 | 0 | 0 | 0 | 0 | 0 | 0 | 0 | 0 | 0 | 0 | 0 | 0 | 0 | 0 | 0 | 0 | 0 | 0 | 0 | 0 | 1 | 0 | 0 | 0 | 1 | 0 | | 0 |
| **Vascular disorders** | | | | | | | | | | | | | | | | | | | | | | | | | | | | | | | | | |
| Hypertension | 0 | 0 | 0 | 0 | 0 | 0 | 0 | 0 | 0 | 1 | 0 | 0 | 0 | 0 | 0 | 0 | 0 | 0 | 0 | 0 | 0 | 0 | 0 | 0 | 0 | 0 | 0 | 0 | 0 | 1 | 0 | | 0 |
| Hypotension | 0 | 0 | 0 | 0 | 0 | 0 | 0 | 0 | 0 | 0 | 0 | 0 | 0 | 0 | 0 | 0 | 0 | 1 | 0 | 0 | 0 | 0 | 0 | 0 | 0 | 0 | 0 | 0 | 0 | 1 | 0 | | 0 |
| **Endocrine disorders** | | | | | | | | | | | | | | | | | | | | | | | | | | | | | | | | | |
| Adrenal insufficiency | 0 | 0 | 0 | 0 | 0 | 0 | 0 | 0 | 0 | 0 | 0 | 0 | 0 | 0 | 0 | 0 | 0 | 1 | 0 | 0 | 0 | 0 | 0 | 0 | 0 | 0 | 0 | 0 | 0 | 1 | 0 | | 0 |

Abbreviations: DL, dose level

**Supplementary Table 2.** Serious adverse events.

| **Dose** | **Adverse Event** | **Grade** | **Dose Interrupted** | **Outcome** | **Causality** |
| --- | --- | --- | --- | --- | --- |
| 100 mg QOD | Gastric Obstruction | 3 | Yes | Recovered without sequelae | Unlikely |
| 100 mg QOD | Abdominal distension | 2 | Yes | Recovered without sequelae | Unlikely |
| 200 mg QOD | Ileus | 3 | No | Recovered without sequelae | Unlikely |
| 800 mg QOD | Cystitis | 2 | Yes | Recovered without sequelae | Unlikely |
| 800 mg QOD | Worsen abdominal pain | 3 | Yes | Resolved to Grade 1 | Unlikely |
| 1,200 mg QOD | Nausea | 3 | Yes | Resolved to Grade 12 | Possible |
| 1,200 mg QD | Lethargy | 2 | Yes | Recovered without sequelae | Unlikely |
| 1,200 mg QD | Akathisia | 2 | No | Recovered without sequelae | Unlikely |
| 1,200 mg QD | Perforated Appendicitis | 3 | No | Recovered without sequelae | Unlikely |

**Supplementary Table 3. Pharmacokinetics of IM156 on Days 1 and 27 of Cycle 1 in Urine**.

| **Day** | **Cohort** | **Regimen** | **Dose (mg)** | **Total Amount Excreted (A_e_) (µg)** | **Plasma AUC_0-24_**  **(h*ng/mL)** | **CLr**  **(L/h)** | **% Dose Recovered** |
| --- | --- | --- | --- | --- | --- | --- | --- |
| 1 | 1 | QOD | 100 | 1790 ± 620 | 482 ± 342 | 6.16 ± 5.25 | 1.79 ± 0.620 |
|  | 2 | QOD | 200 | 20000 ± 3150 | 2980 ± 251 | 6.69 ± 0.709 | 10.0 ± 1.57 |
|  | 3 | QOD | 400 | 31600 ± 11600 | 4380 ± 3010 | 9.41 ± 6.80 | 7.90 ± 2.90 |
|  | 4 | QOD | 800 | 62900 ± 34000 | 7250 ± 3960 | 10.4 ± 6.93 | 7.86 ± 4.25 |
|  | 5 | QOD | 1200 | 109000 ± 155000 | 10600 ± 4820 | 7.89 ± 9.01 | 9.11 ± 12.9 |
|  | 6 | QD | 800 | 63700 ± 47500 | 7790 ± 4530 | 7.92 ± 2.62 | 7.96 ± 5.94 |
|  | 7 | QD | 1200 | 105000 ± 24600 | 16800 ± 7720 | 7.60 ± 4.02 | 8.79 ± 2.05 |
| Fed 1 | 7 | QD | 1200 | 65100 ± 42100 | 17600 ± 12800 | 5.16 ± 3.51 | 5.42 ± 3.51 |
| 27 | 1 | QOD | 100 | 4320 | 1070 | 4.46 | 4.32 |
|  | 2 | QOD | 200 | 27700 ± 4680 | 4200 ± 1140 | 6.83 ± 1.58 | 13.9 ± 2.34 |
|  | 3 | QOD | 400 | 64700 ± 45600 | 7630 ± 5410 | 8.68 ± 1.52 | 16.2 ± 11.4 |
|  | 4 | QOD | 800 | 129000 ± 78400 | 12900 ± 9470 | 11.2 ± 6.94 | 16.2 ± 9.80 |
|  | 5 | QOD | 1200 | 225000 ± 128000 | 21800 ± 3680 | 10.9 ± 7.79 | 18.7 ± 10.6 |
|  | 6 | QD | 800 | 279000 ± 52500 | 33600 ± 9780 | 8.72 ± 3.01 | 34.8 ± 6.56 |
|  | 7 | QD | 1200 | 252000 | 36300 | 7.16 | 21.0 |
| Values represent Mean ± SD, except for n<3 | | | | | | | |

**Supplementary Figure 1.** Inhibition effect of IM156-M5 on oxygen consumption rate in A549 human lung carcinoma cells.

**
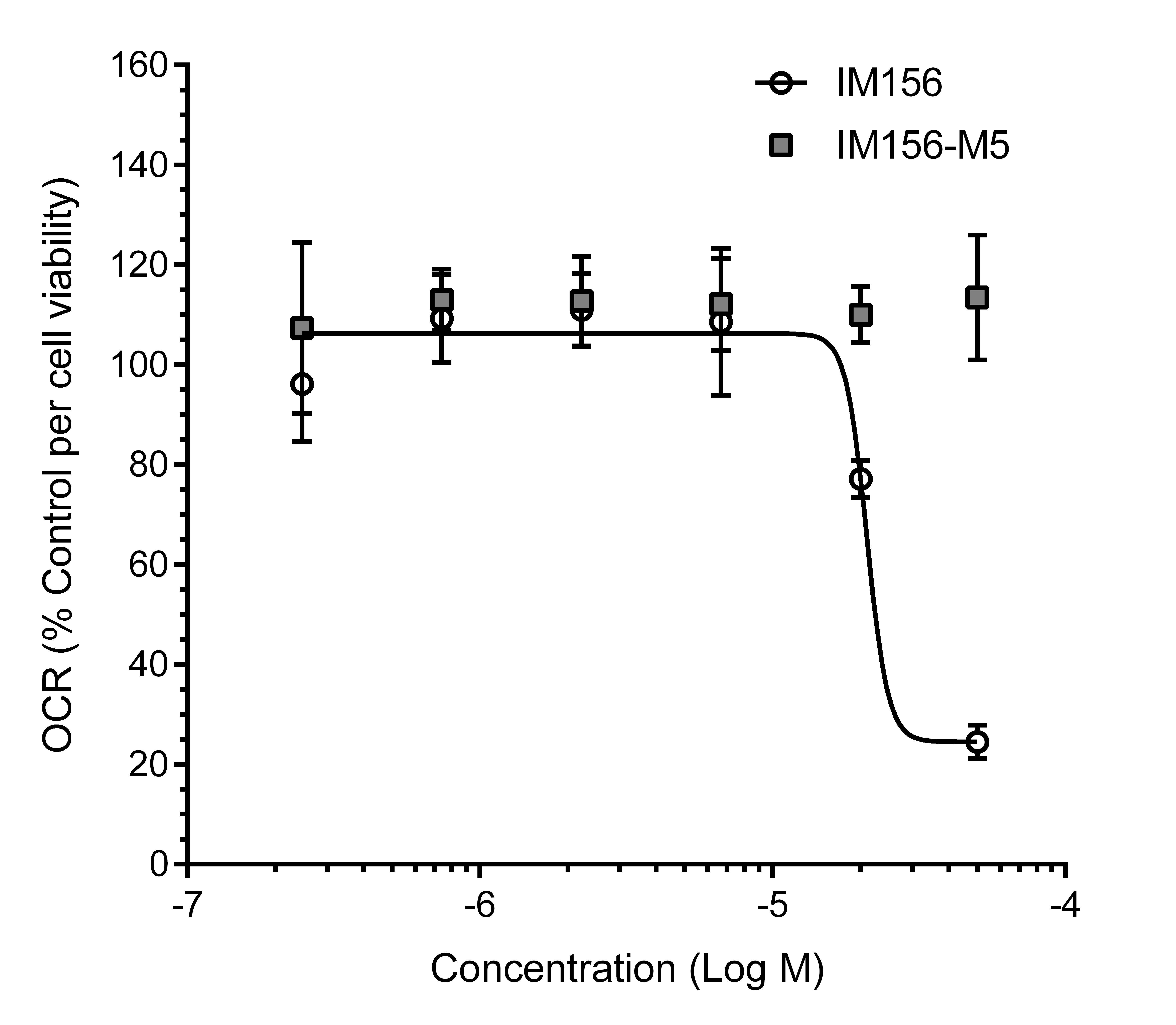
**

**Supplementary Figure 2.** Progression-free survival.


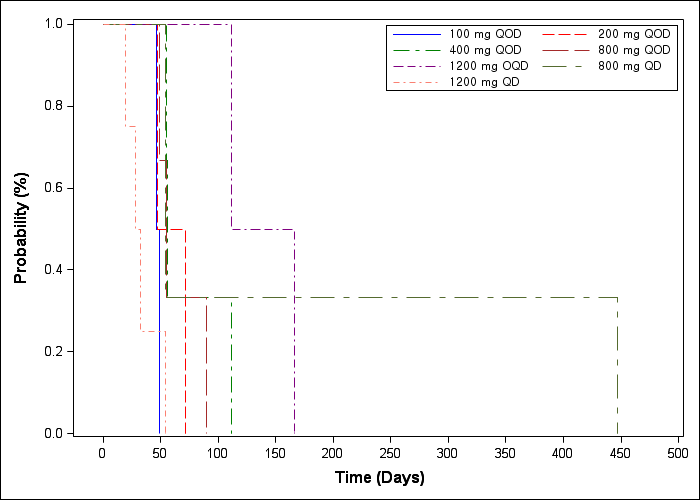

Supplement: Supplementary file 2 — Supplementary file1 (DOCX 780 KB) [file 10637_2022_1277_MOESM2_ESM.docx]
